# Are Healthcare Workers at an Increased Risk for Obstructive Respiratory Diseases Due to Cleaning and Disinfection Agents? A Systematic Review and Meta-Analysis

**DOI:** 10.3390/ijerph18105159

**Published:** 2021-05-13

**Authors:** Karla Romero Starke, Sophie Friedrich, Melanie Schubert, Daniel Kämpf, Maria Girbig, Anna Pretzsch, Albert Nienhaus, Andreas Seidler

**Affiliations:** 1 Institute and Policlinic of Occupational and Social Medicine (IPAS), Faculty of Medicine Carl Gustav Carus, Technische Universität Dresden, 01307 Dresden, Germany; sophie.wagner1@tu-dresden.de (S.F.); melanie.schubert@tu-dresden.de (M.S.); Daniel.Kaempf@mailbox.tu-dresden.de (D.K.); Maria.Girbig@mailbox.tu-dresden.de (M.G.); anna.pretzsch@mailbox.tu-dresden.de (A.P.); andreas.seidler@mailbox.tu-dresden.de (A.S.); 2 Institute of Sociology, Faculty of Behavioral and Social Sciences, Chemnitz University of Technology, Thüringer Weg 9, 09126 Chemnitz, Germany; 3 Department of Occupational Medicine, Toxic Substances and Health Research, Institution for Statutory Social Accident Insurance and Prevention in the Health Care and Welfare Services (BGW), 22089 Hamburg, Germany; Albert.Nienhaus@bgw-online.de; 4 Competence Centre for Epidemiology and Health Services Research for Healthcare Professionals (CVcare), Institute for Health Service Research in Dermatology and Nursing (IVDP), University Medical Centre Hamburg-Eppendorf (UKE), 20251 Hamburg, Germany

**Keywords:** nurses, healthcare workers, clean, disinfect, glutaraldehyde, bleach, asthma, obstructive respiratory disease, bronchial hyperresponsiveness, COPD

## Abstract

Several reviews have reported an increased risk of obstructive respiratory diseases in workers exposed to cleaning or disinfection agents, but they have focused mainly on professional cleaners. Cleaning and disinfecting are frequently performed activities by healthcare workers. We conducted a systematic review with meta-analysis to quantify the risk of obstructive respiratory diseases in healthcare workers exposed to cleaning and disinfection agents. We searched the Medline and Embase databases until 4 February 2021 to find adequate primary studies. Two independent reviewers screened the titles/abstracts and the full texts of the studies, as well as performing data extraction and quality assessment. The literature search yielded 9432 records, and 8 studies were found through a hand search. After screening, 14 studies were included in the review. All had a high risk of bias, and most studies dealt with nurses, asthma, and hyperresponsiveness (BHR)-related symptoms. Only one study investigated COPD. The meta-analysis estimated an increased risk of new-onset asthma for nurses (Effect size (ES) = 1.67; 95% CI 1.11–2.50) compared with other occupations and found an increase in the risk of new-onset asthma for nurses exposed to cleaning and disinfecting surfaces (ES = 1.43; 95% CI 1.09–1.89) and instruments (ES = 1.34; 95% CI 1.09–1.65). Exposure to specific chemicals such as bleach and glutaraldehyde (GA) increased the risk of asthma in nurses (bleach ES = 2.44; 95% CI 1.56–3.82; GA ES = 1.91, 95% CI 1.35–2.70). A higher risk for BHR-related symptoms was observed for nurses exposed to cleaning surfaces (ES = 1.44; 95% CI 1.18–1.78). Although the overall evidence was rated as low, the limitations found in this review hint at a potential underestimation of the real risk. These findings highlight the need for reinforced prevention practices with regard to healthcare workers. Similar research investigating these associations among other healthcare workers such as rescue service and nursing home personnel is needed.

## 1. Introduction

Nursing and caring professionals constitute a large part of the working population. In Germany, there are approximately 1.1 million people working in one of these healthcare fields, constituting 3.2% of the workforce subject to social security [1,2]. In the European Union, the share of nurses and midwives in the total workforce on average was 2.2% in 2019, and the absolute numbers have been rising over the past years [3]. The rise in this occupational group of approximately 11% from 2011 to 2019 has outpaced the increase in the general population in the European Union during the same time frame [4].

Cleaning and disinfection tasks are a significant part of nursing professionals’ duties, with the majority being exposed to chemical agents needed for this purpose [5,6]. Beyond nurses, other healthcare workers such as rescue service workers are also exposed to cleaning and disinfection agents: in accordance with the framework hygiene plan for rescue and patient transport services, surfaces in contact with patients are disinfected after each deployment, and the emergency vehicle is thoroughly cleaned at least once a week [7]. The exposure to some of these agents, such as formaldehyde, glutaraldehyde (GA), quaternary ammonium compounds (QAC), and chloramine-T, has been shown to cause asthma [8,9]. The mechanisms behind this process are still not clear, but it is likely that both allergic and irritant mechanisms are involved [10]. It is thus reasonable to suspect that healthcare workers are at a higher risk for obstructive respiratory diseases than the general population.

Indeed, several reviews investigating the risk of respiratory diseases in the general working population and in cleaners due to exposure to cleaning and disinfecting agents have been published [11,12,13,14]. These studies have found a higher risk for professional cleaners compared with other nonexposed working populations. However, to our knowledge, there has not been a systematic review focusing on the risk of obstructive respiratory diseases in healthcare personnel. Such a review can result in prevention strategies to minimize the occurrence of occupational or work-related asthma among this professional group.

### Aims and Objectives

We conducted a systematic review in order to determine whether healthcare workers with exposure to cleaning or disinfecting agents had an elevated risk of developing obstructive respiratory diseases compared with a nonexposed comparison group. Further, we aimed to quantify the risk due to these exposures by meta-analytical methods.

## 2. Methods

### 2.1. Protocol and Registration

Our review on respiratory diseases in healthcare workers was written considering the guidelines for conducting and reporting meta-analyses of observational studies in epidemiology (MOOSE) [15] and the Preferred Reporting Items for Systematic reviews and Meta-Analysis (PRISMA) [16]. The study protocol was registered in PROSPERO under record number CRD42019139699 and is available at https://www.crd.york.ac.uk/prospero/display_record.php?RecordID=139699 (accessed on 4 February 2021).

### 2.2. Eligibility Criteria

We designed our systematic search strategy based on the Population, Exposure, Control/Comparison, Outcome, and Study Design (PECOS) scheme [17] (Table 1). We included studies of employable populations of both sexes, between 16 and 70 years old (P), and excluded studies of the non-employable population, of adolescents and children under 16 years of age, and of elderly people over 70 years of age.

The ideal study would evaluate only nurses or other healthcare workers such as rescue service personnel with known exposure to cleaning or disinfection agents and compare them with otherwise similar workers in other occupational groups without any elevated risk for respiratory diseases due to other exposures (i.e., dusts, chemical irritants, allergenic substances). However, we also considered other study designs, such as studies using an internal comparison group (i.e., comparing nurses with and without exposure to cleaning or disinfectant agents). We therefore divided the included studies into Type A or Type B. For occupation-based “Type A” studies, we included studies investigating employment as a healthcare worker, in which at least a high percentage of the nurses were confirmed to be exposed to cleaning or disinfectant agents. “Type A” studies must have used an external comparison population employed in other occupations where an average risk for respiratory diseases due to other exposures can be assumed, as described above. For exposure-based “Type B” studies, we included studies investigating the risk of exposure to cleaning or disinfectant agents in which healthcare workers were at least an important part of the exposed population, evaluated against either an internal (i.e., other nurses not exposed to cleaning or disinfectant agents) comparison group or an appropriate external comparison group (i.e., office workers).

Studies assessing asthma, chronic obstructive pulmonary disease (COPD), or other obstructive diseases were included, as well as studies assessing lung function abnormalities. We included cohort, case-control, and cross-sectional study designs and excluded editorials, qualitative studies, studies only having abstracts, conference papers/posters, reviews, and letters. The four last types of publications were excluded because their information may have been preliminary. Furthermore, we excluded studies with a convenience sample, those not reporting response, or those with a response less than 10% to avoid studies with a potentially strong selection bias. Only studies published since 1990 in Europe, US, Canada, and Australia were considered in order to achieve comparable population data. We applied no language restrictions.

### 2.3. Information Sources and Search

**Figure 1:**
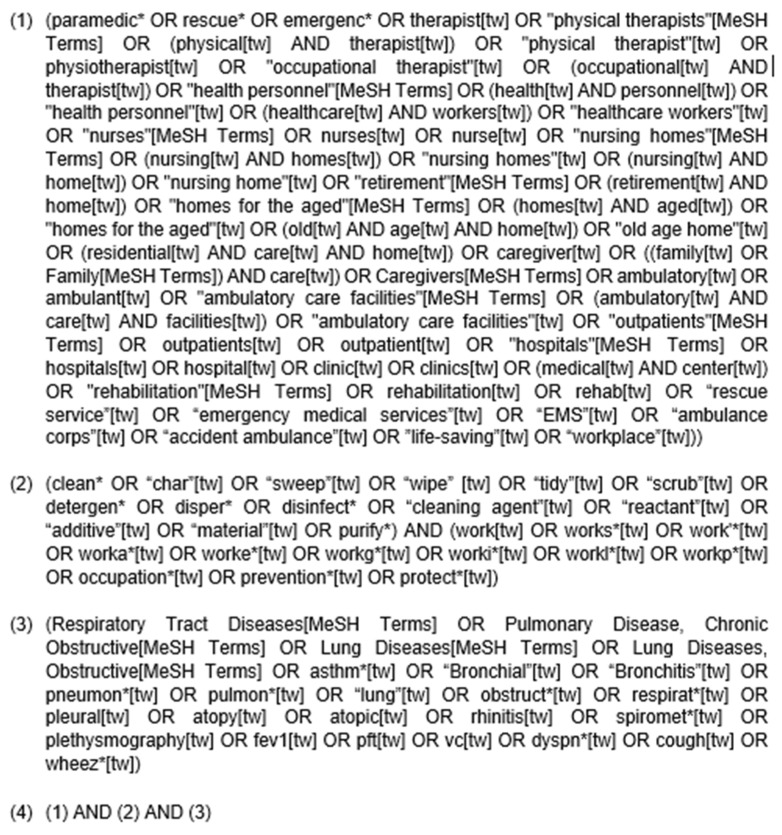
The MEDLINE (PubMed) search strategy.

We searched the electronic literature databases MEDLINE (Pubmed, United States National Library of Medicine, Bethesda, MD, USA) and Embase (Ovid, Wolters Kluwer N.V., Alphen aan den Rijn, The Netherlands) on 11 June 2019 and updated it on 4 February 2021. The MEDLINE search string is shown in Figure 1, and the Embase search string was then accordingly adapted. Additional studies were found manually by combing for relevant reviews using similar keywords that were used in our search strategy and by examining the references of the included studies. In addition, key papers were marked, and the citation tracking factor by Google Scholar was used to find additional relevant studies [18,19].

### 2.4. Study Selection and Data Collection

The search results were imported into an Endnote management system database, and the duplicate references were removed. Two independent scientists (K.R.S. and S.F. for the original search; S.F. and A.P. for the updated search) screened the titles and abstracts of the studies in order to exclude studies unrelated to the a priori defined research questions. Disagreements regarding the inclusion were discussed for a consensus decision. If disagreement was not resolved, the decision was made by a third reviewer (M.S.). The full texts of the remaining studies were screened by two independent reviewers (K.R.S. and S.F. for the original search; S.F. and A.P. for the updated search), and disagreements were discussed in meetings for consensus finding. The reasons for exclusion were recorded during the full-text review.

We extracted the following study characteristics:

- study design
- study region
- study population size
- population sampling information (time and type of recruitment, response, and follow-up)
- exposure and exposure assessment
- population characteristics of the exposure and comparison group (sex, age, duration of employment)
- outcome and outcome assessment
- prevalence or incidence of outcome
- relevant study results, including adjustment for confounders

The data extraction was done by one reviewer (K.R.S., S.F., or M.S.) and checked for accuracy by a second reviewer (K.R.S., S.F., or M.S.). Whenever there was missing or unclear information, we tried to obtain it through personal communication with the authors.

### 2.5. Risk of Bias Assessment

For each included study, two reviewers (K.R.S. and S.F.) assessed the risk of bias as high, low, or unclear against eight domains of bias, using a tool previously used for other occupational health reviews [20,21,22,23]. The hybrid tool uses SIGN (Scottish Intercollegiate Guidelines Network 2004) and CASP (Critical Appraisal Skills Program 2004/2006) assessment tools and contains several domains for risk of bias. Examples of reviews using this tool have been published previously [20,21,24], and the Risk of Bias tool can be found in the Supplementary Materials.

#### 2.5.1. Recruitment Procedure and Follow-Up

A low-risk study should have avoided selection bias by ensuring an adequate recruitment method, such as randomized sampling. The response for the study should be at least 50%, and if not, a non-participation analysis should have been performed. For cohort studies, if the loss to follow-up was below 20% and there was no substantial difference between the comparison groups, the risk of bias for this domain was rated as low. Similarly, for a case-control study to be rated as having a low risk of bias for this section, both cases and control subjects should have had a response of 50% or more, and if this number was not achieved, the substantial differential selection of cases and controls should have been excluded by a non-participation analysis.

#### 2.5.2. Exposure Definition and Measurement

If the exposure was accurately and objectively measured by the use of workplace observations, a validated questionnaire, or a questionnaire asking detailed questions on job characteristics, including tasks and exposures, then this domain was rated as having a low risk of bias. If comparing occupational groups (such as in the case of Type A studies), an adequate comparison nonexposed group should have been chosen. Exposures assessed with a job exposure matrix (JEM) were classified as having a high risk of bias because of the lack of accuracy.

#### 2.5.3. Outcome Source and Validation

To have a low risk of bias, the outcomes must have been objectively and accurately assessed in order to minimize bias. Examples of objective measurements include medical records and pulmonary function tests. If the outcomes were self-reported, this domain received a high risk of bias.

#### 2.5.4. Confounding

A study was considered to have low risk of bias in the confounding domain if the confounders age, sex, socioeconomic status (SES), and atopy were taken into account. Lower SES has been associated with a higher prevalence of asthma [25], while atopy may have an influence on the type of occupation a person chooses [26]. A directed acyclic graph (DAG) depicting the likely causal paths from exposure to cleaning and disinfection agents to obstructive respiratory diseases is shown in Figure S1 [27].

#### 2.5.5. Analysis Methods

If adequate statistical models were used to reduce bias and control for confounding, this domain was considered as having a low risk of bias.

#### 2.5.6. Chronology

If incident diseases were included or if a temporal relation could be established, this domain was considered low risk.

#### 2.5.7. Blinding of Assessors

If assessors were blinded, this domain was considered low risk.

#### 2.5.8. Funding

This was assessed in two areas: the sources of funding and the involvement of the funding body in the research. If a study was funded by non-profit organization(s) and it was not affected by sponsors, the domain was rated as having a low risk of bias. If the sponsoring organization participated in the data analysis or the study was probably affected by the sponsors, the domain was considered as having a high risk of bias.

#### 2.5.9. Conflicts of Interest

If the authors reported not having a conflict of interest, the domain was rated as having a low risk of bias. If at least one author had a conflict of interest, the domain was considered as having a high risk of bias.

#### 2.5.10. Overall Assessment of Risk of Bias

From the nine domains for risk of bias, we considered the domains described in Section 2.5.1, Section 2.5.2, Section 2.5.3, Section 2.5.4, Section 2.5.5 and Section 2.5.6 as major domains. Section 2.5.7, Section 2.5.8 and Section 2.5.9 were considered minor domains. In order for a study to have an overall low risk of bias, every major domain for risk of bias must have been rated as low risk. If one of the major domains was rated as having high or unclear risk, the study was judged to have a high overall risk of bias.

### 2.6. Statistical Analysis

Due to the heterogeneity of outcomes and exposures, it was decided to perform meta-analyses when at least two primary studies with similar exposures and outcomes were present. This procedure differed slightly from the PROSPERO protocol, which indicated a meta-analysis with at least three studies. Random effects models were used due to the heterogeneity of the studies. In general, we used the I^2^ value as a measure of heterogeneity but also considered that the I^2^ statistic is dependent on the size of the included studies [28,29]. The occurrence of possible publication biases was evaluated using funnel plots. Even though Cochrane recommends testing for funnel plot asymmetry when at least 10 studies are included [30], we decided to use funnel plots even with a lower study number for visualization, keeping in mind that no strong conclusions can be made. Odds ratios (ORs) overestimate the relative risk when the prevalence of the outcome of interest is high (>10%) [31]. Therefore, we converted ORs to prevalence ratios (PRs) using the formula by Zhang and Yu [31] when the prevalence of the outcome in the study population was greater than 10%. The statistical analysis was performed with Stata version 14 (StataCorp LLC, College Station, TX, USA) [32].

### 2.7. Quality of Evidence Assessment

To assess the quality of the total body of evidence, we used the Grading of Recommendations Assessment, Development, and Evaluation (GRADE) approach [33], following the example of Hulshof and colleagues [34], with some modifications [20,21,24]. We used three levels of quality: high, moderate, and low. An initial high level can only be achieved by having randomized studies. If only observational studies were included, the starting level would be “moderate”.

The quality of evidence was downgraded a level each for the following criteria:

1. study limitations (risk of bias)
2. indirectness
3. imprecision (range of the confidence intervals of studies greater than 2.0 relative risk)
4. publication bias

The quality of evidence was upgraded a level for each for the following criteria:

1. if the study findings had a large effect size (Relative Risk > 2.0)
2. if a positive dose-response relationship was found
3. if there is presence of residual confounding

We upgraded if “consideration of all plausible residual confounders, biases, or effect modification would underestimate the effect or suggest a spurious effect when results show no effect. If a study shows an association despite the presence of residual association, this would increase the confidence in the association [34]”.

We summed up the downgrading levels first, followed by the upgrading levels to obtain the final result.

## 3. Results

### 3.1. Study Selection

**Figure 2:**
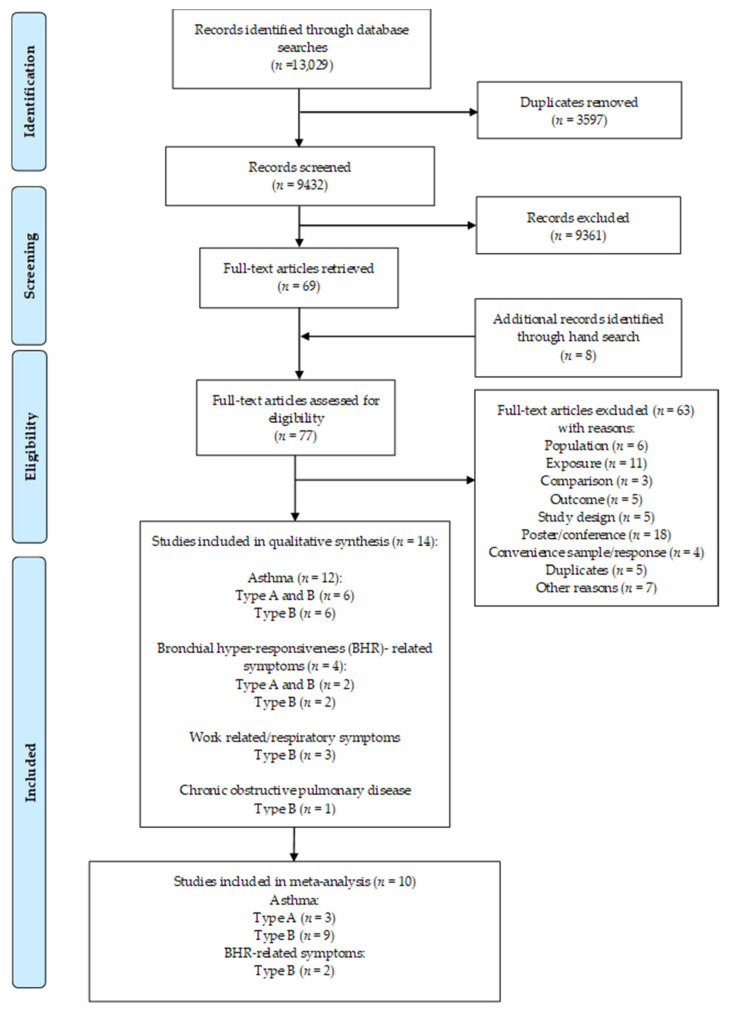
PRISMA Flowchart.

We found a total of 13,029 records in the electronic databases. There were 3597 duplicates removed, and 9432 records remained for the title and abstract screening. After excluding 9361 records during the title/abstract screening from further consideration and adding 8 studies identified through the hand search, 77 studies were assessed for eligibility. Our reasons for excluding studies are summarized in the PRISMA flow diagram (Figure 2), and a list of the studies excluded can be found in Table S1. The most common reasons for exclusion were irrelevant exposure (*n* = 11), population (*n* = 6), irrelevant outcome (*n* = 5), convenience sampling or low response (*n* = 4), study design (*n* = 5), and articles published as posters or conference papers (*n* = 18). The excluded articles with reasons for exclusion can be found in Table S2. After the full-text screening, we identified 14 articles. Six of those could be classified as both occupational and exposure-based (Type A and Type B studies) [35,36,37,38,39,40], and eight were classified as only exposure-based “Type B” studies [41,42,43,44,45,46,47,48]. Twelve studies investigated asthma [35,36,37,38,39,40,41,42,43,45,46,48], four studies investigated bronchial hyperresponsiveness (BHR)-related symptoms [36,38,41,48], and one study investigated COPD [47]. Three studies dealt with respiratory tract symptoms [42,43,44].

### 3.2. Asthma

We included twelve studies that considered asthma as the outcome [35,36,37,38,39,40,41,42,43,45,46,48], and four of these studies used the same population group [35,36,37,38]. Six studies [35,36,37,38,39,40] both compared nurses with an external comparison group and evaluated the risk of exposure to cleaning or disinfection tasks or chemicals (Types A and B), while six additional studies [41,42,43,45,46,48] only evaluated the exposure to cleaning/disinfection tasks or chemicals in nurses (Type B). The summary of the characteristics for the Type A and Type B studies is shown in Tables S3 and S4.

Studies included both males and females in the study population. With respect to study design, most studies used a cross-sectional design, with the exception of Mirabelli et al. [40], a prospective cohort in 13 European countries, Dumas et al. 2020 [45], and Dumas et al. 2021 [46], which used data from the Nurses’ Health Study 2 and 3, respectively. One study was performed in Canada [42], one study was from France [39], and nine studies were conducted in the United States of America (USA) [35,36,37,38,41,43,45,46,48].

Specifically, for “Type A”, most studies used only nurses as the exposed group with the exception of three studies [35,37,38] that included respiratory and occupational therapists in the exposed group. Only Gonzalez et al. [39] reported that the vast majority of nurses (>90%) were exposed to disinfectants, while about half of nurses were exposed to disinfectants or cleaning products in Mirabelli et al. [40]. Arif et al. [36] reported that most (88%) of the nurse practitioners in the studies worked in clinical settings, such as in hospitals, clinics, private practices, nursing homes, public schools, or in-home health, while 12% worked in non-clinical settings.

Regarding “Type B” studies, exposure to cleaning or disinfectant agents was mostly evaluated by self-report [35,37,39,40,41,42,43,46], while Gonzalez et al. [39] also included workplace observations for their exposure assessment. Three studies used a JEM to determine exposure based on the current [45,48] or longest-held jobs [36,37,38]. The exposure categories were heterogeneous: several studies focused on patient cleaning, instrument cleaning, surface cleaning, and sterilization of medical equipment [36,37,38,39,41], others also focused on specific agents, such as exposure to bleach, detergents, ammonia, glutaraldehyde, orthophthaldehyde, formalin, chloramines, and other agents [36,43,46,47,49], and another used general cleaning as the exposure [43]. One study focused on cleaning frequency [35], while another one focused on the duration of exposure to high-level disinfectants in years [46].

Several definitions were used for asthma. Six studies [36,38,39,40,41,42,45,46,48] used “new-onset asthma”, defined as either asthma that started after job entry or as asthma starting since the study baseline in the case of the prospective cohort studies. Four studies [37,39,41,43] used “current asthma” as an outcome, defined as either having had asthma in the previous 12 months [37,41], as ever having had asthma [39], or simply as having asthma at the present time [43]. Arif and Delclos [35] studied three outcomes: work-related asthma symptoms (symptoms that get better when away from work or worsen when returning to work, without history of physician-diagnosed asthma), work exacerbated asthma (symptoms that get better when away from work or worsen on return to work, with history of physician-diagnosed asthma), and occupational asthma (symptoms that get better when away from work or worsen on return to work, with history of physician-diagnosed asthma after beginning work as a healthcare professional). All studies, except for Ellett et al. [43], used self-reported physician-diagnosed asthma for their outcome assessment. The summary of results of Type A and Type B studies are shown in Tables S5 and S6 respectively.

#### 3.2.1. Risk of Bias

Type A (occupation-based) Studies.

All included studies were classified as having a high risk of bias (Table 2). The main reason for the high risk of bias was the exposure domain: either not all members of the exposed group (nurses) were exposed to cleaning or disinfectant agents [40] or the level of exposure was not provided [35,36,37,38,39]. Only Gonzalez et al. [39] reported that most nurses and auxiliary nurses were exposed to cleaning or disinfection tasks. However, the comparison group used by Gonzalez et al. [39] partly comprised charge nurses, physiotherapists, and midwives, and since these occupational groups might also be exposed, the exposure domain for the study was high risk. Other main reasons were that asthma was self-reported (rather than a documented physician diagnosis), main confounders were not considered (age, sex, SES, and atopy), and except for Mirabelli et al. [40], all analyses were unadjusted. Studies reporting “new-onset asthma”, meaning asthma occurring after entry into the profession, were considered to have a low risk of bias in the chronology domain.

Type B (exposure-based) Studies.

Likewise, none of the type B studies were rated as high quality. Several studies were rated as having a high risk of bias in the “recruitment and follow-up” domain due to low response or high loss to follow-up [40,41,43]. Studies that used JEM as their exposure assessment or a questionnaire with non-specific questions on exposures received a high risk in the “exposure” domain [36,37,38,41,43,48]. Dumas et al. 2020 [45] used a questionnaire on general cleaning and disinfection tasks (low risk) but used a JEM when evaluating specific disinfectants (high risk). Both risk of bias levels would then be considered when evaluating GRADE. Although the exposures were assessed with a previously developed questionnaire, the questionnaire used was reported to be only “moderate” with respect to the validity of assessment of cleaning exposures and was comparable to self-report [49]. Gonzalez et al. [39] and Caridi et al. [41] included 18–19% cleaners in the exposed group, and therefore those studies were also rated as having a high risk of bias in the “exposure domain”. All studies used self-reported asthma as outcomes, and therefore they were rated as having high risk in the “outcome” domain. No studies considered all the necessary confounders (age, sex, SES, and atopy), but most used adequate analysis methods, except for Ellett et al. 1996 [43] and Patel et al. 2020 [48], which reported unadjusted relative risks. Most studies also either used “new-onset asthma” to account for chronology or they were prospective in nature, and they were rated to have low risk of bias in that domain.

#### 3.2.2. Synthesis of Results

Type A Studies.

We used three studies for the meta-analysis, of which there are four results because we considered separate risks for nurses and auxiliary nurses in Gonzalez et al. 2014 [39]. We selected Delclos et al. 2007 [38] out of the four studies using the same population [35,36,37,38], first because Delclos et al. 2007, like Gonzalez et al. [39] and Mirabelli et al. [40], used self-reported asthma as the outcome, and we wanted to avoid outcome heterogeneity. Moreover, Delclos et al. 2007 [38] used physicians as the comparison group, which we deemed an appropriate comparison group. Arif et al. 2009 [36], on the other hand, used a combination of physicians and respiratory and occupational therapists, with the latter two occupational categories most likely also being exposed to cleaning or disinfectant agents.

**Figure 3:**
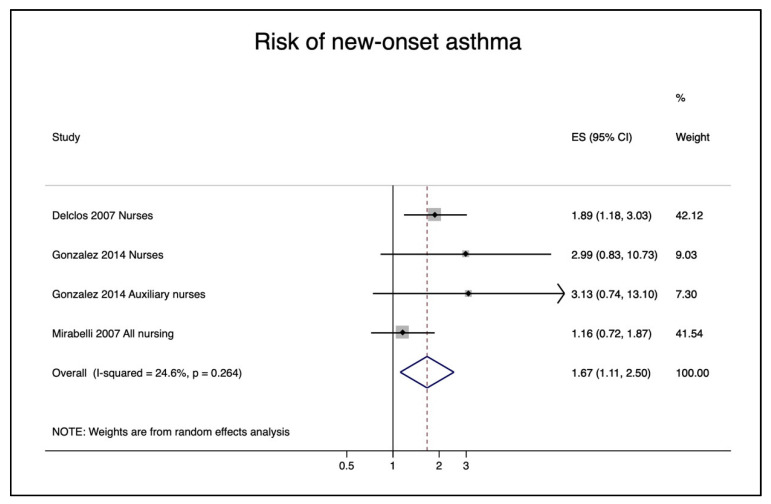
Pooled risks of new-onset asthma in nurses exposed to cleaning/disinfectant agents.

The pooled relative risk of nurses reporting a new onset of asthma (asthma after starting their professional careers) was 1.67 (95% CI 1.11–2.50) (Figure 3). The corresponding funnel plot shows no evidence of publication bias (Figure S2).

Type B Studies

For the Type B studies, we again had to choose between the four studies using the same study population [35,36,37,38]. This time though, we chose Arif et al. 2009 [36] for the meta-analysis, because it used “new-onset asthma” as the outcome, and unlike Delclos et al. 2007 [38], Arif et al. 2009 [36] adjusted for the important confounders age and sex (in addition to smoking, ethnicity, body mass index (BMI), and seniority). We included all other studies that used “new-onset asthma” as the outcome [39,40,41,48] or were prospective in nature [40,45,46] to increase between-study homogeneity.

**Figure 4:**
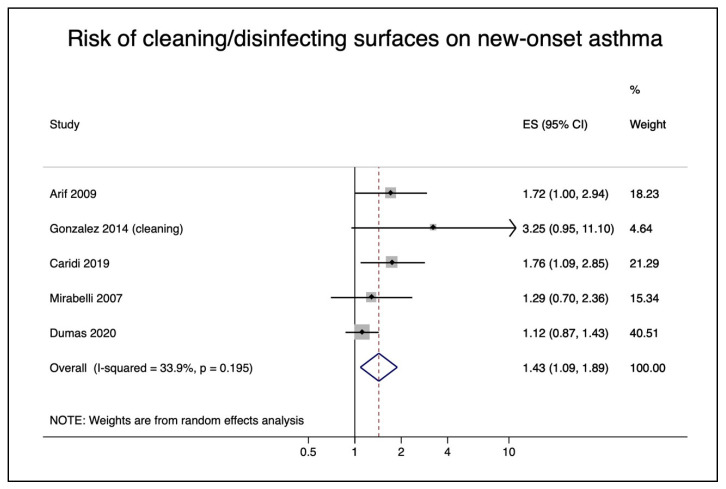
Pooled risk of cleaning/disinfecting surfaces on new-onset asthma in nurses.

In our first meta-analysis, we used the Gonzalez et al. “general cleaning” category for the pooled risk. This resulted in a pooled relative risk of exposure to cleaning or disinfecting surfaces in nurses of 1.43 (95% CI 1.09–1.89) (Figure 4). The corresponding funnel plot (Figure S3) did not indicate publication bias. In a sensitivity analysis, we used the Gonzalez et al. category of “general disinfection”, which yielded a similar relative risk 1.46 (95% CI 1.08–1.97) (Figure S4).

**Figure 5:**
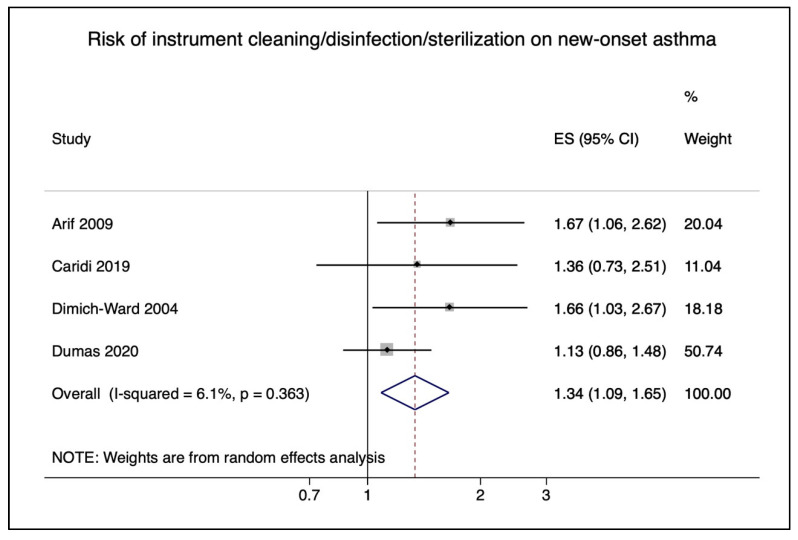
Pooled risk of cleaning/disinfecting/sterilization of instruments and medical equipment on new-onset asthma in nurses.

We used four studies to estimate the risk of nurses exposed to instrument cleaning/disinfection/sterilization [36,41,42,45]. The pooled RR was 1.59, 95% CI (1.19–2.13), shown in Figure 5. HLDs are used to disinfect medical devices chemically. We therefore included Dumas et al. 2021 [46], which investigated in a sensitivity analysis the association of exposure to HLDs on asthma. The pooled RR was similar with the addition of the study (RR = 1.35, 95% CI 1.14–1.59) (Figure S5).

Two studies were included to calculate the pooled risk of use of adhesives or solvents or chemicals in patient care [36,41]. The risk was raised but was not statistically significant (ES = 1.39; 95% CI: 0.90–2.16) (Figure S6).

**Figure 6:**
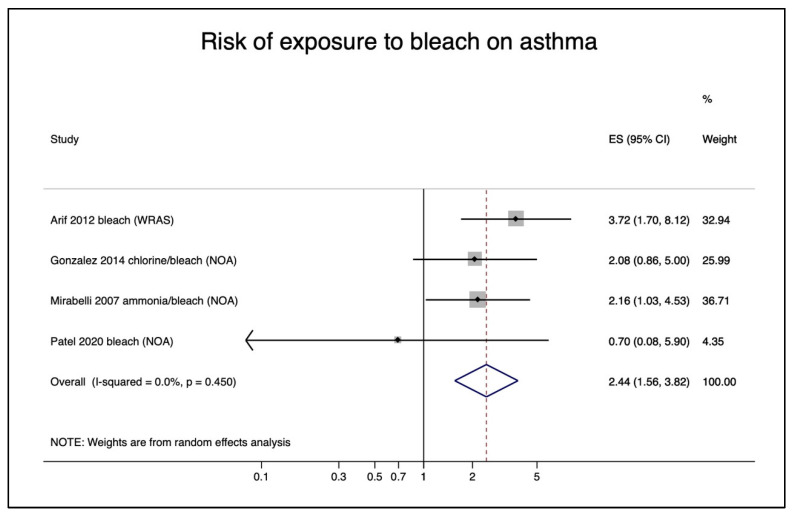
Risk of exposure to bleach on asthma (WRAS, work-related asthma symptoms, NOA, new-onset asthma).

For the pooled risks of exposure to specific chemicals, there were enough studies to perform a meta-analysis for bleach and glutaraldehyde exposure in nurses. This time we chose Arif et al. 2012 [35] out of the four studies [35,36,37,38], simply because it was the only study of the four that presented risks due to exposures to specific chemicals. However, Arif et al. 2012 [35] evaluated work-related asthma (WRAS), work-exacerbated asthma (WEA), and occupational asthma (OA) instead of new-onset asthma, so we chose to evaluate the pooled effect using these outcomes separately as sensitivity analyses. For exposure to bleach, the pooled relative risk was 2.44 (95% CI (1.56–3.82)) when choosing WRAS for Arif et al. 2012 [35] and new-onset asthma for Gonzalez et al. [39], Mirabelli et al. [40], and Patel et al. 2020 [48] (Figure 6). When using WEA for Arif et al. 2012 [35], the relative risk slightly decreased (RR = 2.18; 95% CI 1.34–3.55) and further decreased when using OA (RR = 2.08; 95 CI 1.24–3.49) (Figures S7 and S8). Nonetheless, all risk elevations were statistically significant. For the exposure of glutaraldehyde (GA) in nurses, we again used Arif et al. 2012 [35], Gonzalez et al. 2014 [39], Dumas et al. 2020 [45], and Patel et al. 2020 [48] with similar sensitivity analyses (Figures S9–S11). Again, the risks decreased when using WRAS (RR = 1.91; 95% CI 1.35–2.70), WEA (RR = 1.76; 95% CI 1.20–2.59), and OA 1.70 (RR = 1.14–2.53) as outcomes for Arif et al. 2012 [35]. The relative risk was statistically significant for all analyses regarding GA.

### 3.3. Bronchial Hyperresponsiveness (BHR)-Related Symptoms

We included four cross-sectional studies [36,37,41,48] investigating bronchial hyperresponsiveness (BHR)-related symptoms as an outcome. Two USA studies, which used the same study population, [36,38] were classified as both Type A (investigating the risk of nurses in comparison with a control group) and B (studying the risk of exposure to cleaning/disinfection tasks in nurses), while the other studies [41,48], also from the USA, were classified as Type B. The study characteristics and results for the Type A and B studies included are shown in Tables S7–S10.

#### 3.3.1. Risk of Bias

Type A Studies.

Both included studies used the same study population and both received a high overall risk of bias [36,38] (Table 3). Arif et al. 2009 [36] used a comparison group comprising physicians, respiratory therapists, occupational therapists, and “others”. Delclos et al. 2007 [38] used physicians as the comparison group, which we considered an adequate group, but neither study reported the amount cleaning or disinfection agents to which nurses were exposed. Both studies did not use a physician diagnosis for the outcome, did not consider confounders, and their analysis was univariable.

Type B Studies.

All included studies received a high overall risk of bias. Arif et al. 2009 [36], Delclos et al. 2007 [38], and Patel et al. 2020 [48] used a JEM as the exposure assessment and were therefore rated as having a high risk of bias in the exposure domain. Caridi et al. 2019 [41] investigated the risk of exposure to cleaning tasks in a mixed work group composed of nurses, cleaners, laboratory technicians, operating technicians, operating room technicians, and respiratory therapists, and hence the exposure domain was considered high risk. All four studies used a self-reported outcome, and no study considered the confounders of age, sex, or SES. Additionally, the response rate for Caridi et al. 2019 was low (13%), and therefore the recruitment procedure was also considered high risk.

#### 3.3.2. Synthesis of Results

No meta-analysis could be done for the bronchial hyperresponsiveness risk of nurses exposed to cleaning/disinfection agents compared with a reference population (Type A studies).

**Figure 7:**
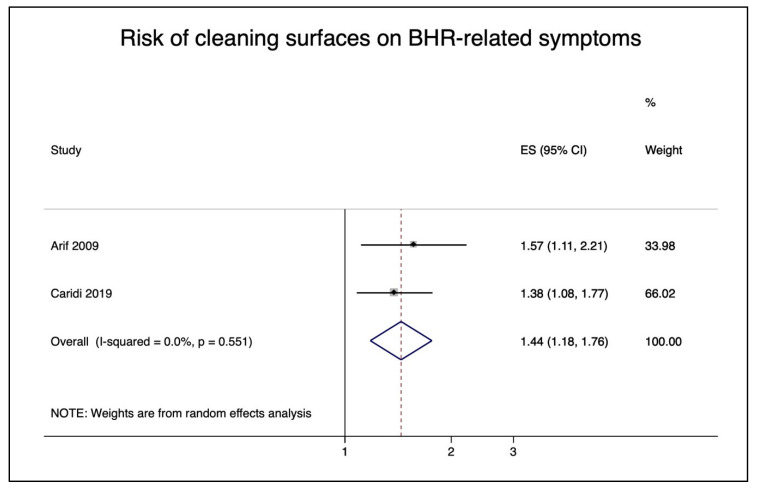
Pooled risk of cleaning surfaces on bronchial hyperresponsiveness (BHR)-related symptoms in nurses.

Arif et al. 2009 [36] and Delclos et al. 2007 [38], which used the same study population, were both similar in terms of exposure and outcome assessments. However, Arif et al. 2009 [36] was chosen for the Type B meta-analysis because it adjusted for age and sex. For Type B studies, the risk of exposure to cleaning surfaces on BHR-related symptoms in nurses was 1.44 (95% CI 1.18–1.76) (Figure 7). The risk of exposure to cleaning or sterilizing equipment on BHR-related symptoms was similar at 1.44 (95% CI 1.13–1.84) (Figure S12), and there was no elevated risk from using chemicals in patient care on BHR-related symptoms (ES = 1.02; 95% CI: 0.81–1.29) (Figure S13).

### 3.4. Work-Related and Respiratory Symptoms

There were three cross-sectional studies investigating the association of exposure to cleaning or sterilization on general respiratory symptoms [42,43,44] one originated from the United Kingdom (UK) [44], one from Canada [42], and one from USA [43]. Two studies evaluated exposure to glutaraldehyde [43,45]: Vyas et al. [45] evaluated nurses working in endoscopy units using questionnaires and site inspections as exposure assessments, while Dimich-Ward et al. [42] evaluated sterilization using glutaraldehyde on respiratory and physical therapists [42]. Ellett et al. [43] evaluated the general use of disinfectants on post-anesthesia nurses.

This category had diverse outcome definitions. For instance, Vyas et al. 2000 [44] evaluated work-related symptoms, meaning symptoms improving on rest days or symptoms experienced as more severe during a work shift, including chronic bronchitis, persistent cough, chest tightness, shortness of breath, and wheeze. The authors defined “lower respiratory tract symptoms” as having any of the above-mentioned symptoms. Dimich-Ward et al. [42] used “respiratory symptoms at any time in the last 12 months”, meaning asthma attack, wheeze, chest tightness, and other respiratory symptoms. We must note that the results for reported asthma (defined as self-reported physician-diagnosed asthma since entering the profession) were used in our section entitled “asthma” but were taken to also mean “respiratory symptoms”. Finally, Ellett et al. [43] used “respiratory problems” as their outcome, with no further definition. A summary of the characteristics and outcomes can be found in Tables S11 and S12.

All studies were evaluated as having high risk of bias due to the exposure and outcome assessments because not all confounders were considered and because a temporal relationship could not be established (Table S13).

### 3.5. Chronic Obstructive Pulmonary Disease (COPD)

Only one study investigating the association between occupational exposure to disinfectants among female nurses was included. Dumas et al. 2019 [47] used the Nurses’ Health Study II, a prospective cohort of USA registered nurses, for their study population. Tables S14 and S15 show the characteristics and results for the study. Nurses who had no history of COPD in 2009 were followed-up with questionnaires regarding their occupational exposures and frequency to disinfection, while exposure to specific disinfectants was evaluated using a JEM. The COPD outcome was assessed through self-reported physician-diagnosed COPD. The analysis controlled for age, smoking status, race, ethnicity, and body mass index but did not control for SES. The study was evaluated as having a high risk of bias (Table S16). The results of the study show a higher risk of developing COPD for nurses exposed to any disinfectants (HR= 1.35; 95% CI: 1.14–1.59). When a more stringent case definition for COPD is used, the risk is still increased, but it is not statistically significant. The authors also show a positive dose–response effect with the frequency of any disinfectant use (never, less than once a week, 1–3 times a week, 4–7 times a week) but most notably for cleaning surfaces. Exposure to formaldehyde, glutaraldehyde, hypochlorite bleach, hydrogen peroxide, alcohol, and QACs using a JEM were all associated with a higher risk of COPD, with statistically significant results for all mentioned except for formaldehyde.

### 3.6. Quality of Evidence Assessment

For the Type A studies (risk of new-onset asthma in nurses), the assessment of evidence resulted in an overall low quality of evidence for the risk of new-onset asthma on nurses (Table 4). We downgraded twice because all the evidence came from the high risk of bias studies and because we could not determine whether publication bias was present due to the low number of studies in the meta-analysis. We upgraded once because not all nurses in the study populations were exposed to cleaning or disinfection agents, most likely resulting in an underestimation of the risk. For the Type B studies (exposure to cleaning/disinfection agents), the assessment of evidence was also low, mostly because all studies were of high risk and because Gonzalez et al. 2014 and Caridi et al. 2019 contained cleaners in the exposure group, which resulted in the downgrade of the indirectness of evidence category. Furthermore, publication bias was unclear. In addition, for the risk of new-onset asthma by exposure to bleach, Mirabelli et al. 2007 combined the two heterogeneous categories “ammonia and/or bleach”. We upgraded for the large effect estimate category for new-onset asthma by exposure to bleach.

## 4. Discussion

To our knowledge, this is the first comprehensive systematic review and meta-analysis on the risk of cleaning and disinfection agents to healthcare workers. Our review found a 67% increased risk of new-onset asthma for nurses compared with the nonexposed comparison group and a 43% increased risk of new-onset asthma for nurses who cleaned or disinfected surfaces. Nurses who cleaned or disinfected instruments had a 34% increased risk of new-onset asthma. Nurses exposed to bleach or glutaraldehyde in the workplace had, respectively, 2.4- and 1.9-times increased risk of asthma than their nonexposed counterparts. Furthermore, nurses exposed to cleaning tasks had a 44% increased risk of BHR-related symptoms. The quality of evidence was low for the risks and exposures investigated.

### 4.1. Strengths and Limitations

Among the main strengths of this systematic review are the independent assessment by two reviewers of the title-abstract screening, the full-text screening, and the study quality. No restrictions in terms of publication language were made. Furthermore, a hand search complemented the systematic review to find additional relevant studies. The study design was published a priori on PROSPERO. Our review applied the latest recommendations on the conduct of systematic reviews of human observational studies, including the assessment of the methodological quality of the included studies [50].

The certainty of the results of the present systematic review is limited by the mainly low methodological quality of the included studies used to answer our research question. None of the included studies had an overall low risk of bias, and a sensitivity analysis studying only high-quality studies could not be undertaken.

A major reason for the high risk of bias assessment was the exposure assessment. An ideal study would have compared only nurses who were exposed to cleaning or disinfection agents with another nonexposed occupational group in which an average risk of obstructive respiratory disease could be assumed. In most of the studies, either groups of nurses or healthcare workers (both exposed and nonexposed) were compared with another occupational group (Type A studies) or in some studies a mix of occupational groups (some including nurses and cleaners) exposed to cleaning/disinfection agents were compared with the same occupational (but nonexposed) group (Type B). In neither of these two cases can an unbiased effect of nurses exposed to disinfection/cleaning agents be calculated—information bias can lead to an underestimate (as in the first comparison) or an under- or overestimate (as in the second comparison) of the actual risk. The exposed population in Gonzalez et al. [39] and Caridi et al. [41] comprised not only nurses: 17–18% were professional cleaners. Cleaners may be more likely to be exposed to cleaning and disinfectant agents than nurses and may therefore have a higher risk for asthma. Therefore, for these two studies, the nurses’ risk for asthma might be an overestimate of the actual risk. In addition, exposures were evaluated using JEMs or self-reports. A more accurate assessment would include workplace observations, as was done by Gonzalez et al. [39].

The outcome assessment was mainly done by self-report via questionnaire. A more objective assessment would include assessment through exams (via spirometry, body plethysmography, or changes in the objective markers such as forced expiratory volume in one second (FEV1)) or through hospital or medical records.

In order to investigate the total effect between occupational exposure to disinfectants and chronic respiratory diseases such as asthma, studies must adjust for age, sex, atopy, and socio-economic status (SES). Only a few studies adjusted for atopy, but no studies adjusted for SES. The lack of adjustment for SES may lead to an overestimation of the risk. Nonetheless, several studies did adjust for smoking, BMI, the use of latex gloves, and ethnicity. Smoking, BMI, and ethnicity could indeed be confounders via the SES pathway (see Figure S1). However, they are as well in the causal pathway between the exposure and outcome, which could result in an underestimation of the risk. First, we take BMI as an example, also depicted in the DAG in Figure S1. Although indeed BMI can be a confounder via the SES causal pathway, the nursing occupation, involving frequent movement, will be associated with lower BMI. In turn BMI influences the development of asthma [51], indicating that BMI is an intermediary factor. Similarly, work-related stress caused by the profession can influence BMI. Similarly, smoking may be a confounder via the SES or age pathway. However, smoking is also an intermediary factor. Some jobs associated with higher stress, such as nursing, may lead to increased smoking, which in turn will increase the risk of obstructive respiratory diseases. The use of latex gloves is also in the causal pathway, as the occupation is associated with a higher use of latex gloves. In turn, latex gloves will also cause asthma. Such causal mechanisms should be looked at in detail while deciding which variables to adjust for in the analysis, as they may lead to under- or overestimation of the risks.

A major limitation was the potential for a healthy worker effect, which would underestimate the effect size. It has been shown that workplace-based asthma studies are particularly prone to healthy worker bias [26]. One study has shown that nurses with a history of asthma were more likely to move to jobs with lower exposure to disinfectants [52]. Four [40,45,46,47] out of fourteen studies in this review were prospective in nature, while the others were cross-sectional studies especially affected by such biases. Nevertheless, even prospective studies might suffer from healthy worker biases. In a sensitivity analysis using age as a proxy for duration of occupation, Dumas et al. 2021 [46] stratified by the nurses’ age at baseline and found that younger nurses exposed to HLDs had a higher risk of incident asthma than older nurses exposed to HLDs (<34 years: HR = 1.75; 95% CI 1.03–2.98; ≥34 years: HR = 1.26; 95% CI 0.90–1.78). Future studies should consider design and such analyses to correct or depict the likelihood of a healthy worker effect. Prospective studies investigating healthcare workers at the start of their careers should be able minimize healthy worker effects.

The included studies encompassed mainly nurses, respiratory therapists, and physiotherapists. There were no studies investigating the risk of asthma on other groups, such as rescue service personnel or on nursing home workers, although this occupational group may be equally or more exposed to cleaning and disinfection agents than other healthcare personnel.

### 4.2. Agreement with Other Studies and Reviews

This review is in agreement with other reviews that have found an increased risk of workers (mostly professional cleaners) exposed to cleaning and disinfecting agents [11,12,13,14]. It is known that some common agents used for cleaning and disinfecting are responsible for occupational asthma, such as chloramine-T, formaldehyde, glutaraldehyde (GA), quaternary ammonium compounds (QACs), and bleach [8,53]. Indeed, in our review, the highest risk for asthma was seen for nurses exposed to bleach and GA. Although a meta-analysis could not be made for QACs because of the lack of studies specifying this agent, Gonzalez et al. [39] reported a more than 6-fold risk of new-onset asthma for nurses exposed to those cleaning agents.

### 4.3. Implications for Practice

Germany recognizes obstructive respiratory diseases caused either by allergenic or chemical-irritative/toxic substances as occupational diseases. In 2016, the Institution for Statutory Social Accident Insurance and Prevention in the Health Care and Welfare Services (BGW) in Germany received 381 reports of obstructive respiratory diseases, indicating a suspicion of disease due to the occupation. Only about 8% of the reports corresponded to workers in hospitals and clinics [54]. In the year 2019, 58 reports of a suspected indication of obstructive airway disease due to allergenic or toxic substances in healthcare workers working in hospitals, clinics, inpatient geriatric care, or outpatient services were received by the BGW (M. Dulon, personal communication) [55].

In the decade spanning the years 2010–2019, the German Social Accident Insurance (DGUV) confirmed a total of 120 cases of obstructive respiratory diseases due to allergenic or toxic substances as occupational diseases in healthcare workers (including nurses, midwives, and rescue service personnel). Almost half of those cases (47%) were determined to have been caused by disinfection or cleaning agents [56].

A recent review found that patients, employers, and healthcare professionals lack awareness and underreport work-related asthma [57]. Therefore, it is possible that the above numbers do not reflect the current burden of obstructive respiratory diseases in healthcare personnel. Primary care physicians should take detailed workplace histories of their patients, along with improving asthma diagnosis. The above review found that patients may not be aware that exposure to cleaning agents may result in asthma, and about half of the workers thought that the asthma symptoms they were experiencing were normal [57]. Because of fear of stigma, even if workers are aware of the association between agents and asthma, they still may underreport their symptoms for fear of losing their job or may be forced to change their job. Importantly, employers should be sensitized to the link between cleaning and disinfection tasks and asthma. Alternative cleaning products that are safer for the respiratory system should be made available by manufacturers and employers.

## 5. Conclusions

In this systematic review, evidence of an increased risk of asthma in nurses exposed to cleaning or disinfection agents was found. Although the overall evidence was rated as low, the limitations found in this review hint at a potential underestimation of the real risk. These findings highlight the obligation for prevention practices. There is a need to sensitize healthcare workers (as patients), as well as primary care physicians and employers. Alternative cleaning products that are safer for the respiratory system should be made available to nurses.

## Figures and Tables

**Table 1:** Study eligibility criteria according to Population, Exposure, Comparison, Outcome, and Study Design.

Category	Inclusion	Exclusion
Population (P)	Employable population of both sexes, between 16 and 70 years old	Non-employable population (for instance, with disabilities or illnesses that render them not being able to work, adolescents and children (under 16 years of age), and elderly people (over 70 years of age)
Exposure (E)	Type A: Employment as a nurse or in rescue service with an occupational exposure to cleaning or disinfection agents Type B: Exposure to cleaning or disinfection agents, in which nurses or rescue workers were an important part of the exposed population	Type A: Employment in settings with no occupational exposure to cleaning or disinfection agents Type B: Exposure to agents other than cleaning or disinfection
Control/Comparator (C)	Type A: Population employed in other occupational groups where an average risk can be presumed (external comparison group) Type B: Working population not exposed to cleaning or disinfection agents (internal or external comparison group)	Type A and B: Groups with an elevated risk for respiratory diseases, due to exposure to inorganic/organic dusts, chemical irritants, and allergenic substances
Outcome (O)	Obstructive diseases such as COPD and asthma: medical diagnosis such as in doctors’ records, hospital records, health insurance records; self-report Lung function abnormalities: changes in the objective markers FEV1, PEF, VC, and other outcomes of pulmonary function tests (spirometry, body plethysmography)	Rhinitis, skin allergies, and other non-respiratory, non-obstructive diseases
Study design (S)	Cohort, case-control, cross-sectional	Qualitative studies, studies with only abstracts, conference papers/posters, reviews, letters, editorials; studies using a convenience sample, not reporting response, or with a response less than 10%

COPD, chronic obstructive pulmonary disease; FEV1, forced expiratory volume in 1 s; PEF, peak expiratory flow; VC, vital capacity.

**Table 2:** Risk of bias for asthma studies.

Study ID	Major Domains						Minor Domains			Overall
	Recruitment Procedure and Follow-Up	Exposure Definition and Measurement	Outcome Source and Validation	Confounding	Analysis Method	Chronology	Assessor Blinding	Funding	Conflict of Interest	
Type A (Risk in nurses exposed to cleaning/disinfection agents)										
Arif et al. 2009 * [36]	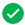	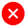	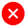	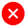	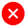	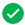	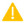	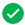	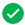	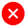
Delclos et al. 2007 * [38]	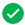	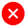	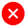	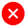	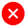	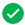	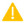	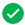	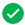	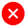
Delclos et al. 2009 * [37]	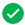	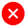	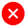	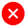	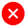	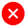	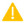	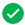	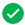	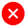
Arif and Delclos 2012 * [35]	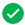	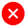	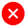	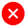	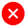	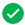	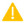	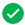	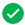	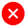
Gonzalez et al. 2014 [39]	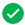	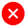	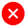	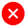	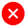	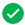	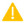	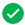	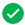	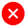
Mirabelli et al. 2007 [40]	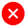	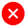	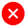	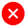	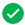	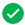	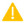	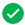	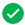	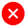
Type B (Risk of exposure to cleaning and disinfectants in nurses)										
Arif et al. 2009 * [36]	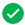	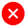	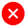	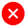	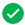	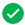	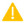	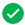	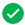	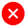
Delclos et al. 2007 * [38]	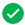	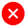	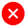	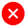	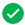	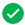	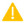	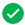	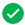	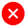
Delclos et al. 2009 * [37]	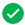	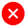	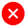	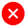	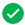	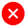	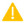	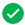	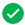	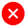
Arif and Delclos 2012 * [35]	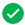	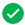	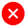	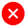	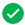	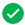	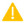	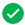	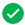	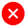
Gonzalez et al. 2014 [39]	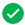	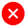	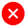	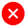	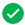	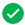	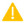	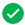	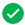	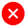
Mirabelli et al. 2007 [40]	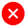	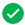	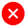	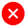	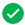	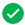	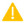	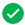	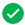	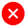
Caridi et al. 2019 [41]	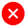	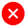	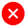	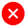	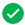	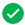	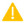	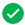	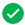	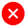
Dimich-Ward et al. 2004 [42]	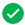	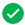	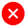	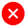	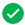	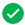	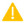	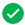	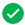	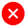
Ellett et al. 1996 [43]	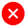	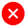	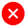	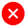	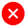	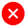	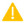	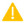	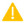	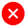
Dumas et al. 2021 [46]	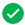	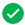	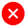	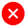	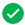	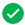	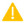	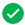	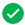	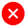
Patel et al. 2020 [48]	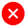	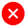	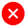	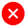	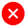	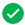	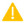	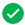	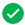	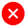
Dumas et al. 2020 † [45]	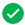	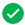 / 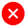	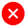	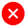	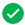	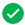	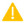	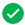	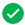	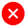

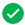
 Low Risk; 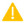 Unclear; 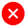 High risk; * Same study population; † Exposure low risk for “disinfection tasks” /high risk for “specific disinfectants”.

**Table 3:** Risk of bias for bronchial hyperresponsiveness (BHR)-related symptoms.

Study ID	Major Domains						Minor Domains			Overall
	Recruitment Procedure and Follow-Up	Exposure Definition and Measurement	Outcome Source and Validation	Confounding	Analysis Method	Chronology	Assessor Blinding	Funding	Conflict of Interest	
Type A (Risk in nurses exposed to cleaning/disinfection agents)										
Arif et al. 2009 * [36]	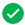	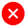	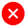	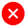	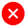	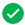	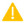	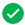	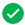	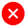
Delclos et al. 2007 * [38]	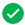	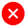	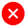	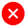	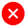	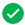	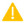	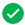	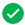	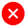
Type B (Risk of exposure to cleaning and disinfectants in nurses)										
Arif et al. 2009 * [36]	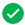	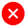	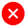	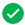	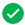	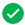	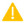	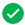	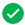	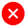
Delclos et al. 2007 * [38]	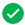	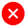	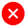	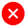	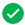	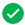	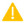	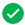	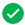	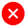
Caridi et al. 2019 [41]	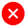	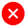	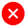	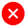	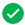	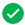	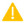	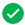	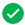	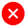
Patel et al. 2020 [48]	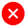	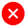	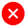	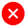	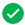	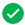	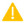	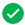	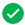	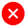

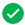
 Low Risk; 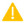 Unclear; 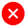 High risk; * Same study population.

**Table 4:** Assessment of evidence for the risk of studied outcomes based on Grading of Recommendations, Assessment, Development, and Evaluation (GRADE) framework.

Risk	Quality of Study Limitations: ↓	Indirectness of Evidence: ↓	Inconsistency: ↓	Imprecision, Range Confidence Interval Effect Size > 2.0: ↓	Publication Bias, Yes or Unclear: ↓	Effect Estimate >2.0: ↑ >5.0: ↑↑	Dose–Response Effect: ↑	Residual Confounding: ↑	Overall Certainty (High, Moderate, Low)
New-onset asthma in nurses	yes ↓ ^1^	no (-)	no (-)	no (-)	unclear ↓	no (-)	no (-)	yes ↑ ^2^	low
New-onset asthma by cleaning/disinfecting surfaces in nurses	yes ↓ ^1^	yes ↓ ^3^	no (-)	no (-)	unclear ↓	no (-)	no (-)	no (-)	low
New-onset asthma by instrument cleaning/disinfecting/sterilization	yes ↓ ^1^	yes ↓ ^4^	no (-)	no (-)	unclear ↓	no (-)	no (-)	no (-)	low
New-onset asthma by exposure to bleach	yes ↓ ^1^	yes ↓ ^5^	no (-)	yes (-)↓ ^6^	unclear ↓	yes ↑ ^7^	no (-)	no (-)	low
New-onset asthma by exposure to glutaraldehyde	yes ↓ ^1^	no (-)	no (-)	no (-)	unclear ↓	no (-)	no (-)	no (-)	low
Bronchial hyperresponsiveness (BHR)-related symptoms by cleaning surfaces	yes ↓ ^1^	yes ↓ ^8^	no (-)	no (-)	unclear ↓	no (-)	no (-)	no (-)	low
BHR-related symptoms by instrument cleaning/disinfecting/sterilization	yes ↓ ^1^	yes ↓ ^8^	no (-)	no (-)	unclear ↓	no (-)	no (-)	no (-)	low

^1^ All the evidence comes from high risk of bias studies. ^2^ Not all nurses in the study population were exposed to cleaning/disinfecting agents. As the study included exposed and nonexposed nurses, this would likely result in an underestimation of the risk. ^3^ The study population was exposed to cleaners for two studies (Gonzalez et al. 2014 and Caridi et al. 2019), which most likely will result in an overestimation of the risk. ^4^ The study population was exposed to cleaners (18%) for one study (Caridi et al. 2019), which may result in an overestimation of the risk. ^5^ The study population was exposed to cleaners in Gonzalez et al. 2014, which most likely will result in an overestimation of the risk; Mirabelli et al. 2007 combines the two heterogeneous categories “ammonia and/or bleach”. ^6^ Pooled confidence interval range >2 (1.56–3.82). ^7^ The pooled effect estimate was 2.44 (greater than 2.0 but less than 5.0); 95% CI (1.56–3.82). ^8^ The study population was exposed to cleaners in Caridi et al. 2019, which most likely will result in an overestimation of the risk.

## Supplementary Materials

The following are available online at https://www.mdpi.com/article/10.3390/ijerph18105159/s1, Figure S1: Directed acyclic graph of the causal model between healthcare worker’s exposure to cleaning/disinfectant agents (E) and chronic respiratory diseases (O), Figure S2: Funnel plot of studies investigating the risk of new-onset asthma in nurses, Figure S3: Funnel plot of studies investigating the risk of new-onset asthma in nurses, Figure S4: Forest plot of studies investigating the risk of cleaning/disinfecting surfaces for new-onset asthma, Figure S5: Forest plot of studies investigating the risk of instrument cleaning/disinfection/sterilization for new-onset asthma, Figure S6: Forest plot of studies investigating the risk of use of adhesive/solvents/chemicals in patient care on new-onset asthma, Figure S7: Forest plot of studies investigating the risk of exposure to bleach on asthma, Figure S8: Forest plot of studies investigating the risk of exposure to bleach on asthma, Figure S9: Forest plot of studies investigating the risk of exposure to glutaraldehyde on asthma, Figure S10: Forest plot of studies investigating the risk of exposure to glutaraldehyde on asthma, Figure S11: Forest plot of studies investigating the risk of exposure to glutaraldehyde on asthma, Figure S12: Forest plot of studies investigating the risk of cleaning/sterilizing equipment on bronchial hyperresponsiveness (BHR)- related symptoms, Figure S13: Forest plot of studies investigating the risk of use of adhesive/solvents/chemicals in patient care on bronchial hyperresponsiveness (BHR)- related symptoms, Table S1: Excluded studies and reasons for exclusion, Table S2: Risk of bias instrument, Table S3: Characteristics of included studies investigating asthma (Type A), Table S4: Characteristics of included studies investigating risk of cleaning-related tasks on asthma (Type B Studies), Table S5: Results for included studies on asthma in nurses exposed to cleaning/disinfection agents (Type A), Table S6: Results for included studies on risk of exposure to cleaning/disinfection agents on asthma in nurses (Type B), Table S7: Characteristics of included studies on bronchial hyper-responsiveness (BHR)-related symptoms (Type A), Table S8: Characteristics of included studies investigating risk of cleaning-related tasks on bronchial hyper-responsiveness (BHR)-related symptoms (Type B), Table S9: Results for included studies on bronchial hyper-responsiveness (BHR)-related symptoms (Type A), Table S10: Results for included studies on bronchial hyper-responsiveness (BHR)-related symptoms (Type B), Table S11: Characteristics of included studies investigating risk of work-related and respiratory symptoms (Type B), Table S12: Results of studies with work-related symptoms and respiratory symptoms (Type B), Table S13: Risk of bias of included studies investigating work-related symptoms and respiratory symptoms, Table S14: Characteristics of included studies investigating risk of chronic obstructive pulmonary disease (COPD) (Type B), Table S15: Results of included studies investigating risk of chronic obstructive pulmonary disease (COPD) (Type B), Table S16: Risk of bias of study included investigating chronic obstructive pulmonary disease (COPD).

## References

[1] Arbeit B.F., Statistisches Bundesamt (2020). Beschäftigtenstatistik.

[2] (2020). Statistik der Bundesagentur für Arbeit. Tabellen, Beschäftigte Nach Berufen (KldB 2010) (Quartalszahlen). http://statistik.arbeitsagentur.de.

[3] Eurostat (2020). Number of Nurses and Midwives on the Rise.

[4] Plecher H. Total Population of the European Union (EU) 2020. https://www.statista.com/statistics/253372/total-population-of-the-european-union-eu/.

[5] Saito R., Virji M.A., Henneberger P.K., Humann M.J., LeBouf R.F., Stanton M.L., Liang X., Stefaniak A.B. (2015). Characterization of cleaning and disinfecting tasks and product use among hospital occupations. Am. J. Ind. Med..

[6] Dumas O., Donnay C., Heederik D.J.J., Héry M., Choudat D., Kauffmann F., Le Moual N. (2012). Occupational exposure to cleaning products and asthma in hospital workers. Occup. Environ. Med..

[7] Widders G.S.A., Seewald M., Poldrack R., Bergen P., Hofmann A., Kohlstock C., Schicht B., Spengler A. (2011). Rahmenhygieneplan für Rettungs-und Krankentransportdienste. https://www.gesunde.sachsen.de/download/Download_Gesundheit/RHPL_Rettungsdienst.pdf.

[8] Chan-Yeung M., Malo J.-L. (1995). Occupational asthma. N. Engl. J. Med..

[9] Quirce S., Barranco P. (2010). Cleaning agents and asthma. J. Investig. Allergol. Clin. Immunol..

[10] Folletti I., Siracusa A., Paolocci G. (2017). Update on asthma and cleaning agents. Curr. Opin. Allergy Clin. Immunol..

[11] Jaakkola J.J., Jaakkola M.S. (2006). Professional cleaning and asthma. Curr. Opin. Allergy Clin. Immunol..

[12] Folletti I., Zock J.-P., Moscato G., Siracusa A. (2014). Asthma and rhinitis in cleaning workers: A systematic review of epidemiological studies. J. Asthma.

[13] Siracusa A., De Blay F., Folletti I., Moscato G., Olivieri M., Quirce S., Raulf-Heimsoth M., Sastre J., Tarlo S.M., Walusiak-Skorupa J. (2013). Asthma and exposure to cleaning products-a European academy of allergy and clinical immunology task force consensus statement. Allergy.

[14] Vincent M.J., Parker A., Maier A. (2017). Cleaning and asthma: A systematic review and approach for effective safety assessment. Regul. Toxicol. Pharmacol..

[15] Stroup D.F., Berlin J.A., Morton S.C., Olkin I., Williamson G.D., Rennie D., Moher D., Becker B.J., Sipe T.A., Thacker S.B. (2000). Meta-analysis of observational studies in epidemiology: A proposal for reporting. Meta-analysis of Observational Studies in Epidemiology (MOOSE) group. JAMA.

[16] Moher D., Liberati A., Tetzlaff J., Altman D.G., The P.G. (2009). Preferred Reporting items for systematic reviews and meta-analyses: The prisma statement. PLoS Med..

[17] Higgins J.G.S. (2011). Cochrane Handbook for Systematic Reviews of Interventions.

[18] Bakkalbasi N., Bauer K., Glover J., Wang L. (2006). Three options for citation tracking: Google Scholar, Scopus and Web of Science. Biomed. Digit. Libr..

[19] Giles J. (2005). Start Your Engines. Nature.

[20] Romero Starke K., Kofahl M., Freiberg A., Schubert M., Groß M.L., Schmauder S., Hegewald J., Kämpf D., Stranzinger J., Nienhaus A. (2020). The risk of cytomegalovirus infection in daycare workers: A systematic review and meta-analysis. Int. Arch. Occup. Environ. Health.

[21] Romero Starke K., Kofahl M., Freiberg A., Schubert M., Groß M.L., Schmauder S., Hegewald J., Kämpf D., Stranzinger J., Nienhaus A. (2019). Are daycare workers at a higher risk of parvovirus B19 infection? A systematic review and meta-analysis. Int. J. Environ. Res. Public Health.

[22] Ijaz S., Verbeek J., Seidler A., Lindbohm M.L., Ojajärvi A., Orsini N., Costa G., Neuvonen K. (2013). Night-shift work and breast cancer--a systematic review and meta-analysis. Scand. J. Work Environ. Health.

[23] Shamliyan T.A., Kane R.L., Ansari M.T., Raman G., Berkman N.D., Grant M., Janes G., Maglione M., Moher D., Nasser M. (2011). Development quality criteria to evaluate nontherapeutic studies of incidence, prevalence, or risk factors of chronic diseases: Pilot study of new checklists. J. Clin. Epidemiol..

[24] Bolm-Audorff U., Hegewald J., Pretzsch A., Freiberg A., Nienhaus A., Seidler A. (2020). Occupational noise and hypertension risk: A systematic review and meta-analysis. Int. J. Environ. Res. Public Health.

[25] Uphoff E., Cabieses B., Pinart M., Valdés M., Antó J.M., Wright J. (2015). A systematic review of socioeconomic position in relation to asthma and allergic diseases. Eur. Respir. J..

[26] Le Moual N., Kauffmann F., Eisen E.A., Kennedy S.M. (2008). The healthy worker effect in asthma: Work may cause asthma, but asthma may also influence work. Am. J. Respir. Crit. Care Med..

[27] Textor J., van der Zander B., Gilthorpe M.S., Liśkiewicz M., Ellison G.T. (2017). Robust causal inference using directed acyclic graphs: The R package ‘dagitty’. Int. J. Epidemiol..

[28] Borenstein M., Hedges L.V., Higgins J.P., Rothstein H.R. (2011). Introduction to Meta-Analysis.

[29] Rücker G., Schwarzer G., Carpenter J.R., Schumacher M. (2008). Undue reliance on I 2 in assessing heterogeneity may mislead. BMC Med. Res. Methodol..

[30] Higgins J.P.T., Thomas J., Chandler J., Cumpston M., Li T., Page M.J., Welch V.A. (2021). Cochrane Handbook for Systematic Reviews of Interventions.

[31] Zhang J., Yu K.F. (1998). What’s the relative risk?A Method of correcting the odds ratio in cohort studies of common outcomes. JAMA.

[32] StataCorp (2019). Stata Statistical Software: Release 14.

[33] Guyatt G., Oxman A.D., Akl E.A., Kunz R., Vist G., Brozek J., Norris S., Falck-Ytter Y., Glasziou P., Debeer H. (2011). GRADE guidelines: 1. Introduction—GRADE evidence profiles and summary of findings tables. J. Clin. Epidemiol..

[34] Hulshof C.T., Colosio C., Daams J.G., Ivanov I.D., Prakash K., Kuijer P.P., Leppink N., Mandic-Rajcevic S., Masci F., van der Molen H.F. (2019). WHO/ILO work-related burden of disease and injury: Protocol for systematic reviews of exposure to occupational ergonomic risk factors and of the effect of exposure to occupational ergonomic risk factors on osteoarthritis of hip or knee and selected other musculoskeletal diseases. Environ. Int..

[35] Arif A.A., Delclos G.L. (2012). Association between cleaning-related chemicals and work-related asthma and asthma symptoms among healthcare professionals. Occup. Environ. Med..

[36] Arif A.A., Delclos G.L., Serra C. (2009). Occupational exposures and asthma among nursing professionals. Occup. Environ. Med..

[37] Delclos G.L., Gimeno D., Arif A.A., Benavides F.G., Zock J.-P. (2009). Occupational exposures and asthma in health-care workers: Comparison of self-reports with a workplace-specific job exposure matrix. Am. J. Epidemiol..

[38] Delclos G.L., Gimeno D., Arif A.A., Burau K.D., Carson A., Lusk C., Stock T., Symanski E., Whitehead L.W., Zock J.-P. (2007). Occupational risk factors and asthma among health care professionals. Am. J. Respir. Crit. Care Med..

[39] Gonzalez M., Jégu J., Kopferschmitt M.C., Donnay C., Hedelin G., Matzinger F., Velten M., Guilloux L., Cantineau A., de Blay F. (2014). Asthma among workers in healthcare settings: Role of disinfection with quaternary ammonium compounds. Clin. Exp. Allergy.

[40] Mirabelli M.C., Zock J.-P., Plana E., Antó J.M., Benke G., Blanc P.D., Dahlman-Höglund A., Jarvis D.L., Kromhout H., Lillienberg L. (2007). Occupational risk factors for asthma among nurses and related healthcare professionals in an international study. Occup. Environ. Med..

[41] Caridi M.N., Humann M.J., Liang X., Su F.-C., Stefaniak A.B., LeBouf R.F., Stanton M.L., Virji M.A., Henneberger P.K. (2019). Occupation and task as risk factors for asthma-related outcomes among healthcare workers in New York City. Int. J. Hyg. Environ. Health.

[42] Dimich-Ward H., Wymer M.L., Chan-Yeung M. (2004). Respiratory health survey of respiratory therapists. Chest.

[43] Ellett M.L., Fullhart J.W., Wright K.B. (1996). Society of Gastroenterology Nurses and Associates, Inc.(SGNA) Endoscopic Disinfectant Survey results compared with control group. Gastroenterol. Nurs..

[44] Vyas A., Pickering C., Oldham L., Francis H., Fletcher A., Merrett T., Niven R.M. (2000). Survey of symptoms, respiratory function, and immunology and their relation to glutaraldehyde and other occupational exposures among endoscopy nursing staff. Occup. Environ. Med..

[45] Dumas O., Boggs K.M., Quinot C., Varraso R., Zock J.P., Henneberger P.K., Speizer F.E., Le Moual N., Camargo C.A. (2020). Occupational exposure to disinfectants and asthma incidence in U.S. nurses: A prospective cohort study. Am. J. Ind. Med..

[46] Dumas O., Gaskins A.J., Boggs K.M., Henn S.A., Le Moual N., Varraso R., Chavarro J.E., Camargo C.A. (2021). Occupational use of high-level disinfectants and asthma incidence in early- to mid-career female nurses: A prospective cohort study. Occup. Environ. Med..

[47] Dumas O., Varraso R., Boggs K.M., Quinot C., Zock J.P., Henneberger P.K., Speizer F.E., Le Moual N., Camargo C.A. (2019). Association of occupational exposure to disinfectants with incidence of chronic obstructive pulmonary disease among US female nurses. JAMA Netw. Open.

[48] Patel J., Gimeno Ruiz de Porras D., Mitchell L.E., Patel R.R., De Los Reyes J., Delclos G.L. (2020). Work-related asthma among certified nurse aides in Texas. Workplace Health Saf..

[49] Delclos G., Arif A., Aday L., Carson A., Lai D., Lusk C., Stock T., Symanski E., Whitehead L., Benavides F. (2006). Validation of an asthma questionnaire for use in healthcare workers. Occup. Environ. Med..

[50] Wang Z., Taylor K., Allman-Farinelli M., Armstrong B., Askie L., Ghersi D., McKenzie J., Norris S.L., Page M.J., Rooney A. (2019). A Systematic Review: Tools for Assessing Methodological Quality of Human Observational Studies. https://osf.io/preprints/metaarxiv/pnqmy/.

[51] Boulet L.-P. (2013). Asthma and obesity. Clin. Exp. Allergy.

[52] Dumas O., Varraso R., Zock J.P., Henneberger P.K., Speizer F.E., Wiley A.S., Le Moual N., Camargo C.A. (2015). Asthma history, job type and job changes among US nurses. Occup. Environ. Med..

[53] Cormier M., Lemière C. (2020). Occupational asthma. Int. J. Tuberc. Lung Dis..

[54] Wendeler D., Dulon M., Nienhaus A. (2018). 1 Unfälle und Berufskrankheiten im Jahr 2016 bei der Berufsgenossenschaft für Gesundheitsdienst und Wohlfahrtspflege. Risiken Ressour. Gesundh. Wohlfahrtspfl. Bd. 3.

[55] Dulon M. (2020). Personal communication.

[56] Schneider S. (2020). Personal Communication.

[57] MacKinnon M., To T., Ramsey C., Lemière C., Lougheed M.D. (2020). Improving detection of work-related asthma: A review of gaps in awareness, reporting and knowledge translation. Allergy Asthma Clin. Immunol..

