# Supplementary material for: Are Healthcare Workers at an Increased Risk for Obstructive Respiratory Diseases Due to Cleaning and Disinfection Agents? A Systematic Review and Meta-Analysis
Source: Int J Environ Res Public Health. 2021 May 13;18(10):5159. doi: 10.3390/ijerph18105159 (PMC8152277; doi:10.3390/ijerph18105159)

Figure S1. Directed acyclic graph of the causal model between healthcare worker's exposure to cleaning/disinfectant agents (E) and chronic respiratory diseases (O).

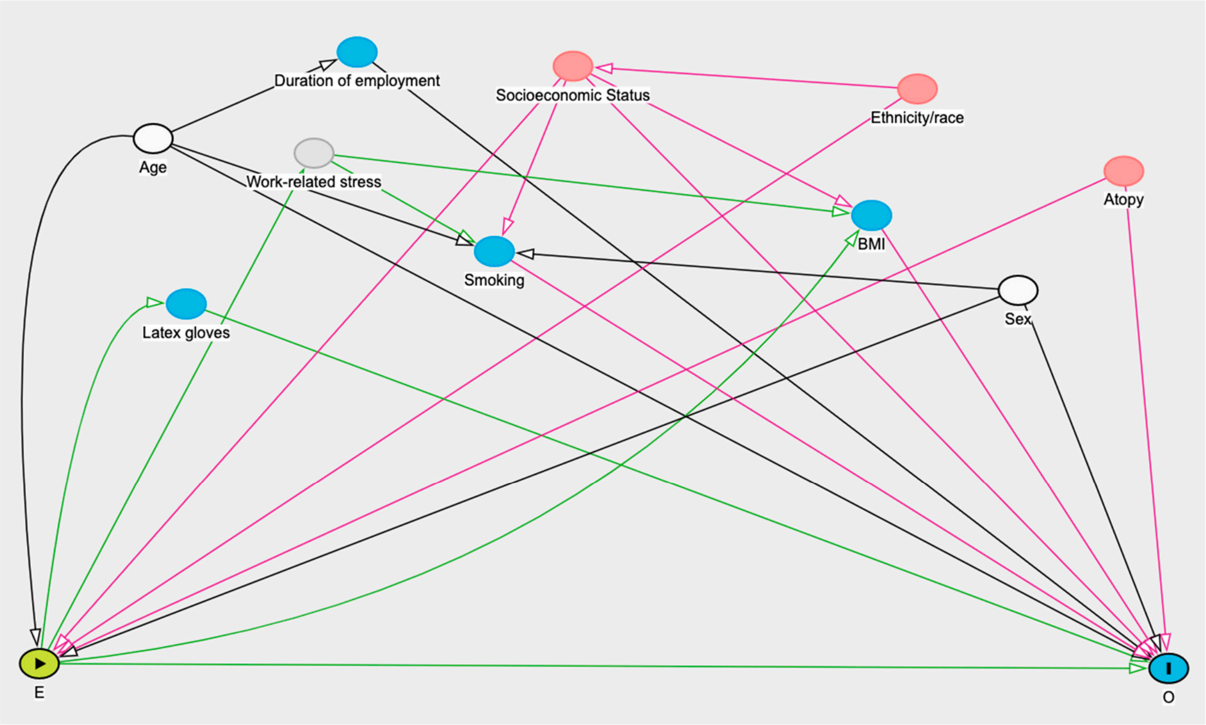

Figure S2. Funnel plot of studies investigating the risk of new-onset asthma in nurses

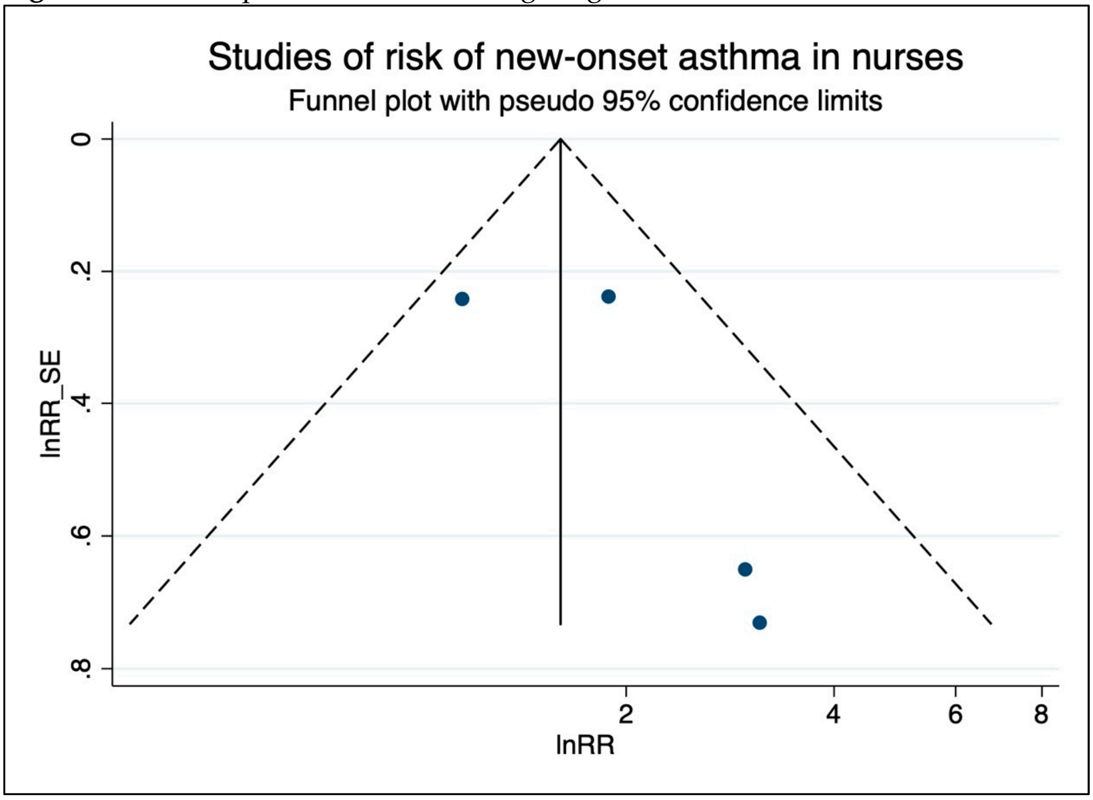

Figure S3. Funnel plot of studies investigating the risk of new-onset asthma in nurses

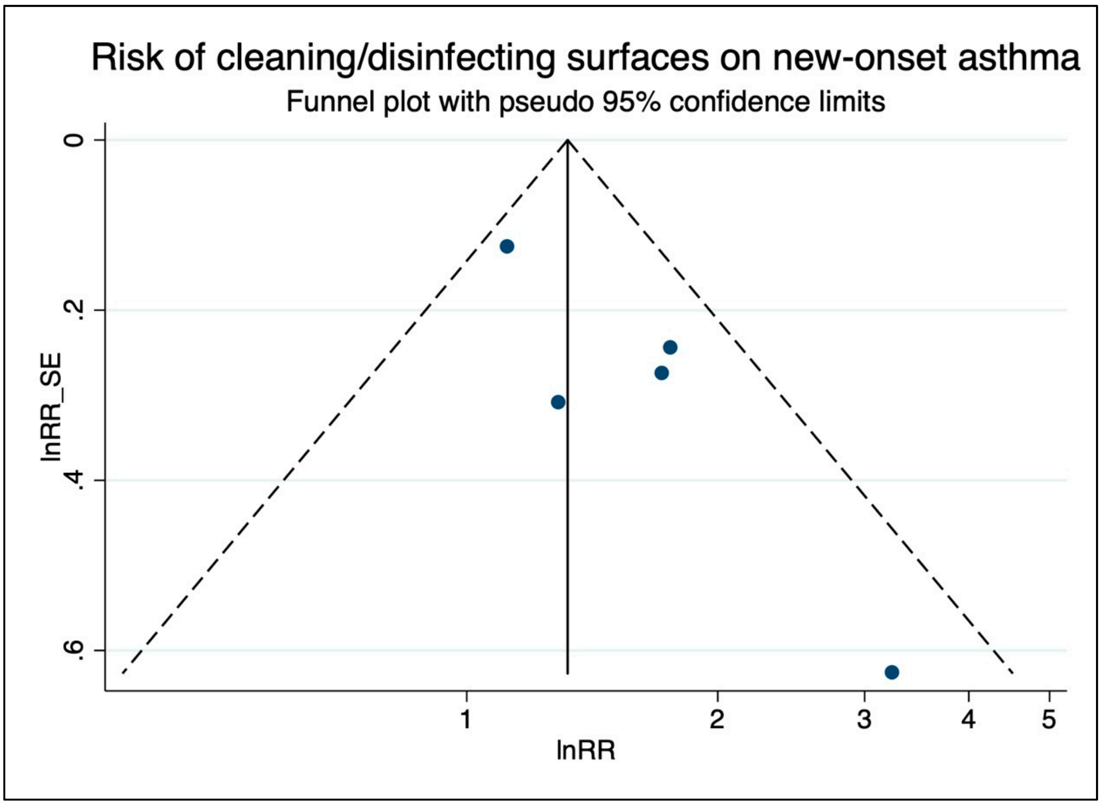

Figure S4. Forest plot of studies investigating the risk of cleaning/disinfecting surfaces for new-onset asthma (sensitivity analysis using disinfection in Gonzalez et al. 2014)

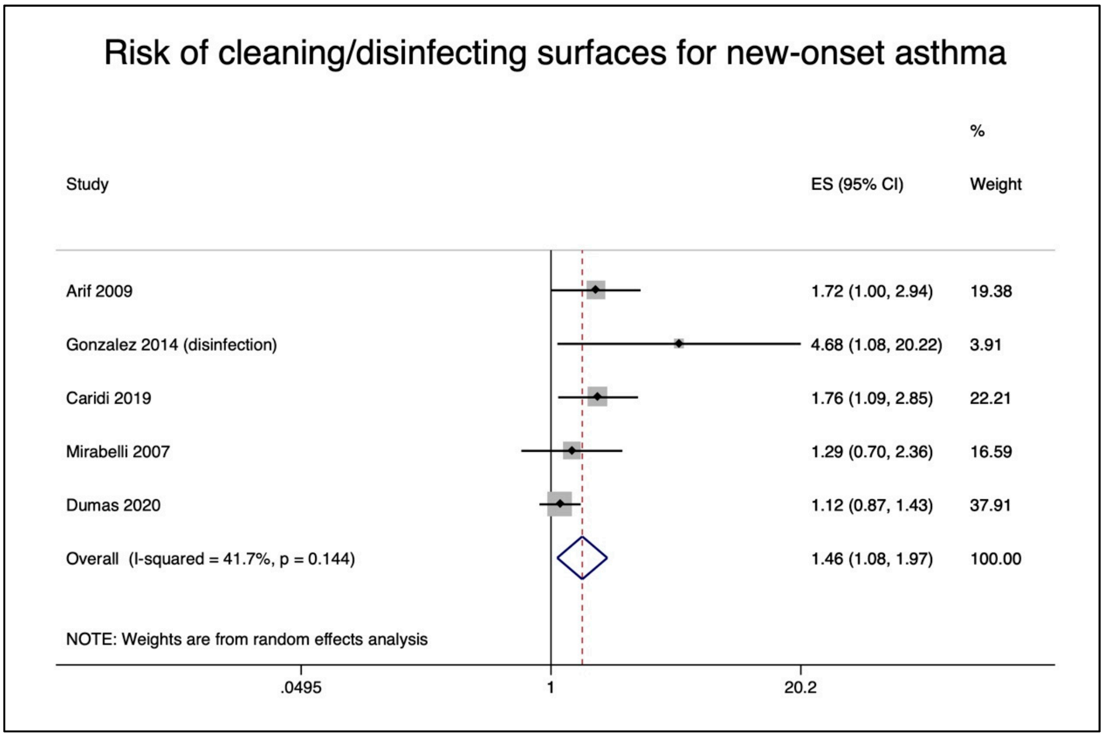

Figure S5. Forest plot of studies investigating the risk of instrument cleaning/disinfection/sterilization for new-onset asthma (sensitivity analysis using exposure to high level disinfectants for more than 5 years, Dumas et al. 2021)

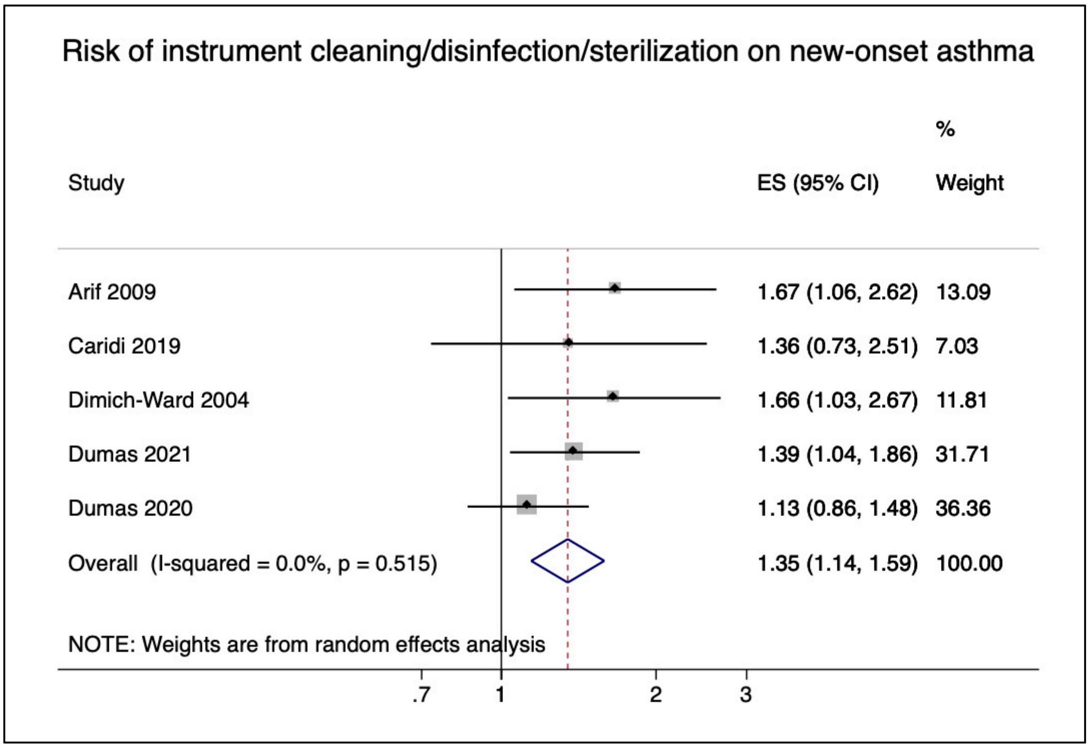

Figure S6. Forest plot of studies investigating the risk of use of adhesive/solvents/chemicals in patient care on new-onset asthma

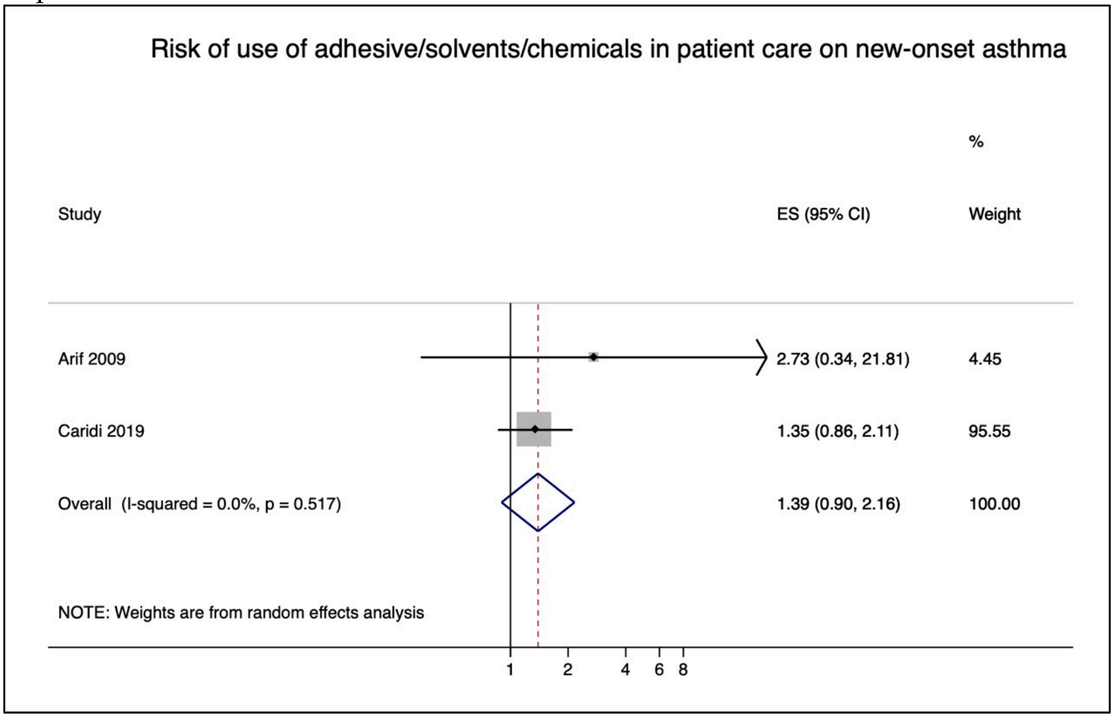

Figure S7. Forest plot of studies investigating the risk of exposure to bleach on asthma (sensitivity analysis using work-exacerbated asthma for Arif et al. 2012)

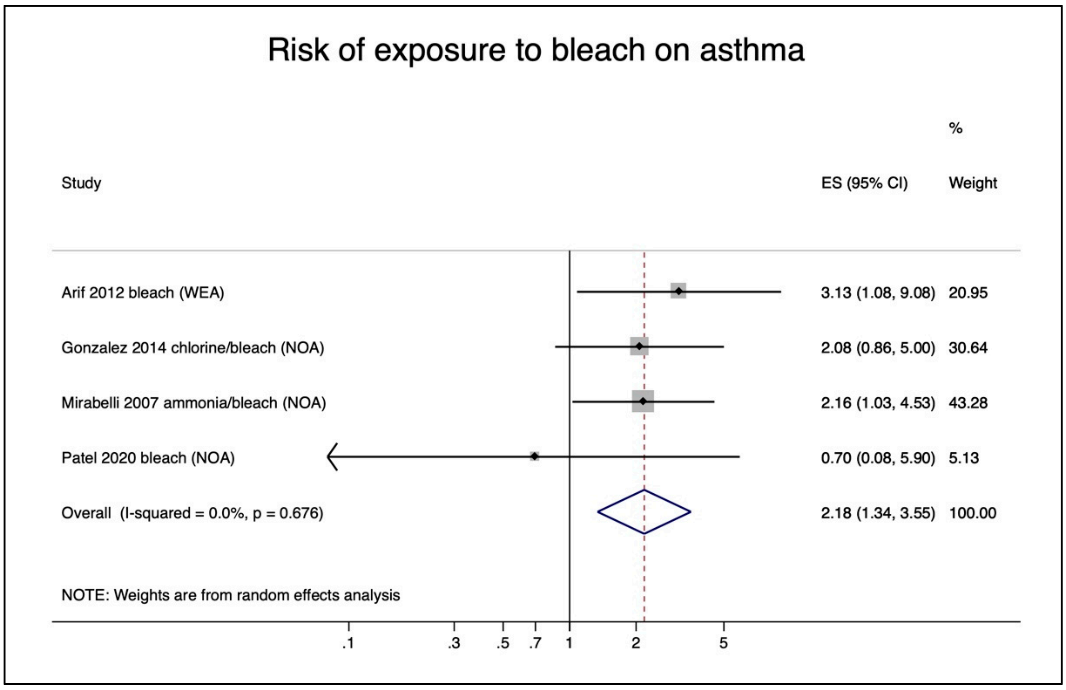

WEA: work-exacerbated asthma; NOA: new-onset asthma

Figure S8. Forest plot of studies investigating the risk of exposure to bleach on asthma (sensitivity analysis using occupational asthma for Arif et al. 2012)

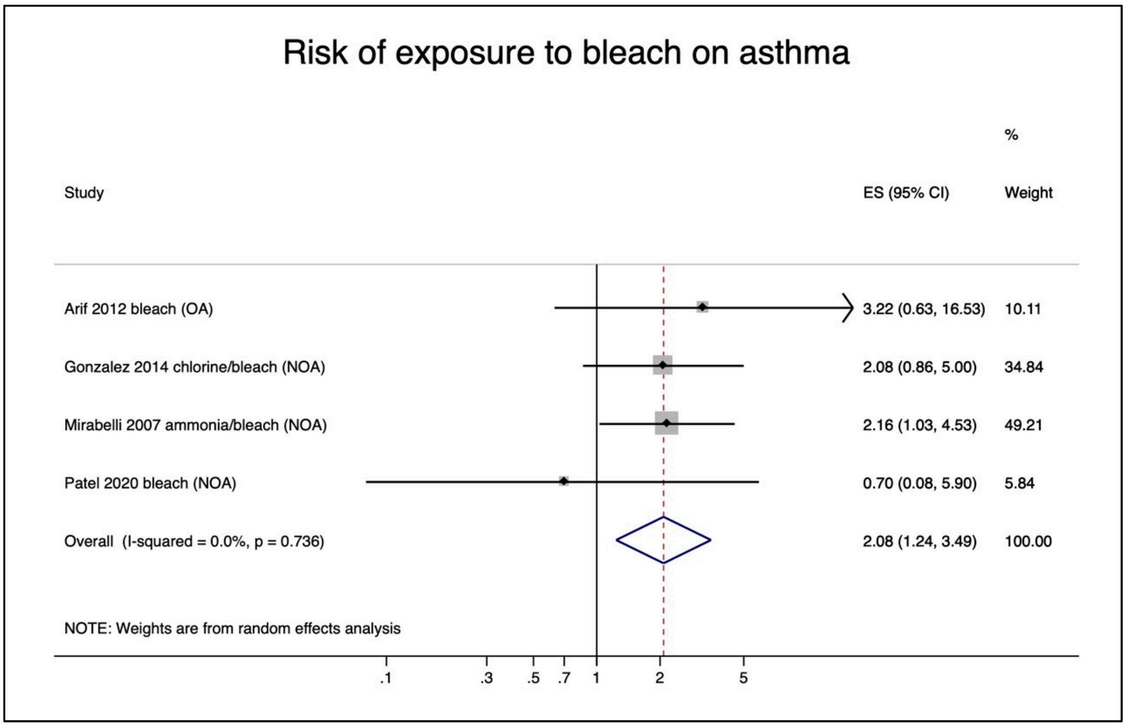

OA: occupational asthma; NOA: new-onset asthma

Figure S9. Forest plot of studies investigating the risk of exposure to glutaraldehyde on asthma (using work-related asthma symptoms for Arif et al. 2012)

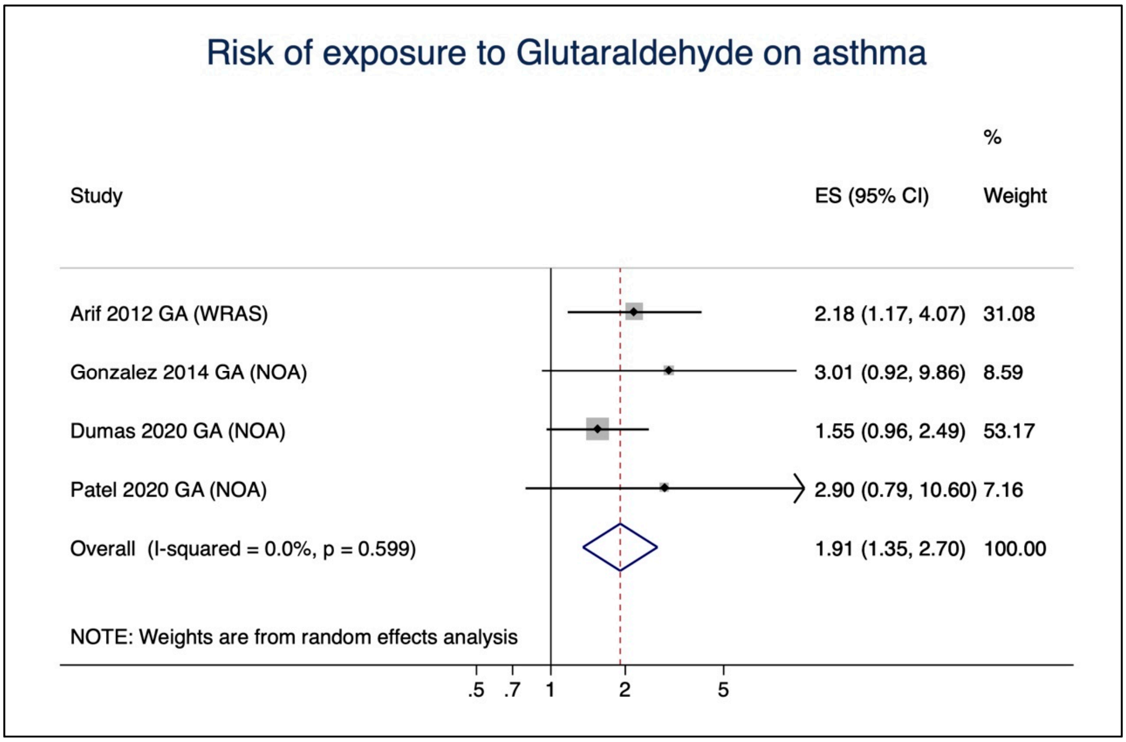

WRAS: work-related asthma; NOA: new-onset asthma

Figure S10. Forest plot of studies investigating the risk of exposure to glutaraldehyde on asthma (using occupational work-exacerbated asthma symptoms for Arif et al. 2012)

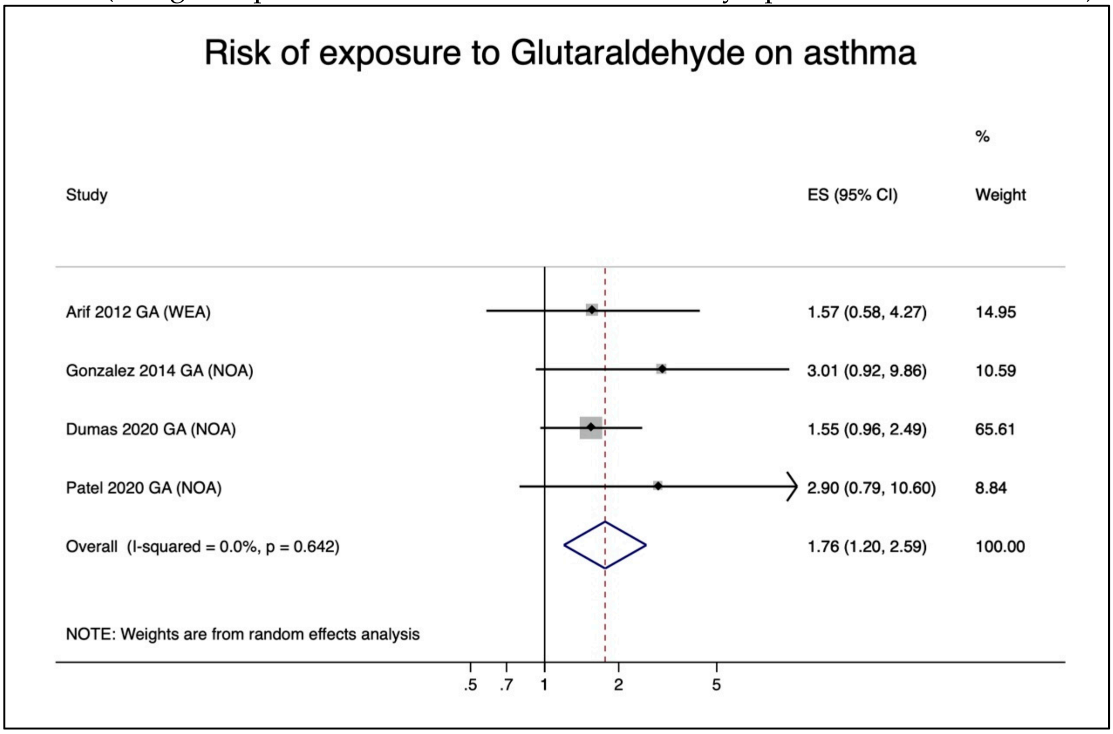

WEA: work-exacerbated asthma; NOA: new-onset asthma

Figure S11. Forest plot of studies investigating the risk of exposure to glutaraldehyde on asthma (using occupational asthma symptoms for Arif et al. 2012)

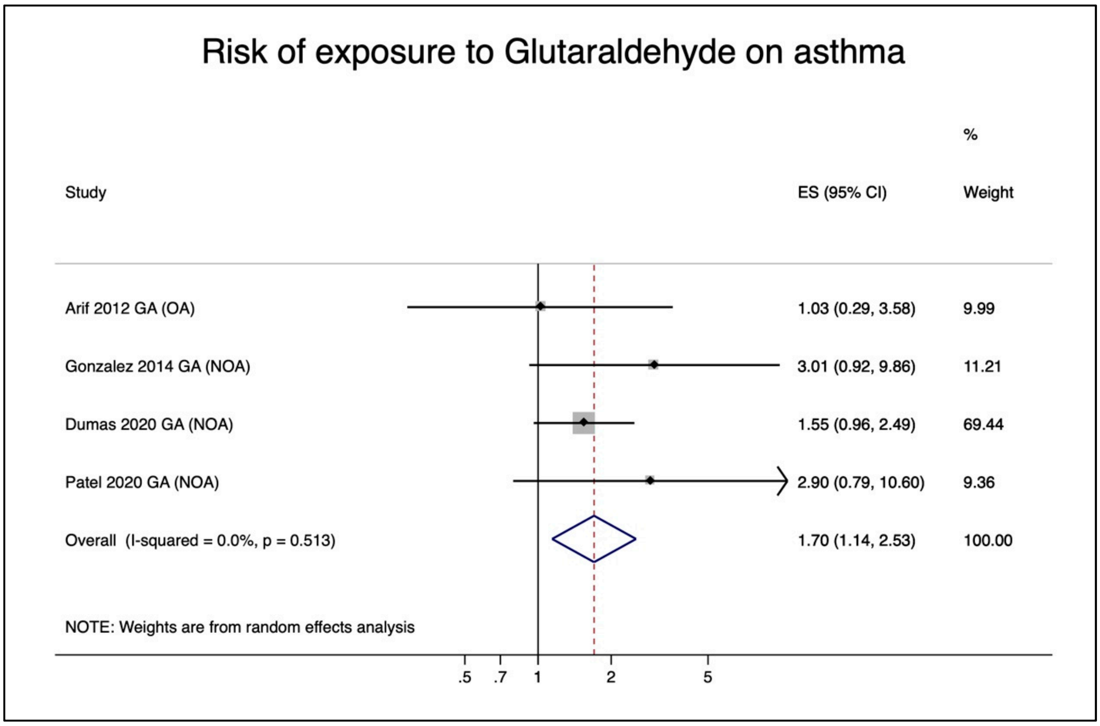

OA: occupational asthma; NOA: new-onset asthma

Figure S12. Forest plot of studies investigating the risk of cleaning/sterilizing equipment on bronchial hyperresponsiveness (BHR)- related symptoms

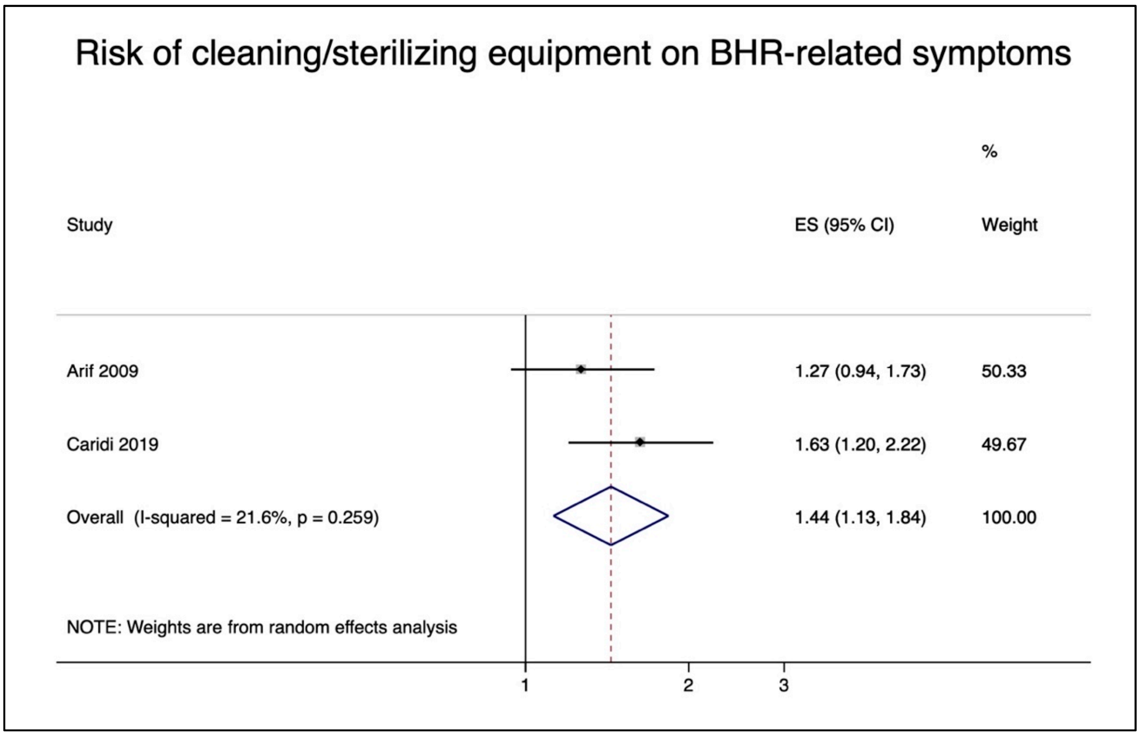

Figure S13. Forest plot of studies investigating the risk of use of adhesive/solvents/chemicals in patient care on bronchial hyperresponsiveness (BHR)- related symptoms

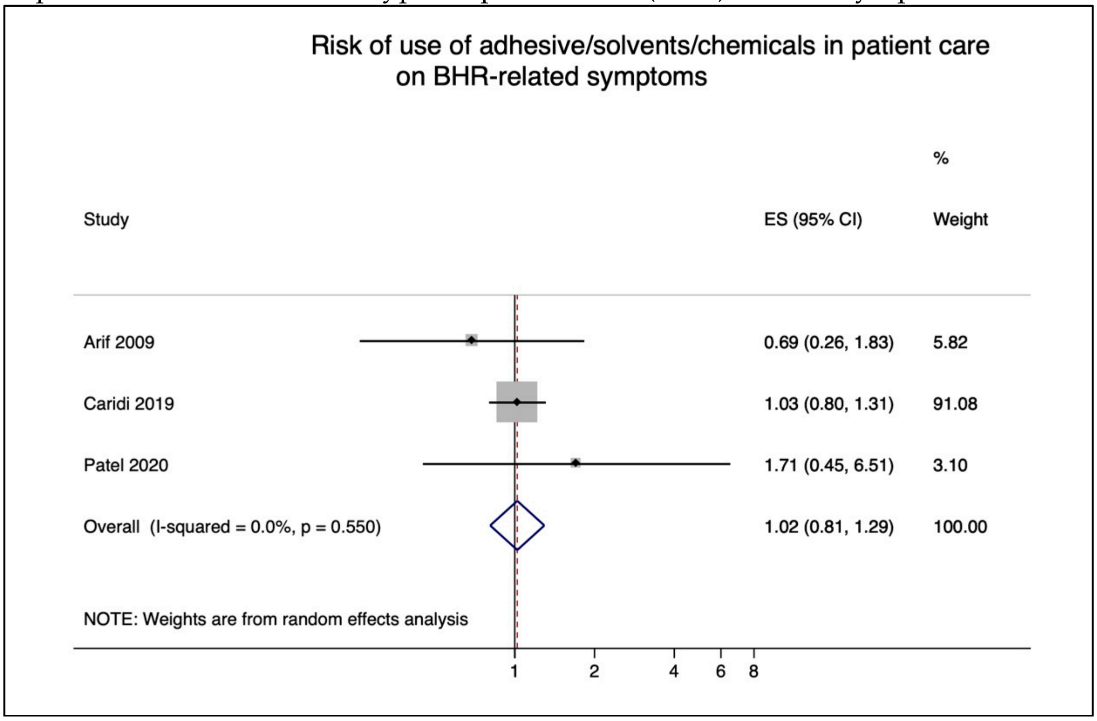

Supplement: Supplementary file 1 [file ijerph-18-05159-s001.zip › Supplementary Figures.pdf]
