# Supplementary material for: Are Healthcare Workers at an Increased Risk for Obstructive Respiratory Diseases Due to Cleaning and Disinfection Agents? A Systematic Review and Meta-Analysis
Source: Int J Environ Res Public Health. 2021 May 13;18(10):5159. doi: 10.3390/ijerph18105159 (PMC8152277; doi:10.3390/ijerph18105159)
Supplement: Supplementary file 1 [file ijerph-18-05159-s001.zip › Supplementary Tables.pdf]

Table S1. Excluded studies and reasons for exclusion

Reference (e: exposition; c: comparison; o: outcome; p: publication; n: no response given; r: response<10% or convenience sample; s: study design; nr: subject not relevant; cp: conference/poster; yr: year)	Reason for exclusion									
	e	c	o	p	n	r	s	nr	cp	yr
Adisesh A, Murphy E, Barber CM, Ayres JG (2011) Occupational asthma and rhinitis due to detergent enzymes in healthcare Occupational medicine (Oxford, England) 61:364-369 doi:http://dx.doi.org/10.1093/occmed/kqr107							x			
Ahmed-Lecheheb D, Cunat L, Hartemann P, Hautemaniere A (2012) Dermal and pulmonary absorption of ethanol from alcohol-based hand rub The Journal of hospital infection 81:31-35 doi:10.1016/j.jhin.2012.02.006	x									
Alanko K, Susitaival P, Jolanki R, Kanerva L (2004) Occupational skin diseases among dental nurses Contact Dermatitis 50:77-82 doi:10.1111/j.0105-1873.2004.00304.x	x									
Ameille J, Hamelin K (2011) Occupational rhinitis in workers with occupational asthma Allergy: European Journal of Allergy and Clinical Immunology 94):590-591 doi:http://dx.doi.org/10.1111/j.1398-9995.2011.02608.x									x	
Ameille J, Larbanois A, Descatha A, Vandenplas O (2006) [Epidemiology and etiologic agents of occupational asthma] Rev Mal Respir 23:726-740							x			
Arif A, Whitehead L, Delclos G, Tortolero S, Lee E (2002) Prevalence and risk factors of work related asthma by industry among United States workers: data from the third national health and nutrition examination survey (1988–94) Occupational and environmental medicine 59:505-511			x							
Arif AA, Delclos GL (2011) Cleaning-related chemicals and work-related asthma in healthcare professionals American Journal of Respiratory and Critical Care Medicine Conference: American Thoracic Society International Conference, ATS 183									x	
Arif AA, Delclos GL, Whitehead LW, Tortolero SR, Lee ES (2003) Occupational exposures associated with work-related asthma and work-related wheezing among U.S. workers American Journal of Industrial Medicine 44:368-376 doi:http://dx.doi.org/10.1002/ajim.10291	x									
Baelum J et al. (2016) Gender, occupation, and prescription of medicine for asthma Occupational and Environmental Medicine 73 (Supplement 1):A216 doi:http://dx.doi.org/10.1136/oemed-2016-103951.599									x	
Bakerly ND, Moore VC, Vellore AD, Jaakkola MS, Robertson AS, Burge PS (2008) Fifteen-year trends in occupational asthma: data from the Shield surveillance scheme Occup Med (Lond) 58:169-174 doi:10.1093/occmed/kqn007								x		
Balkova B, Lebedova J, Klusackova P, Hurtova L, Fenclova Z (2011) Occupational asthma - 13 years experience in the Czech Republic Allergy: European Journal of Allergy and Clinical Immunology 94):591 doi:http://dx.doi.org/10.1111/j.1398-9995.2011.02608.x									x	
Balmes JR, Estacio PL, Quinlan P, Kelly T, Corkery K, Blanc P (1995) Respiratory effects of occupational exposure to aerosolized pentamidine Journal of occupational and environmental medicine 37:145-150	x									
Barnes S, Stuart R, Redley B. Health care worker sensitivity to chlorhexidine-based hand hygiene solutions: A cross-sectional survey. American journal of infection control. 2019 Aug 1;47(8):933-7.						x				
Batllo M, Siret A, Bousquet PJ, Dhivert-Donnadieu H, Demoly P, Hemery ML (2009) Prevalence and risk factors of respiratory and cutaneous symptoms in healthcare workers of several medical centers of Herault region in France. [French] Revue Francaise d'Allergologie 49:2-9 doi:http://dx.doi.org/10.1016/j.allerg.2008.10.006						x				
Benke G, Sim MR, McKenzie DP, MacFarlane E, Del Monaco A, Hoving JL, Fritschi L (2008) Comparison of first, last, and longest-held jobs as surrogates for all jobs in estimating cumulative exposure in cross-sectional studies of work-related asthma Annals of epidemiology 18:23-27	x									

Reference (e: exposition; c: comparison; o: outcome; p: publication; n: no response given; r: response<10% or convenience sample; s: study design; nr: subject not relevant; cp: conference/poster; yr: year)	Reason for exclusion									
	e	c	o	p	n	r	s	nr	cp	yr
Carder M, Seed MJ, Money A, Agius RM, van Tongeren M. Occupational and work-related respiratory disease attributed to cleaning products. Occupational and environmental medicine. 2019 Aug 1;76(8):530-6.		x								
Casey ML, Hawley B, Edwards N, Cox-Ganser JM, Cummings KJ (2017) Health problems and disinfectant product exposure among staff at a large multispecialty hospital American Journal of Infection Control 45:1133-1138 doi:http://dx.doi.org/10.1016/j.ajic.2017.04.003				x						
Center for Disease Control and Prevention (CDC) (2010) Acute antimicrobial pesticide-related illnesses among workers in health-care facilities - California, Louisiana, Michigan, and Texas, 2002-2007 Mmwr Morbidity and mortality weekly report. 59:551-556								x		
Christiani DC, Kern DG (1993) Asthma risk and occupation as a respiratory therapist American Journal of Respiratory and Critical Care Medicine 148:671-674								x		
Crivellaro MA, MacUlan P, Carrara T, Pellegrini MG, Ottaviano G (2018) Allergic respiratory disease in healthcare workers: If not latex, what else? Allergy: European Journal of Allergy and Clinical Immunology 73 (Supplement 105):816-817 doi:http://dx.doi.org/10.1111/all.13540									x	
Donnay C et al. (2011) Under-estimation of self-reported occupational exposure by questionnaire in hospital workers Occupational and Environmental Medicine 68:611-617 doi:http://dx.doi.org/10.1136/oem.2010.061671								x		
Dumas O, Donnay C, Heederik DJ, Hery M, Choudat D, Kauffmann F, Le Moual N (2012) Occupational exposure to cleaning products and asthma in hospital workers Occup Environ Med 69:883-889 doi:10.1136/oemed-2012-100826				x						
Dumas O et al. (2017a) Late Breaking Abstract-Occupational exposure to disinfectants and COPD incidence in US nurses: A prospective cohort study European Respiratory Journal Conference: European Respiratory Society International Congress, ERS 50 doi:http://dx.doi.org/10.1183/1393003.congress-2017.OA1774									x	
Dumas O et al. (2014) Asthma history, job type, and job changes among U.S. Nurses European Respiratory Journal Conference: European Respiratory Society Annual Congress 44									x	
Dumas O et al. (2017b) Occupational exposure to disinfectants and asthma control in US nurses European Respiratory Journal 50 (4) (no pagination) doi:http://dx.doi.org/10.1183/13993003.00237-2017			x							
Fujita H, Sawada Y, Ogawa M, Endo Y (2007) [Health hazards from exposure to ortho-phthalaldehyde, a disinfectant for endoscopes, and preventive measures for health care workers] Sangyo eiseigaku zasshi = Journal of occupational health 49:1-8				x						
Garrido AN, House R, Lipszyc JC, Liss GM, Holness DL, Tarlo SM. Cleaning Agent Usage in Healthcare Professionals and Relationship to: Lung and Skin Symptoms. Journal of Asthma. 2021 Jan 6:1-3				x						
Gonzalez M, Kopferchmitt M, Donnay C, Hedelin G, Velten M, Cantineau A, De Blay F (2010) Asthma and exposure to quaternary ammonium compounds in healthcare settings: Preliminary results of the FRAPSA study Allergy: European Journal of Allergy and Clinical Immunology 92):9-10 doi:http://dx.doi.org/10.1111/j.1398-9995.2010.02391.x									x	
Gonzalez M, Kopfferschmitt MC, Donnay C, Hedelin G, Velten M, Cantineau A, De Blay F (2011) Asthma and exposure to quaternary ammonium compounds in healthcare settings European Respiratory Journal Conference: European Respiratory Society Annual Congress 38									x	

Reference (e: exposition; c: comparison; o: outcome; p: publication; n: no response given; r: response<10% or convenience sample; s: study design; nr: subject not relevant; cp: conference/poster; yr: year)	Reason for exclusion									
	e	c	o	p	n	r	s	nr	cp	yr
Henneberger PK et al. (2018) Work tasks and occupations as risk factors for asthma in a sample of urban healthcare workers European Respiratory Journal Conference: European Respiratory Society International Congress, ERS 52 doi:http://dx.doi.org/10.1183/13993003.congress-2018.OA195									x	
Henneberger PK, Humann MJ, Liang X, Su FC, Stefaniak AB, LeBouf RF, Stanton ML, Virji MA. The Association of Exacerbation of Current Asthma with Work Tasks in a Sample of Healthcare Workers. InA26. ENVIRONMENTAL MODIFIERS OF LUNG DISEASE 2019 May (pp. A1169-A1169). American Thoracic Society.									x	
Knezevic H (2018) Prevalence of allergy related skin and respiratory diseases among healthcare workers in Croatia Occupational and Environmental Medicine 75 (Supplement 2):A53 doi:http://dx.doi.org/10.1136/oemed-2018-ICOHabstracts.150									x	
Kopferschmitt-Kubler MC, Ameille J, Popin E, Calastreng-Crinquand A, Vervloet D, Bayeux-Dunglas MC, Pauli G (2002) Occupational asthma in France: a 1-yr report of the observatoire National de Asthmes Professionnels project The European respiratory journal 19:84-89			x							
Kujala V, Reijula K, Ruotsalainen E, Heikkinen K (1995) Occupational asthma due to chloramine-T solution Respiratory medicine 89:693-695							x			
Kurth L, Virji MA, Storey E, Framberg S, Kallio C, Fink J, Laney AS (2017) Current asthma and asthma-like symptoms among workers at a Veterans Administration Medical Center International journal of hygiene and environmental health 220:1325-1332 doi:http://dx.doi.org/10.1016/j.ijheh.2017.09.001						x				
Le Moual N et al. (2015) Self-reported occupational exposure to disinfectants and asthma control in U.S. Nurses European Respiratory Journal Conference: European Respiratory Society Annual Congress 46 doi:http://dx.doi.org/10.1183/13993003.congress2015.PA2011									x	
Mirabelli MC, London SJ, Charles LE, Pompeii LA, Wagenknecht LE (2012) Occupation and three-year incidence of respiratory symptoms and lung function decline: The ARIC Study Respiratory Research 13 (no pagination) doi:http://dx.doi.org/10.1186/1465-9921-13-24	x									
Montgomery AB, Corkery KJ, Brunette ER, Leoung GS, Waskin H, Debs RJ (1990) Occupational exposure to aerosolized pentamidine Chest 98:386-388	x									
Mwanga HH, Baatjies R, Jeebhay MF (2018) Exposure to aldehydes among health care workers in a large Tertiary Hospital in Cape Town, South Africa Occupational and Environmental Medicine 75 (Supplement 2):A337 doi:http://dx.doi.org/10.1136/oemed-2018-ICOHabstracts.966									x	
Mwanga HH, Baatjies R, Singh T, Jeebhay MF. Occupational risk factors for work-related asthma outcomes in health workers exposed to diverse cleaning agents. InALLERGY 2020 Aug 1 (Vol. 75, pp. 230-230). 111 RIVER ST, HOBOKEN 07030-5774, NJ USA: WILEY.									x	
Norback D (1988) Skin and respiratory symptoms from exposure to alkaline glutaraldehyde in medical services Scandinavian journal of work, environment & health 14:366-371			x							x
Pechter E et al. (2005) Work-related asthma among health care workers: Surveillance data from California, Massachusetts, Michigan, and New Jersey, 1993-1997 American Journal of Industrial Medicine 47:265-275 doi:http://dx.doi.org/10.1002/ajim.20138		x								

**Reference** (e: exposition; c: comparison; o: outcome; p: publication; n: no response given; r: response<10% or convenience sample; s: study design; nr: subject not relevant; cp: conference/poster; yr: year)	**Reason for exclusion**									
	**e**	**c**	**o**	**p**	**n**	**r**	**s**	**nr**	**cp**	**yr**
Quinot C et al. (2017) Development of a job-task-exposure matrix to assess occupational exposure to disinfectants among US nurses Occupational and Environmental Medicine 74:130-137 doi:http://dx.doi.org/10.1136/oemed-2016-103606								x		
Quinot C et al. (2015) Job-task-exposure matrices to assess occupational exposure to disinfectants among U.S. Nurses European Respiratory Journal Conference: European Respiratory Society Annual Congress 46 doi:http://dx.doi.org/10.1183/13993003.congress2015.PA2023									x	
Rideout K, Teschke K, Dimich-Ward H, Kennedy SM (2005) Considering risks to healthcare workers from glutaraldehyde alternatives in high-level disinfection Journal of Hospital Infection 59:4-11 doi:http://dx.doi.org/10.1016/j.jhin.2004.07.003								x		
Reilly MJ, Wang L, Rosenman KD. The Burden of Work-related Asthma in Michigan, 1988–2018. Annals of the American Thoracic Society. 2020 Mar;17(3):284-92.				x						
Rosenman KD et al. (2003) Cleaning products and work-related asthma Journal of Occupational and Environmental Medicine 45:556-563 doi:http://dx.doi.org/10.1097/01.jom.0000058347.05741.f9	x									
Rosenman K, Reilly MJ, Pechter E, Fitzsimmons K, Flattery J, Weinberg J, Cummings K, Borjan M, Lumia M, Harrison R, Dodd K. Cleaning Products and work-related asthma, 10 year update. Journal of occupational and environmental medicine. 2020 Feb 1;62(2):130-7.				x						
Sama SR, Milton DK, Hunt PR, Houseman EA, Henneberger PK, Rosiello RA (2006) Case-by-case assessment of adult-onset asthma attributable to occupational exposures among members of a health maintenance organization Journal of occupational and environmental medicine 48:400-407	x									
Silver SR, Alarcon WA, Li J. Incident chronic obstructive pulmonary disease associated with occupation, industry, and workplace exposures in the Health and Retirement Study. American Journal of Industrial Medicine. 2021 Jan;64(1):26-38.	x									
Su F et al. (2018) Clustering respiratory symptoms and cleaning and disinfecting activities and evaluating their associations among healthcare workers American Journal of Respiratory and Critical Care Medicine Conference: American Thoracic Society International Conference, ATS 197									x	
Suuronen K, Sauni R, Jaakkola MS (2011) [Assessment of exposure in suspected occupational asthma] Duodecim 127:2215-2222							x			
Virji MA, LeBouf R, Saito R, Stefaniak A, Stanton M, Henneberger P (2011) Assessing exposures to cleaning and disinfecting chemicals for an epidemiologic study of asthma in healthcare occupations Occupational and Environmental Medicine 1):A79-A80 doi:http://dx.doi.org/10.1136/oemed-2011-100382.260									x	
Walters GI, Burge PS, Moore VC, Robertson AS (2018) Cleaning agent occupational asthma in the West Midlands, UK: 2000-16 Occupational medicine (Oxford, England) 68:530-536 doi:http://dx.doi.org/10.1093/occmed/kqy113			x							
Walters GI, Moore VC, McGrath EE, Burge PS, Henneberger PK (2013) Agents and trends in health care workers' occupational asthma Occup Med (Lond) 63:513-516 doi:10.1093/occmed/kqt093		x								
Waters A, Beach J, Abramson M (2003) Symptoms and lung function in health care personnel exposed to glutaraldehyde American Journal of Industrial Medicine 43:196-203 doi:http://dx.doi.org/10.1002/ajim.10172						x				
Weber DJ, Consoli SA, Rutala WA (2016) Occupational health risks associated with the use of germicides in health care Am J Infect Control 44:e85-89 doi:10.1016/j.ajic.2015.11.030							x			
Zock J-P et al. (2004) Evaluation of specific occupational asthma risks in a community-based study with special reference to single and multiple exposures Journal of Exposure Science & Environmental Epidemiology 14:397-403	x									

Table S2. Risk of bias instrument

Major risk of bias domains*	Risk	Criteria	Hints/ notes
**1. Recruitment procedure & follow-up (in cohort studies)**:<br><br>***For cohort studies***<br><br>*HINT: We are looking for selection bias:*<br>- *Was the cohort representative of a defined population? #*<br>- *Was everybody included who should have been included? #*<br><br>*PRELIMINARY RULING:*<br>- *If the cohort recruitment is based on a convenient/ self-reported sampling OR if response is <10%, the study will be excluded from analysis.*	low	☐ Cohort recruitment was acceptable. #<br>☐ Baseline response on both educator and day care centre level is acceptable (50% or more) OR is <50% and >30%, but substantial differential selection could be excluded (e. g. by a non-responder analysis).<br>☐ Loss to follow-up is below 20% in total and not different between the two groups (up to 10% difference). *	
	high	☐ Cohort recruitment was not acceptable. #<br>☐ Response not reported/ not calculable.<br>☐ Total loss to follow-up is larger than acceptable (20% or more) * OR drop out differs between the groups by more than 10%* OR the reasons for drop out considerably differ between exposed and non-exposed groups. *	
For case-control studies<br><br>*HINT: We are looking for selection bias:*<br>- *Were the cases and control subjects representative of the same defined population ("study base"; geographically and/or temporally)? #*<br>- *Was there an established reliable system for selecting all the cases? #*<br>- *The same exclusion criteria are used for both cases and controls. #*<br>- *Comparison is made between participants and non-participants to establish their similarities or differences. #*<br><br>*.PRELIMINARY RULING:*<br>- *If the recruitment is based on a convenient/ self-reported sampling OR*	low	☐ Case selection and recruitment were acceptable. #<br>☐ Control subjects' selection and recruitment were acceptable. #<br>☐ Non-response was less than 50% for cases and/or control subjects OR it was >50% and <70%, but substantial differential selection of cases and control subjects could be excluded (e.g., by a non-responder analysis) *	
	high	☐ Case selection and recruitment were not acceptable. #<br>☐ Control subjects' selection and recruitment were not acceptable. #<br>☐ Non-response was >70% for cases or control subjects OR it was >50% and<70%, but substantial differential selection of cases and control subjects could not be excluded. *<br>☐ Response not reported/ not calculable	

Major risk of bias domains*	Risk	Criteria	Hints/ notes
if response is <10%, the study will be excluded from analysis.			
For cross-sectional studies<br><br>*HINT: We are looking for selection bias:*<br><br>- *Was the study population representative of a defined population? #*<br>- *Was everybody included who should have been included? #*<br><br>*PRELIMINARY RULING:*<br><br>- *If the recruitment is based on a convenient/ self-reported sampling OR if response is <10%, the study will be excluded from analysis.*	low	☐ Recruitment of the study population was acceptable. #<br>☐ Non-response was less than 50% OR it was >50% and <70%, but substantial differential selection of the study population could be excluded (e.g., by a non-responder analysis). *	
	high	☐ Recruitment of the study population was not acceptable. #<br>☐ Non-response was >70% OR it was >50% and <70%, but substantial differential selection of the study population could not be excluded. *<br>☐ Response not reported/ not calculable.	
**2. Exposure definition and measurement**	low	☐ Exposure definition included at least basic job characteristics (e.g., job tasks, length of employment).<br>☐ Exposure was accurately measured to minimize bias: use of external validation, validated questionnaire or exposure is connected to a task) #<br>☐ Adequate comparison group of non-exposed workers (e.g., office workers) included.	
	high	☐ Exposure does not cover basic job characteristics.<br>☐ Exposure was not accurately measured (e.g., use of JEM only) #<br>☐ Different methods were used to measure exposure in different groups/ cases and control subjects (***in case-control studies***).<br>☐ No adequate comparison group of non-exposed workers included	
	unclear	☐ Not reported.	
**3.1 Outcome "obstructive disease" (e.g., asthma)**<br>**Source and validation**	low	☐ Outcome was accurately/ objectively measured to minimize bias (pulmonary function test, medical diagnosis). #<br>☐ Measurement methods were similar in the different groups. #	
	high	☐ Outcome was not accurately or subjectively measured (self-reported physician diagnosis or questionnaire). #<br>☐ Measurement methods were different in the groups. #	
	unclear	☐ Not reported.	

Major risk of bias domains*	Risk	Criteria	Hints/ notes
**3.2 Outcome "limitation in lung function" (e.g., bronchial hyperresponsiveness). Source and validation**	low	☐ Outcome was accurately/ objectively measured to minimize bias (e.g., pulmonary function test, peak flow, medical diagnosis). #<br>☐ Measurement methods were similar in the different groups.#	
	high	☐ Outcome was not accurately or subjectively measured (e.g., self-reported physician diagnosis or questionnaire). #<br>☐ Measurement methods were different in the groups. #	
	unclear	☐ Not reported.	
**4. Confounding and effect modification**	low	☐ If risk estimators were calculated, major confounding factors (at least age, sex, atopy and SES) were considered.<br>☐ If only prevalence or incidence was assessed, at least sex & age (at least mean values for the study population) are described.	
	high	☐ Major confounding factors or effect modifiers were not considered.	
	unclear	☐ Not reported.	
**5. Analysis method: methods to reduce research specific bias**	low	☐ Authors used adequate statistical models to reduce bias (e.g., standardization, matching, adjustment in multivariate model, stratification, propensity scoring). § For prevalences, matching/stratification may not be required as long as a good description of the age structure and immunization status of the population is given.	
	high	☐ Authors did not use adequate statistical models to reduce bias.	
	unclear	☐ Not reported	
**6. Chronology**<br><br># although incident diseases were inferred due to calculation of number of years of having asthma	low	☐ Incident diseases were included. #<br>☐ Temporal relation may be established (exposure precedes the outcome). #	
	high	☐ Prevalent diseases were included OR prevalent diseases of baseline were not excluded ***(in cohort studies)***. #<br>☐ Temporal relation cannot be established.	
	unclear	☐ Not reported.	

Minor risk of bias domains*	Risk	Criteria	Hints/ notes
**7. Blinding of assessors**	low	☐ Assessors were blinded.	
	high	☐ Assessors were not blinded.	
	unclear	☐ Not reported.	
**8. Funding**	low	☐ Grant/ non-profit-organizations*<br>☐ Study was clearly not affected by sponsors. *	
	high	☐ Sponsoring organization participated in data analysis.<br>☐ Study was probably affected by sponsors.	
	unclear	☐ Industry, combined industry grant*, unclear if study was affected by sponsors.<br>☐ Not reported.	
**9. Conflict of interest**	low	☐ Reported not having conflict of interest or clear from report/ communication that study was not affected by author(s) affiliation. *	

Minor risk of bias domains*	Risk	Criteria	Hints/ notes
	high	☐ Conflict of interest exists (at least one author). *	
	unclear	☐ Not reported.	

Overall risk of bias assessment:		Low Risk	High Risk	Unclear
Major domains	1. Recruitment procedure & follow-up (in cohort studies)			
	2. Exposure definition and measurement			
	3.1 Outcome “obstructive disease”. Source and validation			
	3.2 Outcome “limitation in lung function”. Source and validation			
	4. Confounding and effect modification			
	5. Analysis method: methods to reduce research specific bias			
	6. Chronology			
Minor domains	7. Blinding of assessors			
	8. Funding			
	9. Conflict of interest			
**General rule for rating:**<br>**Low risk of bias:** low risk in all major domains<br>**High risk of bias:** if not low risk	**Overall assessment:**			

Table S3. Characteristics of included studies investigating asthma (Type A)

Study<br><br>Ref [ ]	Study design	Study region	Time and type of recruitment<br><br>Response (%)	Population characteristics			Outcome description and assessment
				Overall	Exposure group	Comparison group	
Arif et al. 2009*	Cross-sectional	Texas, USA	2004<br><br>Random sample of four groups of Texas healthcare professionals (HCPs) with active licenses in 2003<br><br>Overall response: 66%<br>Response nursing professionals (NPs): 70%	N= 3634	NPs (n= 448)<br>comprised of<br>registered nurses: 394 (87.9%)<br>nurse practitioners: 14 (3.1%)<br>licensed vocational nurse: 25 (5.6%)<br>nurse aides: 15 (3.4%)<br><br>Sex<br>Male: 39 (8.8%)<br>Female: 403 (91.2%)<br><br>Age (mean, SD):<br>NPs: 48.6 years, SD 10.7 yrs.<br><br>Mean duration of employment:<br>23.9 yrs. (SD 11.3, median 24 yrs.)<br><br>NPs working in clinical settings (hospitals, private practice, outpatient clinic, nursing home, public school, home health and dental office)<br>n= 396 (88.4%)<br>Working in non-clinical settings (health department, health insurance agency, research, medical sales, academia and others)<br>n= 52 (11.6%)	Other HCPs (n=3186)<br>comprised of<br>physicians: 862 (27.1%)<br>respiratory therapists: 879 (27.6%)<br>occupational therapists: 968 (30.4%)<br>others: 477 (15.0%)<br><br>Sex<br>Male: 1114 (35.5%)<br>Female: 2028 (64.5%)<br><br>Age (mean, SD):<br>other HCPs: 44.9 yrs., SD 12.0 yrs.<br><br>Mean duration of employment:<br>18.6 yrs. (SD 11.8, median 16 yrs.)<br><br>Other HCPs working in clinical settings (hospitals, private practice, outpatient clinic, nursing home, public school, home health and dental office)<br>N= 860 (27%)<br>Working in non-clinical settings (health department, health insurance agency, research, medical sales, academia and others)<br>N= 2326 (73%)	New-onset asthma:<br>asthma after entry into the healthcare profession constructed from 3 questions:<br>„Have you ever had asthma? “<br>„If yes, has your asthma been confirmed by a doctor? “<br>„If yes, at what age was your asthma confirmed by a doctor? “<br>Age of asthma diagnosis compared to number of years in the healthcare profession to determine temporal relationship between asthma and entry to professional<br><br>Outcome assessment:<br>validated questionnaire
Delclos et al. 2007*	Cross-sectional	Texas, USA	2004<br><br>Random sample of four groups of Texas healthcare professionals (HCPs) with active licenses in 2003<br><br>Response<br>overall: 66%	N = 2738<br><br>Number of participants by duration of employment:<br>0-9 yrs.:<br>689 (25.2%)<br>10-16 yrs.:<br>706 (25.8%)	n= 2056<br>nurses: 695 (33.8%)<br>respiratory therapists: 644 (31.3%)<br>occupational therapists: 717 (34.9%)	Physicians<br>n= 682	New-onset (reported) asthma:<br>self-reported physician-diagnosed asthma with onset after entry into the health care profession<br>Age of asthma diagnosis compared to number of years in the healthcare profession to determine temporal

Study<br><br>Ref [ ]	Study design	Study region	Time and type of recruitment<br><br>Response (%)	Population characteristics			Outcome description and assessment
				Overall	Exposure group	Comparison group	
			occupational therapists: 73%<br>respiratory therapists: 65%<br>nurses: 70%<br>physicians: 54%	17-26 yrs.:<br>675 (24.7%)<br>≥27 yrs.:<br>668 (24.4%)<br>Sex<br><br>Female:<br>1803 (75.4%)<br>Male:<br>935 (24.6%)<br><br>Mean age<br>46.7±0.32 yrs.			relationship between asthma and entry to professional<br><br>Outcome assessment:<br>validated questionnaire
Delclos et al. 2009*	Cross-sectional	Texas, USA	2004<br><br>Random sample of four groups of Texas healthcare professionals (HCPs) with active licenses in 2003<br><br>Response<br>Overall: 66%	N= 3650<br><br>Sex<br>Female: 2391 (67.6%)<br>Male: 1145 (32.4%)<br><br>Mean Age (SD)<br>Asthmatics:<br>45.1 yrs. (11.4 yrs.)<br>Non-asthmatics:<br>45.4 yrs. (12.0 yrs.)<br>Mean seniority (SD)<br>Asthmatics:<br>19.1 yrs. (11.5 yrs.)<br>Non-asthmatics:<br>19.2 yrs. (12.0 yrs.)	Nurses<br>n= 926<br>Respiratory therapists<br>n= 869<br>Occupational therapists<br>n= 947	Physicians<br>n= 852	"Asthmatic"<br><br>Outcome assessment:<br>self-reported history of physician-diagnosed asthma or wheezing (aside from colds) in the previous 12 months
Arif and Delclos, 2012*	Cross-sectional	Texas, USA	2004<br><br>Random sample of four groups of Texas healthcare professionals (HCPs) with active licenses in 2003<br><br>Response<br>Overall: 66%	N= 3650<br><br>Sex<br>Female: 2437 (76.1%)<br>Male: 1153 (22.3%)<br><br>Mean age 45.3 yrs.,<br>SD 11.9 yrs.	Nurses:<br>n= 941<br>Respiratory therapists:<br>n= 879<br>Occupational therapists:<br>n= 968	Physicians:<br>n= 682	WRAS (work related asthma symptoms):<br>asthma symptoms at work (wheezing/whistling or shortness of breath) that get better away from work or worsen on return to work,<br>no history of physician-diagnosed asthma

Study<br><br>Ref [ ]	Study design	Study region	Time and type of recruitment<br><br>Response (%)	Population characteristics			Outcome description and assessment
				Overall	Exposure group	Comparison group	
							WEA (work exacerbated asthma):<br>asthma symptoms at work that get better away from work or worsen on return to work with history of physician-diagnosed asthma *before* beginning work as HCP<br><br>OA (occupational asthma):<br>asthma symptoms at work that get better away from work or worsen in return to work with history of physician-diagnosed asthma *after* beginning work as HCP (determined by comparing age at which a physician made diagnosis with years spent in the healthcare profession).<br><br>Outcome assessment:<br>assessment via validated questionnaires
Gonzalez et al.<br>2014	Cross-sectional	France	May 2006 – October 2007<br><br>Multi-center study in 7 French healthcare settings. Stratified random sampling of the various healthcare departments according to activity (surgery/medicine) and sector (public university hospital/private hospital clinics).<br>All staff members aged 18-65 years, working for at least 6 months in the	N= 543<br><br>Sex<br>Female: 474 (88.9%)<br>Male: 59 (11.1%)<br><br>Age:<br>39.9 yrs.<br>(SD=10.5)<br><br>Healthcare sector seniority:<br>0-9 yrs.: 197 (37.2%)<br>10-19 yrs.: 133 (25.0%)	Nurses: 201<br>Auxiliary nurses: 89<br>Cleaners: 94<br><br>Sex<br>Nurses:<br>Male: 10 (5.1%)<br>Female: 187 (94.9%)<br>Auxiliary nurses:<br>Male: 9 (10.5%)<br>Female: 77 (89.5%)<br><br>Age<br>Nurses:<br>0-29 yrs.: 65 (32.3%)	Comparison group used for physician-diagnosed outcome:<br>administrative personnel: 59<br><br>Sex<br>Male: 4 (6.8%)<br>Female: 55 (93.2%)<br><br>Age<br>0-29 yrs.: 7 (11.9%)<br>30-39 yrs.: 12 (20.3%)<br>40-49 yrs.: 22 (37.3%)<br>50+ yrs.: 18 (30.5%)	Physician-diagnosed asthma:<br>Positive responses to `Have you ever had asthma?´ *and* `Was it confirmed by a doctor?´<br><br>New-onset asthma:<br>physician-diagnosed asthma but reported onset after entry into the healthcare sector<br><br>Self-administered questionnaire<br>Symptoms and questions related to asthma selected from validated questionnaires such as European Community

Study<br><br>Ref [ ]	Study design	Study region	Time and type of recruitment<br><br>Response (%)	Population characteristics			Outcome description and assessment
				Overall	Exposure group	Comparison group	
			sampled departments were eligible for inclusion.<br><br>Overall response: 77%	20+ yrs.: 201 (37.9%)	30-39 yrs.: 67 (33.4%)<br>40-49 yrs.: 41 (20.4%)<br>50+ yrs.: 28 (13.9%)<br>Auxiliary nurses:<br>0-29 yrs.: 13 (15.1%)<br>30-39 yrs.: 20 (23.3%)<br>40-49 yrs.: 37 (43%)<br>50+ yrs.: 16 (18.6%)<br><br>Healthcare sector seniority:<br>Nurses:<br>0-9 yrs.: 97 (48.5%)<br>10-19 yrs.: 45 (22.5%)<br>20+ yrs.: 59 (29%)<br>Auxiliary nurses:<br>0-9 yrs.: 28 (31.8%)<br>10-19 yrs.: 26 (29.6%)<br>20+ yrs.: 34 (38.6%)<br><br>Exposure to disinfection tasks<br>Nurses: 195 (97.0%)<br>Auxiliary nurses: 86 (96.6%)<br>Exposure to cleaning tasks<br>Nurses: 167 (84.3%)<br>Auxiliary nurses: 82 (95.3%)	Healthcare sector seniority:<br>0-9 yrs.: 15 (26.3%)<br>10-19 yrs.: 11 (19.3%)<br>20+ yrs.: 31 (54.4%)<br><br>Comparison group used for new-onset asthma:<br>Others: 100 (mainly comprised of physicians, charge nurses, physiotherapists, midwives)<br>Sex<br>Nurses:<br>Male: 36 (36.4%)<br>Female: 63 (63.6%)<br><br>Age<br>0-29 yrs.: 4 (4.0%)<br>30-39 yrs.: 24 (24.0%)<br>40-49 yrs.: 31 (31.0%)<br>50+ yrs.: 41 (41.0%)<br><br>Healthcare sector seniority:<br>0-9 yrs.: 11 (11.0%)<br>10-19 yrs.: 31 (31.0%)<br>20+ yrs.: 58 (58.0%)<br>Exposure to disinfection tasks<br><br>Administrative: 1 (1.7%)<br>Others: 42 (42.4%)<br>Exposure to cleaning tasks<br>Administrative: 2 (3.4%)<br>Others: 26 (26.5%)	Respiratory Health Suvey (ECRHS), the International Study of Asthma and Allergies in Childhood (ISAAC), and Epidemiological Study of Genetics and Environment is Asthma (EGEA)
Mirabelli et al.<br>2007	Cohort	13 European countries	Baseline ECRHS I: 1991<br>Follow-up ECRHS II: 1998-1999<br><br>Population-based, random sample of adults living close	N= 2813<br><br>Sex<br><br>Female: 1709 (61%)<br>Male: 1104 (39%)	Exposure group 1:<br>Nursing related occupations based on ISCO-88:<br>332<br>Comprised of<br>Nursing and midwifery: 86	Professional administrative occupations:<br>2481<br><br>ECRHS II respondents who reported they had not performed any of the	New-onset asthma: symptom-free at baseline (negative answers to: „Have you ever had asthma“, „Have you been woken up by an attack of shortness of breath at any

Study<br><br>Ref [ ]	Study design	Study region	Time and type of recruitment<br><br>Response (%)	Population characteristics			Outcome description and assessment
				Overall	Exposure group	Comparison group	
			to one of the 28 study centers.<br><br>Response<br>Baseline<br>(ECRHS I): screening questionnaire:<br>median 78%<br>ECRHS I main questionnaire:<br>median 65%<br><br>Follow up<br>(ECRHS II) screening questionnaire:<br>median 79%<br>ECRHS II main questionnaire:<br>median 76%<br><br>Participation in ECRHS II random sample and in centers for which occupational survey modules were administered: 58%<br><br>Overall response in final study population:<br>nurses (exposed): 87%<br>administrative (unexposed): 57%	Classification of jobs held for at least three months during the ECRHS follow-up period were classified according to International Standard Classification of Occupations (ISCO-88) codes using verbatim information provided in the occupational history portion of the ECRHS II survey<br>*and*<br>nursing jobs were identified using questionnaire-based categories	Nursing-associated: 56<br>Personal care workers: 85<br>Other: 107<br><br>Sex<br>Female: 289 (87.0%)<br>Male: 43 (13.0%)<br><br>Age<br>27.7 to <36.6 yrs.: 93 (28.0%)<br>≥36.6 to <42.9 yrs.: 87 (26.2%)<br>≥42.9 to <48.9 yrs.: 96 (28.9%)<br>≥48.9 to 55.8 yrs.: 56 (16.9%)<br><br>Exposure group 2:<br>Nursing jobs identified using questionnaire-based categories: 284<br>Comprised of<br>Clinical nurse in hospital: 97<br>General practice assistant: 36<br>Nursing assistant in hospital: 53<br>Personal care provider, home-based: 32<br>Personal care provider, institution-based: 50<br>Technician in hospital: 16<br><br>Exposure to disinfection tasks in all nursing and related occupations: 180 (54.2%)<br>Exposure to liquid cleaning multi-use products: 183 (55.1%)<br>Exposure to latex glove use:<br>258 (77.7%)<br>Exposure to specific nursing group not given.	following works for at least 3 months during follow up:<br>professional cleaning, disinfecting, nursing, metal working, soldering, welding<br><br>Sex<br>Female: 1420 (57.2%)<br>Male: 1061 (42.8%)<br><br>Age<br>27.7 to <36.6 yrs.: 609 (24.5%)<br>≥36.6 to <42.9 yrs.: 617 (24.9%)<br>≥42.9 to <48.9 yrs.: 609 (24.5%)<br>≥48.9 to 55.8 yrs.: 646 (26.0%)	time in the last 12 months" and „Have you (had) wheezing or whistling when you did not have a cold (in the last 12 months)")<br>and<br>current asthma at the end of the follow-up period (positive response to any of these questions: „Have you had an attack of asthma in the last 12 months", „Have you been woken by an attack of shortness of breath at any time in the last 12 months" or „Are you currently taking any medicine for asthma")<br><br>Classification as symptom-free based on ECRHS I: negative answers to „Have you ever had asthma", „Have you been woken up by an attack of shortness of breath at any time in the last 12 months" and „Have you (had) wheezing or whistling when you did not have a cold (in the last 12 months)"

*Same study population; yrs.: years; SD: standard deviation; ECRHS: European community respiratory health survey; N.A.: not available

Table S4. Characteristics of included studies investigating risk of cleaning-related tasks on asthma (Type B Studies)

Study	Study type	Region	Study period<br>Type of recruitment<br>Population<br>Response (%)	Sex<br>Age<br>Duration of employment	Exposure<br>Exposure assessment	Outcome(s)<br>Outcome assessment
Arif et al. 2009*	Cross-sectional	Texas, USA	2004<br><br>Random sample of four groups of Texas healthcare professionals (HCPs) with active licenses in 2003<br><br>N= 3634<br>Nursing professionals (NPs): 448 comprised of registered nurses (n=394); nurse practitioners (n=14); licensed vocational nurse (n=25); nurse aides (n=15)<br>Other HCPs: 3186<br>comprised of physicians (n=862); respiratory therapists (n=879); occupational therapists (n=968); others (n=477)<br><br>Overall response: 66%<br>Response nursing professionals (NPs): 70%	Sex<br>NPs:<br>Male: 39 (8.8%)<br>Female: 403 (91.2%)<br>Other HCPs:<br>Male: 1114 (35.5%)<br>Female: 2028 (64.5%)<br><br>Age (mean, SD)<br>NPs: 48.6 years, SD 10.7 yrs.<br>other HCPs: 44.9 yrs., SD 12.0 yrs.<br><br>Mean duration of employment:<br>NPs: 23.9 yrs.<br>(SD 11.3, median 24 yrs.)<br>Other HCPs: 18.6 yrs.<br>(SD 11.8, median 16 yrs.)	Cleaning-related tasks (patient care cleaning, instrument cleaning, cleaning of general building surfaces)<br>Use of adhesives/solvents (used in patient care or on general surfaces)<br><br>JEM: exposure dichotomized to<br>0=no probability of exposure<br>1/2=low/high probability of exposure	New-onset asthma:<br>asthma after entry into the healthcare profession constructed from 3 questions:<br>„Have you ever had asthma? "<br>„If yes, has your asthma been confirmed by a doctor? "<br>„If yes, at what age was your asthma confirmed by a doctor? "<br>Age of asthma diagnosis compared to number of years in the healthcare profession to determine temporal relationship between asthma and entry to professional<br><br>Assessment via validated questionnaire
Delclos et al. 2007*	Cross-sectional	Texas, USA	2004<br><br>Random sample of four groups of Texas healthcare professionals (HCPs) with active licenses in 2003<br><br>N=2738<br>physicians: 682 (24.9%)<br>nurses: 695 (25.4%)<br>respiratory therapists: 644 (23.5%)<br>occupational therapists: 717 (26.9%)<br><br>Overall response: 66%	Sex<br>Female:<br>1803 (75.4%)<br>Male:<br>935 (24.6%)<br><br>Mean age<br>46.7±0.32 yrs.<br><br>Number of participants by duration of employment:<br>0-9 yrs.: 689 (25.2%)<br>10-16 yrs.: 706 (25.8%)<br>17-26 yrs.: 675 (24.7%)<br>≥27 yrs.: 668 (24.4%)	Cleaning-related tasks at work (patient care cleaning, instrument cleaning, cleaning of general building surfaces),<br>Use of adhesives/ solvents (used in patient care or on general surfaces)<br><br>JEM: dichotomized by exposed and unexposed JEM-codes for longest held job.<br>If outside health sector, JEM-codes from current job used.	New-onset (reported) asthma:<br>self-reported physician-diagnosed asthma with onset after entry into the health care profession<br>Age of asthma diagnosis compared to number of years in the healthcare profession to determine temporal relationship between asthma and entry to professional<br><br>Assessment via validated questionnaire

Study	Study type	Region	Study period<br>Type of recruitment<br>Population<br>Response (%)	Sex<br>Age<br>Duration of employment	Exposure<br>Exposure assessment	Outcome(s)<br>Outcome assessment
Delclos et al. 2009*	Cross-sectional	Texas, USA	2004<br><br>Random sample of four groups of Texas healthcare professionals (HCPs) with active licenses in 2003<br><br>N= 3650<br>nurses: 926 (25.4%)<br>respiratory therapists: 869 (23.8%)<br>occupational therapists: 947 (25.9%)<br>physicians 852 (23.3%)<br><br>Overall response: 66%	Sex<br>Female: 2391 (67.6%)<br>Male: 1145 (32.4%)<br><br>Mean Age (SD)<br>Asthmatics:<br>45.1 yrs. (11.4 yrs.)<br>Non-asthmatics:<br>45.4 yrs. (12.0 yrs.)<br><br>Mean seniority (SD)<br>Asthmatics:<br>19.1 yrs. (11.5 yrs.)<br>Non-asthmatics:<br><br>19.2 yrs. (12.0 yrs.)	Cleaning-related tasks<br>(patient care cleaning, instrument cleaning, cleaning of general building surfaces)<br>Use of adhesives/ solvents (in patient care or on general surfaces)<br><br>Questionnaire with respect to current and longest held job (self-reports) and Job Exposure Matrix (JEM).	"Asthmatic"<br><br>Self-reported history of physician-diagnosed asthma or wheezing (aside from colds) in the previous 12 months<br><br>Assessment via validated questionnaire
Arif and Delclos, 2012*	Cross-sectional	Texas, USA	2004<br><br>Random sample of four groups of Texas healthcare professionals (HCPs) with active licenses in 2003<br><br>N= 3680<br>physicians: 862 (19.2%)<br>nurses: 941 (73.4%)<br>respiratory therapists: 879 (4.4%)<br>occupational therapists: 968 (3.1%)<br><br>Overall response: 66%	Sex<br>Female: 2437 (76.1%)<br>Male: 1153 (22.3%)<br><br>Mean age 45.3 yrs.,<br>SD 11.9 yrs.	Exposure to cleaning-related chemicals at work<br><br>Self-reported exposures using questionnaire validated for healthcare worker population<br>Exposure in longest held job and any job held for 6 months or longer	WRAS (work related asthma symptoms):<br>asthma symptoms at work (wheezing/whistling or shortness of breath) that get better away from work or worsen on return to work,<br>no history of physician-diagnosed asthma<br>WEA (work exacerbated asthma):<br>asthma symptoms at work that get better away from work or worsen on return to work with history of physician-diagnosed asthma *before* beginning work as HCP<br>OA (occupational asthma):<br>asthma symptoms at work that get better away from work or worsen in return to work with history of physician-diagnosed asthma *after* beginning work as HCP (determined by comparing the age at which a physician made the diagnosis with years spent in the healthcare profession).<br><br>Assessment via validated questionnaires
Gonzalez et al. 2014	Cross-sectional study	France	May 2006 – October 2007<br><br>Multi-center study in 7 French healthcare settings.	Sex<br>Female: 474 (88.9%)<br>Male: 59 (11.1%)	Exposure 1<br>Exposure to cleaning/disinfection-related chemicals at work<br>Assessment via questionnaire and workplace observations	Physician-diagnosed asthma:<br>Positive responses to \`Have you ever had asthma?´ *and* \`Was it confirmed by a doctor?´<br><br>New-onset asthma:

Study	Study type	Region	Study period<br>Type of recruitment<br>Population<br>Response (%)	Sex<br>Age<br>Duration of employment	Exposure<br>Exposure assessment	Outcome(s)<br>Outcome assessment
			Stratified random sampling of the various healthcare departments according to activity (surgery/medicine) and sector (public university hospital/private hospital clinics).<br>All staff members aged 18-65 years, working for at least 6 months in the sampled departments were eligible for inclusion.<br><br>N= 543<br>nurses: 201 (37.0%)<br>auxiliary nurses: 89 (16.4%)<br>cleaners: 94 (17.3%)<br>administrative personnel: 59 (10.9%)<br>others: 100 (18.4%), (mainly comprised of physicians (7.4%),<br>charge nurses (4.1%), physiotherapists (3.1%), and midwives (1.1%))<br><br>Overall response: 77%	Age:<br>39.9 yrs.<br>(SD=10.5)<br><br>Healthcare sector seniority:<br>0-9 yrs.: 197 (37.2%)<br>10-19 yrs.: 133 (25.0%)<br>20+ yrs.: 201 (37.9%)	Exposure 2<br>Cleaning/disinfection tasks performed at work<br>- general disinfection tasks (patient room, furniture surfaces, equipment, instruments…)<br>- general cleaning tasks (floors, surfaces, bathrooms…)<br>- high-risk procedures (product dilution process by manual mixing, use of sprays, placing lids back or not on soaking containers…)<br><br>Assessment via questionnaire	physician-diagnosed asthma but reported onset after entry into the healthcare sector<br><br>Self-administered questionnaire<br>Symptoms and questions related to asthma selected from validated questionnaires such as European Community Respiratory Health Suvey (ECRHS), the International Study of Asthma and Allergies in Childhood (ISAAC), and Epidemiological Study of Genetics and Environment is Asthma (EGEA)
Mirabelli et al. 2007	Cohort study	13 European countries	baseline ECRHS I: 1991<br>follow-up ECRHS II:<br>1998-1999<br><br>Population-based, random sample of adults living close to one of the 28 study centers.<br><br>N= 2813<br>Nursing-related occupations: 332<br>Professional administrative occupations: 2481<br><br>Participation in ECRHS II random sample and in centers for which occupational survey modules were administered: 58%<br><br>Response:	Sex<br><br>Female: 1709 (61%)<br>Male: 1104 (39%)<br><br>Age<br>Nursing-related:<br>27.7 to <36.6 yrs.: 93 (28.0%)<br>≥36.6 to <42.9 yrs.: 87 (26.2%)<br>≥42.9 to <48.9 yrs.: 96 (28.9%)<br>≥48.9 to 55.8 yrs.: 56 (16.9%)<br>Administrative:<br>27.7 to <36.6 yrs.: 609 (24.5%)<br>≥36.6 to <42.9 yrs.: 617 (24.9%)<br>≥42.9 to <48.9 yrs.: 609 (24.5%)<br>≥48.9 to 55.8 yrs.: 646 (26.0%)<br><br>Duration of employment<br>N.A.	Exposure group 1:<br>Work-related cleaning tasks<br><br>Exposure group 2:<br>Exposure to cleaning products at work<br><br>Assessment via questionnaire	New-onset asthma:<br>symptom-free at baseline (negative answers to: „Have you ever had asthma“, „Have you been woken up by an attack of shortness of breath at any time in the last 12 months“ and „Have you (had) wheezing or whistling when you did not have a cold (in the last 12 months)”)<br>and<br>current asthma at the end of the follow-up period (positive response to any of these questions: „Have you had an attack of asthma in the last 12 months“, „Have you been woken by an attack of shortness of breath at any time in the last 12 months“ or „Are you currently taking any medicine for asthma”)<br><br>Classification as symptom-free based on ECRHS I: negative answers to<br>„Have you ever had asthma“, „Have you been woken up by an attack of shortness of breath at any time in the last

Study	Study type	Region	Study period<br>Type of recruitment<br>Population<br>Response (%)	Sex<br>Age<br>Duration of employment	Exposure<br>Exposure assessment	Outcome(s)<br>Outcome assessment
			Baseline<br>(ECRHS I): screening questionnaire: median 78%<br>ECRHS I main questionnaire: median 65%<br>Follow up<br>(ECRHS II) screening questionnaire: median 79%<br>ECRHS II main questionnaire: median 76%<br>Overall response in final study population:<br>nurses (exposed): 87%<br>administrative (unexposed): 57%			12 months" and „Have you (had) wheezing or whistling when you did not have a cold (in the last 12 months)"
Caridi et al. 2019	Cross-sectional	New York City, USA	February 2014<br><br>Members of service Employees International Union Local who lived in New York City and were in one of nine target occupations, providing a range of occupational exposures. 502 union members lacking phone number and mailing address were excluded (from 24,562 members).<br><br>N= 2030<br>nursing assistants: 702 (35%)<br>environmental service workers: 374 (18%)<br>licensed practical nurses: 297 (15%)<br>registered nurses: 280 (14%) laboratory technicians: 166 (8.2%)<br>operating room technicians: 63 (3.1%)<br>respiratory therapists or technicians: 75 (3.7%)<br>central supply workers: 41 (2.0%)<br><br>Response:<br>13%	Sex<br>Female: 1542 (76%)<br>Male: 487 (24%)<br><br>Age<br>Mean: 48.6 yrs.<br>SD=11.4 yrs.<br><br>Duration of employment<br>N.A.	Cleaning of fixed surfaces, sterilization of medical equipment<br><br>Questionnaire with "many" questions from frequently used standardized instruments and a previous study of healthcare workers in Texas.	New-onset (post-hire asthma): physician- diagnosed asthma with onset after entering the healthcare profession<br>Current asthma: physician-diagnosed asthma with any of the following criteria in the last 12 months:<br>attack of asthma, medication use for asthma, hospitalized overnight for asthma, urgent care/treatment for asthma or asthma score ≥1<br><br>Assessment through questionnaire (see exposure assessment)

Study	Study type	Region	Study period<br>Type of recruitment<br>Population<br>Response (%)	Sex<br>Age<br>Duration of employment	Exposure<br>Exposure assessment	Outcome(s)<br>Outcome assessment
Dimich-Ward et al. 2004	Cross-sectional	British Columbia, Canada	Respiratory Therapists (RT): October-December 2000<br>Physiotherapists (PT): January-September 1999<br><br>All registered RTs and PTs working in British Columbia were contacted by mail using database of professional registry.<br><br>N= 903<br><br>Response:<br>RT: 64.1%<br>PT: 68.6%	Sex<br>RT:<br>Female: 161 (58.5%)<br>Male: 114 (41.5%)<br>PT:<br>Female: 573 (91.2%)<br>Male: 55 (8.8%)<br><br>Mean age<br>RT:<br>37.0 yrs. (SD = 7.7 yrs.)<br>PT:<br>43.2 yrs. (SD = 9.2 yrs.)<br><br>Mean duration of employment (SD):<br>RT: 11.3 yrs. (SD = 7.1 yrs.)<br>PT: 17.6 yrs. (SD = 9.6 yrs.)	Sterilization with Glutaraldehyde<br><br>Questionnaire with questions on job tasks and on use of glutaraldehyde	New-onset (reported) asthma (physician-diagnosed asthma since entering the profession)<br><br>Questionnaire with questions on respiratory symptoms based on validated questionnaires from American Thoracic Society-Division of Lung Disease
Ellett et al. 1996	Cross-sectional	USA, all states	March 1995<br><br>5000 surveys sent to 50% of the membership of the ASPAN selected at random<br>ASPAN membership predominantly composed of recovery room nurses<br><br>ASPAN response†: 37%	Sex<br>Female: 1716 (97.9%)<br>Male: 36 (2.1%)<br><br>Mean age<br>ASPAN exposed (E):<br>42.7 yrs.<br>ASPAN not exposed (NE):<br>42.2 yrs.	Use of disinfectants<br><br>Adapted endoscopic disinfectant surveys asking about disinfectant use	Current asthma<br><br>Adapted endoscopic disinfectant surveys asking about health problems.
Dumas, 2021<br><br>USA<br><br>Ref 11184	Cohort	USA and Canada	Recruitment: 2010-2018<br>Follow-up: 2019 (≥ 1 year)<br><br>The Nurses' Health Study 3 (NHS3), a prospective open cohort of nurses in the USA and Canada<br><br>NHS3 study response 86.2%<br><br>Follow-up 63%, with differential selection between those lost to follow-	Sex<br>All females (100%)<br><br>Mean age (SD)<br>Overall:<br>Mean age: 34 yrs.<br>Range: 20-52 yrs.<br>Never or < 1 year exposed:<br>33.6 yrs. (SD = 7.1 yrs.)<br>1-5 years exposed:<br>33.4 yrs. (SD = 7.0 yrs.)	Occupational use of high-level disinfectants (HLDs)<br><br>Questionnaire on HLD use, including duration and type of HLD use	Self-reported clinician-diagnosed asthma in the past 12 months<br><br>Questionnaire

Study	Study type	Region	Study period<br>Type of recruitment<br>Population<br>Response (%)	Sex<br>Age<br>Duration of employment	Exposure<br>Exposure assessment	Outcome(s)<br>Outcome assessment
			up and those remaining in study reported<br><br>N= 17,280<br>Never or <1 year exposed:12,641<br>1-5 years exposed: 2,778<br>>5 years exposed: 1,861	>5 years exposed:<br>38.2 yrs. (S = 5.7 yrs.)		
Patel, 2020<br><br>Ref 7968	Cross-sectional	Texas, USA	2016-2017<br><br>Random sample of certified nurse aides (CNAs) registered through the Texas Department of Aging and Disability Services<br><br>N=413<br>Response: 21.6%	Sex<br>Male: 23 (10.2%)<br>Female: 216 (89.8%)<br><br>Mean age (SD)<br>38.0 yrs. (0.89 yrs)<br><br>Duration of employment<br>0-4 yrs: 69 (35.7%)<br>5-10 yrs: 53 (24%)<br>11-20 yrs: 70 (26.9%)<br>>20 yrs: 47 (13.3%)	Cleaning-related tasks and use of cleaning agents<br><br>Job Exposure Matrix (JEM)	New-onset asthma (physician-diagnosed after entry into health care profession)<br><br>Validated questionnaire
Dumas, 2020<br><br>Ref 7650	Cohort	USA	Baseline: 2009<br>Follow-up: 2011-2015<br><br>Nurses Health Study 2 (NHS2) recruitment of female registered nurses from 14 US states, starting in 1989 with follow-up every 2 years.<br><br>Response for biennial questionnaires: 80%<br><br>N=61 539<br>Follow-up: 98,811/116,429= 84.8% (own calculations)	Sex<br>All females (100%)<br><br>Overall<br>Mean age: 55yrs<br>range 44-68 yrs<br><br>By weekly use of disinfectants, Mean age (SD)<br>none: 55.0 yrs (4.6 yrs)<br>surfaces only: 54.6 yrs (4.6yrs)<br>instruments: 54.1yrs (4.6yrs)<br><br>Duration of employment: N.A.	Occupational exposure to disinfectants:<br>questionnaire on nursing job type, general disinfection tasks, frequency of use<br><br>Cleaning/disinfection tasks: questionnaire<br><br>Use of commonly used disinfectants:<br>job-task-exposure matrix (JTEM)	Self-reported physician-diagnosed asthma in the past year<br><br>Questionnaire

*Same study population †The survey of the Society of Gastroenterology Nurses and Associates (SGNA) was intended to be used as an exposed group, but it used a convenience sample, therefore that population was not included for this analysis.yrs.: years; SD: standard deviation; JEM: Job Exposure Matrix; ECRHS: European community respiratory health survey; ASPAN: American Society of Postanesthesia Nurses; N.A.: not available

Table S5. Results for included studies on asthma in nurses exposed to cleaning/disinfection agents (Type A)

Study	Asthma type	Prevalence or incidence			Risk estimate		
		Effect estimate	Exposure group	Comparison group	Effect estimate	Effect value (95% CI)	Adjusted for
Arif et al. 2009*	New-onset asthma	Prevalence	Nursing professionals (NPs): 44/448 (9.8%)	Other health care professionals (HCPs): 154/3186 (4.8%)	OR**	Other HCPs: 1.00 (Ref.)<br>NPs: 2.14 (1.51-3.05)	No adjustment
Delclos et al. 2007*	New-onset asthma	Prevalence	Nurses: 7.3%<br>Respiratory therapists (RTs): 5.6%<br>Occupational therapists (OTs): 4.5%	Physicians: 4.2%	OR	Physicians: 1.00(Ref.)<br>Nurses: 1.89 (1.18-3.03)<br>RTs: 1.30 (0.78-2.17)<br>OTs: 1.06 (0.63-1.78)	No adjustment
Delclos et al. 2009*	Asthma	Prevalence	Nurses: 300/926 (32.5%)<br>Respiratory therapists (RTs): 331/869 (38.1%)<br>Occupational therapists (OTs): 273/947 (28.8%)	Physicians: 178/947 (18.8%)	PR**	Physicians: 1.00 (Ref.)<br>Nurses: 1.55 (1.32-1.82)<br>RTs: 1.82 (1.56-2.13)<br>OTs: 1.38 (1.17-1.63)	No adjustment
Arif and Delclos, 2012*	Work-related asthma symptoms	Prevalence	Nurses: 35/941 (3.7%)	Physicians: 7/862 (0.8%)	OR**	Physicians: 1.00 (Ref.)<br>Nurses: 4.72 (2.08-10.68)	No adjustment
	Work-exacerbated asthma	Prevalence	Nurses: 11/941 (1.2%)	Physicians: 6 (0.7%)	OR**	Physicians: 1.00 (Ref.)<br>Nurses: 1.68 (0.62-4.58)	No adjustment
	Occupational asthma	Prevalence	Nurses: 9/941 (0.9%)	Physicians: 2 (0.2%)	OR**	Physicians: 1.00 (Ref.)<br>Nurses: 3.69 (0.78-17.41)	No adjustment
Gonzalez et al. 2014	Physician-diagnosed asthma	Prevalence	Nurses: 31/192 (16.1%)<br>Auxiliary nurses: 14/84 (16.7%)	Administrative: 2/59 (3.4%)	OR	Administrative: 1.00 (Ref.)<br>Nurses: 5.49 (1.27-23.7)<br>Auxiliary nurses: 5.70 (1.24-26.12)	No adjustment
	New-onset asthma	Prevalence	Nurses: 11/192 (5.8%)<br>Auxiliary nurses: 5/84 (6.0%)	Administrative: 0 (0%)<br>Others: 3 (1.9%)	OR	Others: 1.00 (Ref.)<br>Nurses: 2.99 (0.83-10.73)<br>Auxiliary nurses: 3.13 (0.74-13.10)	No adjustment
Mirabelli et al. 2007	New-onset asthma	Incidence	Exposure group 1:<br>ISCO-based<br>Nursing and midwifery: 4/86 (4.7%)<br>Nursing-associated: 5/56 (8.9%)<br>Personal care workers, institution-based: 3/85 (3.5%)<br>Other: 8/107 (7.5%)<br><br>Exposure group 2:<br>Reported on questionnaire<br>Clinical nurse in hospital: 3/97 (3.1%)<br>General practice assistant: 2/36 (5.6%)	Professional, administrative: 131/2481 (5.3%)	RR	Professional, administrative: 1.00(Ref.)<br>All nursing and related occupations: 1.16 (0.72-1.87)<br><br>Exposure group 1 (ISCO-88 based):<br>Nursing and midwifery: 0.90 (0.34-2.40)<br>Nursing-associated: 1.85 (0.76-4.50)<br>Personal care workers, institution-based: 0.65 (0.20-2.06)<br>Other: 1.43 (0.70-2.91)<br><br>Exposure group 2 (reported on questionnaire):<br>Clinical nurse in hospital: 0.60 (0.19-1.85)<br>General practice assistant: 1.07 (0.27-4.24)<br>Nursing assistant in hospital: 0.36 (0.05-2.60)	Age, country, sex, and smoking status of ECRHS II

			Nursing assistant in hospital: 1/53 (1.9%)<br>Personal care provider, home-based: 1/32 (3.1%)<br>Personal care provider, institution-based: 6/40 (12.0%)<br>Technician in hospital: 4/16 (25.0%)			Personal care provider, home-based: 0.56 (0.08-3.92)<br>Personal care provider, institution-based: 2.30 (1.03-5.14)<br>Technician in hospital: 4.63 (1.87-11.5)	

OR: odds ratio; PR: prevalence ratio; RR: relative risk; ECRHS: European community respiratory health survey; Ref.: reference

*Same study population

**Own calculation

Table S6. Results for included studies on risk of exposure to cleaning/disinfection agents on asthma in nurses (Type B)

Study	Asthma type	Exposure category	Exposure sub-category	Exposed (%)/ Number exposed cases (%)	Unexposed (%)/ Number of unexposed cases (%)	Effect estimate	Effect value* (95% CI)	Adjusted for
Arif et al. 2009**	New-onset asthma	Cleaning tasks	Patient care cleaning and disinfection	N.A.	N.A.	OR	2.73 (0.34-21.81)	Age, sex, ethnicity, atopy, smoking, BMI and seniority
			Instrument cleaning and disinfection	N.A.	N.A.		1.67 (1.06-2.62)	
			Building surfaces cleaning and disinfection	N.A.	N.A.		1.72 (1.00-2.94)	
		Adhesives/ solvents	Patient care	N.A.	N.A.	OR	1.36 (0.82-2.26)	Age, sex, ethnicity, atopy, smoking, BMI, and seniority
			On surfaces	N.A.	N.A.		1.23 (0.62-2.44)	
Delclos et al. 2007**	New-onset asthma	Cleaning agents	Used in patient care	% Exposed: 2705 (98.8%) / N.A.	% Unexposed: 33 (1.2%)/ N.A.	OR	1.60 (0.18-14.16)	Seniority, race/ethnicity, body mass index, atopy; weighted survey samples
			Instrument cleaning	% Exposed: 1257 (45.9%)/ N.A.	% Unexposed: 1481 (54.1%)/ N.A.		2.22 (1.34-3.67)	
			Surface cleaners	% Exposed: 1943 (71.0%)/ N.A.	% Unexposed: 795 (29.0%)/ N.A.		2.02 (1.20-3.40)	
		Adhesives/ solvents/ gases	Used in patient care	% Exposed: 1921 (70.2%)/ N.A.	% Unexposed: 817 (29.8%)/ N.A.	OR	1.68 (0.99-2.86)	Seniority, race/ethnicity, BMI, atopy; weighted survey samples
			On surfaces	% Exposed: 581 (21.2%)/ N.A.	% Unexposed: 2157 (78.8%)/ N.A.		0.59 (0.26-1.33)	
			Miscellaneous	% Exposed: 869 (31.7%)/ N.A.	% Unexposed: 1869 (68.3%)/ N.A.		0.53 (0.32-0.88)	
Delclos et al. 2009**	Asthma	Cleaning products	Patient care	% Exposed: 64.4%† / N.A.	% Unexposed: 35.6%† / N.A.	PR	JEM: 0.89 (0.52-1.52) Self-reports: 1.26 (1.00-1.59)	Seniority, race/ethnicity, BMI, atopy; weighted survey samples
			Instrument cleaning	% Exposed: 42.2%† / N.A.	% Unexposed: 57.8%† / N.A.		JEM: 1.19 (1.02-1.39) Self-reports: 1.24 (1.06-1.44)	
			Building surfaces	% Exposed: 78.3%† / N.A.	% Unexposed: 21.7%† / N.A.		JEM: 1.32 (1.11-1.56) Self-reports: 1.35 (1.11-1.65)	
		Adhesives/ solvents	Patient care	% Exposed: 31.1%† / N.A.	% Unexposed: 68.9%† / N.A.	PR	JEM: 1.26 (1.06-1.51) Self-reports: 1.34 (1.15-1.56)	Seniority, race/ethnicity, BMI, atopy; weighted survey samples
			On surfaces	% Exposed: 31.1%† / N.A.	% Unexposed: 68.9%† / N.A.		JEM: 0.78 (0.59-1.02) Self-reports: 1.34 (1.15-1.56)	
Arif and Delclos, 2012**	Work-related asthma symptoms	Cleaning agents (in longest held job)	Frequency	% Exposed‡: Never/once a month: 17.6% At least once a day: 13.4%	% Unexposed‡: Never/once a month: 82.4% At least once a day: 86.6%	OR	Never/once a month: 1.00 (Ref.) At least once a week: 2.64 (0.57-12.14)	Age, sex, race/ethnicity, BMI, seniority, atopy

Study	Asthma type	Exposure category	Exposure sub-category	Exposed (%)/ Number exposed cases (%)	Unexposed (%)/ Number of unexposed cases (%)	Effect estimate	Effect value* (95% CI)	Adjusted for
				Every day:<br>32.1%<br>More than once a day:<br>31.8%	Every day:<br>67.9%<br>More than once a day:<br>68.2%		Every day:<br>4.33 (1.14-16.37)<br>More than once a day:<br>5.37 (1.43-20.16)	
		Disinfectants/ sterilants (in longest held job)	Never/once a month	% Exposed‡:<br>Never/once a month: 16.5%<br>At least once a day: 13.1%<br>Every day: 28.3%<br>More than once a day: 37.2%	% Unexposed‡:<br>Never/once a month: 83.5%<br>At least once a day: 86.9%<br>Every day: 61.7%<br>More than once a day: 62.8%	OR	Never/once a month:<br>1.00 (Ref.)<br>At least once a week:<br>1.66 (0.39-7.01)<br>Every day:<br>2.83 (0.79-10.09)<br>More than once a day:<br>2.96 (0.85-10.31)	Age, sex, race/ethnicity, BMI, seniority, atopy
		General purpose cleaning (any job held for 6 months or longer)	Includes bleach, room cleaners, cleaners/abrasives, cleaners for restroom, detergents, disinfectants, ammonia	% Exposed‡:<br>All categories: N.A.<br>Bleach: 55.5%<br>Cleaners/abrasives: 56.4%<br>Disinfectants: 80.9%	% Unexposed‡:<br>All categories<br>N.A.<br>Bleach:<br>45.5%<br>Cleaners/abrasives:<br>43.6%<br>Disinfectants:<br>19.1%	OR	All categories<br>3.93 (1.66-9.32)<br>Bleach:<br>3.72 (1.70-8.12)<br>Cleaners/abrasives:<br>2.50 (1.19-5.25)<br>Disinfectants:<br>2.50 (0.78-8.09)	Age, sex, race/ethnicity, BMI, seniority, atopy
		Instrument cleaning/ sterilization (any job held for 6 months or longer)	Include GA/orthophtaldehyde (OP), formalin/ formaldehyde, chloramines, ethylene oxide	% Exposed‡:<br>All categories: N.A.<br>GA/OP: 38.4%<br>Formalin/formaldehyde:<br>27.2%<br>Chloramines: 9.2%<br>Ethylene oxide: 7.6%	% Unexposed‡:<br>All categories: N.A.<br>GA/OP: 61.6%<br>Formalin/formaldehyde: 72.8%<br>Chloramines: 90.8%<br>Ethylene oxide: 92.4%	OR	All categories:<br>2.14 (1.06-4.32)<br>GA/OP: 2.18 (1.17-4.07)<br>Formalin/formaldehyde:<br>1.48 (0.71-3.07)<br>Chloramines:<br>3.81 (1.79-8.11)<br>Ethylene oxide:<br>2.97 (1.21-7.33)	Age, sex, race/ethnicity, BMI, seniority, atopy
	Work-exacerbated asthma	Cleaning agents	Frequency	See above	See above	OR	Never/once a month:<br>1.00 (Ref.)<br>At least once a day:<br>1.66 (0.28-9.79)<br>Every day:<br>0.74 (0.19-2.99)<br>More than once a day:<br>2.28 (0.56-9.27)	Age, sex, race/ethnicity, BMI, seniority, atopy
		Disinfectants/ sterilants	Never/once a month	See above	See above	OR	Never/once a month:<br>1.00 (Ref.)<br>At least once a day:<br>3.75 (0.35-40.18)<br>Every day:<br>5.06 (0.79-32.48)	Age, sex, race/ethnicity, BMI, seniority, atopy

Study	Asthma type	Exposure category	Exposure sub-category	Exposed (%)/ Number exposed cases (%)	Unexposed (%)/ Number of unexposed cases (%)	Effect estimate	Effect value* (95% CI)	Adjusted for
							More than once a day: 9.02 (1.47-55.35)	
		General purpose cleaning	Includes bleach, room cleaners, cleaners/abrasives, cleaners for restroom, detergents, disinfectants, ammonia	See above	See above	OR	All categories: 1.91 (0.71-5.15) Bleach: 3.13 (1.08-9.08) Cleaners/abrasives: 1.24 (0.42-3.65) Disinfectants: 4.03 (0.86-18.79)	Age, sex, race/ethnicity, BMI, seniority, atopy
		Instrument cleaning/ sterilization	Include GA/orthophtaldehyde (OP), formalin/ formaldehyde, chloramines, ethylene oxide	See above	See above	OR	All categories: 3.40 (1.35-8.53) GA/OP: 1.57 (0.58-4.27) Formalin/formaldehyde: 2.66 (1.03-6.86) Chloramines: 2.02 (0.52-7.89) Ethylene oxide: 2.55 (0.41-15.86)	Age, sex, race/ethnicity, BMI, seniority, atopy
	Occupational asthma	Cleaning agents	Frequency	See above	See above	OR	Never/once a month: 1.00 (Ref.) At least once a day: 0.04 (0.01-0.31) Every day: 0.48 (0.10-2.41) More than once a day: 0.81 (0.17-3.86)	Age, sex, race/ethnicity, BMI, seniority, atopy
		Disinfectants/ sterilants	Never/once a month	See above	See above	OR	Never/once a month: 1.00 (Ref.) At least once a day: 0.04 (0.01-0.31) Every day: 0.52 (0.11-2.38) More than once a day: 0.71 (0.15-3.39)	Age, sex, race/ethnicity, BMI, seniority, atopy
		General purpose cleaning	Includes bleach, room cleaners, cleaners/abrasives, cleaners for restroom, detergents, disinfectants, ammonia	See above	See above	OR	All categories: 1.30 (0.34-4.96) Bleach: 3.22 (0.63-16.53) Cleaners/abrasives: 1.32 (0.40-4.42) Disinfectants: 3.93 (0.75-20.69)	Age, sex, race/ethnicity, BMI, seniority, atopy

Study	Asthma type	Exposure category	Exposure sub-category	Exposed (%)/ Number exposed cases (%)	Unexposed (%)/ Number of unexposed cases (%)	Effect estimate	Effect value* (95% CI)	Adjusted for
		Instrument cleaning/ sterilization	Include GA/orthophtaldehyde (OP), formalin/ formaldehyde, chloramines, ethylene oxide	See above	See above	OR	All categories: 2.25 (0.76-6.66) GA/OP: 1.03 (0.29-3.58) Formalin/formaldehyde: 1.77 (0.55-5.75) Chloramines: 4.81 (1.28-18.06) Ethylene oxide: 1.61 (0.33-7.85)	Age, sex, race/ethnicity, body mass index, BMI, seniority, atopy
Gonzalez et al. 2014	Physician-diagnosed asthma	Occupational exposures	QAC	335/444 (75.4%) / 47 (14.5%)	109/444 (24.6%) / 3 (2.8%)	OR	7.56 (1.84-31.05)	Sex, age, tobacco consumption status, atopy, BMI, latex gloves, and chlorine/bleach
			Chlorine/bleach	258/524 (49.2%) / 31 (12.6%)	266/524 (50.8%) / 26 (10.0%)		1.01 (0.47-2.18)	
			GA	59/304 (19.4%) / 7 (11.9%)	245/304 (80.6%) / 22 (9.3%)		1.31 (0.53-3.23)	No adjustment
		Tasks	Spray use at work	195/526 (37.1%) / 12 (12.3%)	331/526 (62.9%) / 33 (10.3%)	OR	0.84 (0.42-1.69)	Gender, age, tobacco consumption, atopy, BMI, latex gloves, and task
			General disinfection	381/542 (70.3%) / 53 (14.4%)	161/542 (37.1%) / 6 (3.8%)		3.16 (1.17-8.52)	
			Dilution of disinfectants	226/390 (57.9%) / 33 (15.1%)	164/390 (42.1%) / 6 (3.8%)		4.01 (1.34-12.00)	
			General cleaning	367/534 (68.7%) / 50 (14.2%)	167/534 (31.3%) / 9 (5.5%)		2.26 (0.95-5.35)	
			Dilution of cleaning products	227/497 (45.7%) / 25 (11.5%)	270/497 (54.3%) / 29 (11.1%)		0.81 (0.39-1.65)	
			Soaking solution preparation	273/512 (53.3%) / 38 (14.5%)	239/512 (46.7%) / 18 (7.8%)		1.56 (0.77-3.18)	
			Frequency of disinfection tasks	Sometimes by week or every day: 313/511 (61.3%) / 46 (14.7%)	Never or sometimes by month: 198/313 (38.7%) / 12 (6.1%)		2.67 (1.37-5.17)	No adjustment
	New-onset asthma	Occupational exposures	QAC	See above	See above	OR	6.35 (0.83-48.19)	No adjustment
			Chlorine/bleach	See above	See above		2.08 (0.86-5.00)	
			GA	See above	See above		3.01 (0.92-9.86)	
		Tasks	Spray use at work	See above	See above	OR	1.30 (0.56-3.04)	No adjustment
			General disinfection	See above	See above		4.68 (1.08-20.22)	
			Dilution of disinfectants	See above	See above		4.56 (1.00-20.29)	
			General cleaning	See above	See above		3.25 (0.95-11.10)	
			Dilution of cleaning products	See above	See above		1.35 (0.56-3.25)	
			Soaking solution preparation	See above	See above		1.27 (0.53-3.04)	

Study	Asthma type	Exposure category	Exposure sub-category	Exposed (%)/ Number exposed cases (%)	Unexposed (%)/ Number of unexposed cases (%)	Effect estimate	Effect value* (95% CI)	Adjusted for
			Frequency of disinfection tasks	See above	See above		3.13 (1.05-9.35)	
Mirabelli et al. 2007	New-onset asthma	Cleaning products	Ammonia and/or bleach	60/2813 (2.1%) / 7 (11.7%)	2745/2813 (97.9%) / N.A.	RR	2.16 (1.03-4.53)	Age, country, sex, and smoking status of ECRHS II
			Liquid multi-use products	183/2813 (6.5%) / 11 (6.0%)	2630/2813 (93.5%) / N.A.		1.16 (0.61-2.19)	
			Washing powders	78/2813 (2.8%) / 7 (9.0%)	2735/2813 (97.2%) / N.A.		1.65 (0.77-3.53)	
			Any products in spray form	42/2813 (1.5%)/ 5 (11.9%)	2771/2813 (98.5%) / N.A.		2.36 (0.99-5.64)	
		Work tasks	Disinfection	180/2813 (6.4%)/ 12 (6.7%)	2633/2813 (93.6%) / N.A.	RR	1.29 (0.70-2.36)	Age, country, sex, and smoking status of ECRHS II
Caridi et al. 2019	New-onset (post-hire) asthma	Work tasks	Clean fixed surfaces	1291/2030 (63.6%) / 74 (5.7%)	739/2030 (36.4%) / N.A.	OR	1.76 (1.90-2.85)	Age, sex, race, smoking status, history of allergies
			Sterilize medical equipment	301/2030 (14.8%) / 15 (5.0%)	1729/2030 (85.2%) / N.A.		1.36 (0.73-2.51)	
			Use chemicals, adhesives, or solvents on patients	646/2030 (31.2%)/ 40 (6.2%)	1384/2030 (68.2%) / N.A.		1.35 (0.86-2.11)	
	Current asthma	Work tasks	Clean fixed surfaces	1291/2030 (63.6%) / 129 (10%)	739/2030 (36.4%) / N.A.	OR	1.84 (1.26-2.68)	Age, sex, race, smoking status, history of allergies
			Sterilize medical equipment	301/2030 (14.8%) / 25 (8.3%)	1729/2030 (85.2%) / N.A.		1.21 (0.74-2.00)	
			Use chemicals, adhesives, or solvents on patients	646/2030 (31.2%)/ 64 (9.9%)	1384/2030 (68.2%) / N.A.		1.25 (0.88-1.77)	
Dimich-Ward et al. 2004	New-onset asthma	Sterilization with GA	Cold sterilization with GA at least once a month	Exposed: 53.3%† / N.A.	Exposed: 46.7%† / N.A.	OR	3.2 (11.1-9.3)	Age, sex, and smoking status
Ellett et al. 1996	Current asthma	Use of disinfectants	N.A.	929 (53%) / 5%	830 (47%) / 3%	PR	1.66 (1.03-2.67)	Not adjusted
Dumas et al. 2021 Ref 11184	New-onset asthma	HLD use	Duration of use 1	Exposed: 1-5 years: 58 cases >5 years: 57 cases	Unexposed (Never or < 1 year): 276 cases	HR	1-5 yrs.: 0.96 (0.73-1.28) >5 yrs.: 1.38 (1.03-1.85)	Age, race, ethnicity, smoking status, and BMI
			Duration of use 2	Exposed: >5 years: 57 cases	Unexposed (never or ≤5 years): 334 cases	HR	>5 yrs.: 1.39 (1.04-1.86)	
			Number or HLDs currently used	Exposed: No current use: 31 cases	Unexposed (Never or ≤5 years): 334 cases	HR	No current use: 1.46 (1.00-2.12)	

Study	Asthma type	Exposure category	Exposure sub-category	Exposed (%)/<br>Number exposed cases (%)	Unexposed (%)/<br>Number of unexposed cases (%)	Effect estimate	Effect value* (95% CI)	Adjusted for
				Current use of 1 HLD:<br>17 cases<br>Current use of ≥2 HLDs:<br>9 cases			Current use of 1 HLD:<br>1.17 (0.72-1.92)<br>Current use of ≥2 HLDs:<br>1.72 (0.88-3.34)	
			Type of HLDs currently used over more than 5 years	>5 yrs. of use<br>Glutaraldehyde:<br>18 cases<br>Orthophthalaldehyde:<br>4 cases<br>Paracetic acid:<br>1 case<br>Hydrogen peroxide:<br>9 cases<br>Other:<br>4 cases	Unexposed (Never or ≤5 years):<br>334 cases	HR	>5 yrs. of use<br>Glutaraldehyde:<br>1.55 (0.96-2.49)<br>Ortho-phthalaldehyde:<br>1.20 (0.44-3.22)<br>Paracetic acid:<br>-<br>Hydrogen peroxide:<br>1.73 (0.89-3.37)<br>Other:<br>1.12 (0.42-3.01)	
			Duration of HLD use Nurses <34 yrs. old at baseline	>5 years of HLD use:<br>16 cases	Unexposed (Never or ≤5 years):<br>156 cases	HR	1.75 (1.03-2.98)	
			Duration of HLD use Nurses ≥34 yrs. old at baseline	>5 years of HLD use:<br>41 cases	Unexposed (Never or ≤5 years):<br>178 cases	HR	1.26 (0.90-1.78)	
Patel, 2020<br><br>Ref 7968	New-onset asthma (NOA)	Cleaning products and tasks	Cleaning/ disenfection products	Endoscopy: 68 (28.5%)<br>Glutaraldehyde: 68 (28.5%)<br>Orthophthaldehyde: 68 (28.5%)<br>Enzymatic cleaners: 26 (12.1%)<br>Bleach: 216 (90.4%)<br>Quaternary ammonium compounds (QACs): 216 (90.4%)<br>Sprays: 225 (93.2)	Endoscopy: 171 (71.5%)<br>Glutaraldehyde: 171 (71.5%)<br>Orthophthaldehyde: 171 (71.5%)<br>Enzymatic cleaners: 213 (89.1%)<br>Bleach: 23 (9.6%)<br>QACs: 23 9.6%)<br>Sprays: 14 (6.8%)	OR	Endoscopy<br>2.90 (0.79-10.60)<br>GA<br>2.90 (0.79-10.60)<br>Orthophtalaldehyde<br>2.90 (0.79-10.60)<br>Enzymatic cleaners<br>0.84 (0.10-7.02)<br>Bleach/quaternary compounds<br>0.70 (0.08-5.90)	No adjustment
Dumas, 2020<br><br>Ref 7650	New-onset Asthma	Use of Disinfectants	Weekly use of disinfectants to clean surfaces and/or instruments	Any disinfectants cases:170<br>of which:<br>Surface only cases: 97<br>Instruments cases: 73	None: 200 cases	HR	Any disinfectant:<br>1.12 (0.91-1.38)<br>Surface only:<br>1.12 (0.87-1.43)<br>Instruments:<br>1.13 (0.86-1.48)	Age, race, ethnicity, smoking status, BMI
			Specific disinfectants	Formaldehyde<br>cases: 32<br>Glutaraldehyde<br>cases: 108<br>Hypochlorite bleach	N.A.		Formaldehyde<br>0.97 (0.67-1.40)<br>Glutaraldehyde<br>1.11 (0.88-1.41)<br>Hypochlorite bleach	

Study	Asthma type	Exposure category	Exposure sub-category	Exposed (%)/ Number exposed cases (%)	Unexposed (%)/ Number of unexposed cases (%)	Effect estimate	Effect value* (95% CI)	Adjusted for
				cases: 109<br>Hydrogen peroxide<br>cases: 117<br>Quaternary ammonium compounds<br>cases: 124<br>Alcohol<br>cases: 142<br>Enzymatic cleaners<br>cases: 52			1.07 (0.84-1.36)<br>Hydrogen peroxide<br>1.06 (0.84-1.34)<br>Quaternary ammonium compounds<br>1.00 (0.79-1.26)<br>Alcohol<br>1.14 (0.91-1.42)<br>Enzymatic cleaners<br>0.97 (0.72-1.30)	

N.A.: not available; OR: odds ratio; PR: prevalence ratio; RR: relative risk; JEM: Job Exposure Matrix; BMI: body mass index; ECRHS: European community respiratory health survey; GA: Glutaraldehyde; QAC: Quaternary ammonium compounds; HLD: high-level disinfectants

*If not specified, reference group is "not exposed"

**Same study population

†Self-reported exposure prevalence, actual sample sizes not available and they might vary by exposure

‡Information on number of exposed/unexposed cases not available (N.A.)

Table S7. Characteristics of included studies on bronchial hyper-responsiveness (BHR)-related symptoms (Type A)

Study<br><br>Ref [ ]	Study design	Study region	Time and type of recruitment<br><br>Response (%)	Population characteristics			Outcome(s) and outcome assessment
				**Overall**	**Exposure group**	**Comparison group**	
Arif et al. 2009*	Cross-sectional	Texas, USA	2004<br><br>Random sample of four groups of Texas healthcare professionals (HCPs) with active licenses in 2003<br><br>Overall response: 66%<br>Response nursing professionals (NPs): 70%	N= 3634	NPs<br>n= 448<br><br>comprised of<br>registered nurses (RN):<br>n=394<br>nurse practitioners (NPr):<br>n=14<br>licensed vocational nurse (LVN):<br>n=25<br>nurse aides (NA):<br>n=15<br><br>Sex<br>Male: 39 (8.8%)<br>Female: 403 (91.2%)<br><br>Age (mean, SD):<br>NPs: 48.6 years, SD 10.7 yrs.<br><br>Mean duration of employment:<br>23.9 yrs.<br>(SD 11.3, median 24 yrs.)<br><br>NPs working in clinical settings (hospitals, private practice, outpatient clinic, nursing home, public school, home health and dental office)<br>n= 396 (88.4%)<br>Working in non-clinical settings (health department, health insurance agency, research, medical sales, academia and others)<br>n= 52 (11.6%)	Other HCPs:<br>n=3186<br><br>comprised of<br>physicians:<br>n=862<br>respiratory therapists:<br>n=879<br>occupational therapists:<br>n=968<br>others:<br>n=477<br><br>Sex<br>Male: 1114 (35.5%)<br>Female: 2028 (64.5%)<br><br>Age (mean, SD):<br>other HCPs: 44.9 yrs., SD 12.0 yrs.<br><br>Mean duration of employment:<br>18.6 yrs.<br>(SD 11.8, median 16 yrs.)<br><br>Other HCPs working in clinical settings (hospitals, private practice, outpatient clinic, nursing home, public school, home health and dental office)<br>n= 860 (27%)<br>Working in non-clinical settings (health department, health insurance agency, research, medical sales, academia and others)<br>n= 2326 (73%)	BHR-related symptoms:<br>based on 8 questions on asthma and allergy symptoms that had exhibited best combination of sensitivity/specificity when compared to non-specific bronchial challenge testing with methacholine<br><br>Outcome assessment:<br>validated questionnaire

Study<br><br>Ref [ ]	Study design	Study region	Time and type of recruitment<br><br>Response (%)	Population characteristics			Outcome(s) and outcome assessment
				**Overall**	**Exposure group**	**Comparison group**	
Delclos et al. 2007*	Cross-sectional	Texas, USA	2004<br><br>Random sample of four groups of Texas healthcare professionals (HCPs) with active licenses in 2003<br><br>Response<br>overall: 66%<br>occupational therapists: 73%<br>respiratory therapists: 65%<br>nurses: 70%<br>physicians: 54%	N = 2738<br><br>Number of participants by duration of employment:<br>0-9 yrs.:<br>689 (25.2%)<br>10-16 yrs.:<br>706 (25.8%)<br>17-26 yrs.:<br>675 (24.7%)<br>≥27 yrs.:<br>668 (24.4%)<br><br>Sex<br>Female:<br>1803 (75.4%)<br>Male:<br>935 (24.6%)<br><br>Mean age<br>46.7±0.32 yrs.	n= 2056<br>nurses: 695 (33.8%)<br>respiratory therapists: 644 (31.3%)<br>occupational therapists: 717 (34.9%)	Professional group (physicians):<br>n= 682	BHR-related symptoms:<br>based on 8 questions on asthma and allergy symptoms that had exhibited best combination of sensitivity/specificity when compared to non-specific bronchial challenge testing with methacholine, developed in a validation study.<br><br>Outcome assessment:<br>validated questionnaire

*Same study population

yrs.: years; SD: standard deviation; N.A.: not available

Table S8. Characteristics of included studies investigating risk of cleaning-related tasks on bronchial hyper-responsiveness (BHR)-related symptoms (Type B)

Study	Study type	Region	Study period<br>Type of recruitment<br>Population<br>Response (%)	Sex<br>Age<br>Duration of employment	Exposure<br>Exposure assessment	Outcome<br>Outcome assessment
Arif et al. 2009*	Cross-sectional	Texas, USA	2004<br><br>Random sample of four groups of Texas healthcare professionals (HCPs) with active licenses in 2003<br><br>N= 3634<br>Nursing professionals (NPs): 448 comprised of registered nurses (n=394); nurse practitioners (n=14); licensed vocational nurse (n=25); nurse aides (n=15)<br>Other HCPs: 3186<br>comprised of physicians (n=862); respiratory therapists (n=879); occupational therapists (n=968); others (n=477)<br><br>Overall response: 66%<br>Response nursing professionals (NPs): 70%	Sex<br>NPs:<br>Male: 39 (8.8%)<br>Female: 403 (91.2%)<br>Other HCPs:<br>Male: 1114 (35.5%)<br>Female: 2028 (64.5%)<br><br>Age (mean, SD)<br>NPs: 48.6 years, SD 10.7 yrs.<br>other HCPs: 44.9 yrs., SD 12.0 yrs.<br><br>Mean duration of employment:<br>NPs:<br><br>23.9 yrs.<br>(SD 11.3, median 24 yrs.)<br>Other HCPs:<br>18.6 yrs.<br>(SD 11.8, median 16 yrs.)	Cleaning-related tasks (patient care cleaning, instrument cleaning, cleaning of general building surfaces)<br>Use of adhesives/solvents (used in patient care or on general surfaces)<br><br>JEM: exposure dichotomized to<br>0=no probability of exposure<br>1/2=low/high probability of exposure	Bronchial hyper-responsiveness (BHR)-related symptoms:<br>based on 8 questions on asthma and allergy symptoms that had exhibited best combination of sensitivity/specificity when compared to non-specific bronchial challenge testing with methacholine<br><br>Assessment via validated questionnaire
Delclos et al. 2007*	Cross-sectional	Texas, USA	2004<br><br>Random sample of four groups of Texas healthcare professionals (HCPs) with active licenses in 2003<br><br>N=2738<br><br>physicians: 682 (24.9%)<br>nurses: 695 (25.4%)<br>respiratory therapists: 644 (23.5%)<br>occupational therapists: 717 (26.9%)<br><br>Response<br>overall: 66%	Sex<br>Female:<br>1803 (75.4%)<br>Male:<br>935 (24.6%)<br><br>Mean age<br>46.7±0.32 yrs.<br><br>Number of participants by duration of employment:<br>0-9 yrs.:<br>689 (25.2%)<br>10-16 yrs.:<br>706 (25.8%)<br>17-26 yrs.:<br>675 (24.7%)	Cleaning-related tasks at work (patient care cleaning, instrument cleaning, cleaning of general building surfaces),<br>Use of adhesives/ solvents (used in patient care or on general surfaces)<br><br>JEM: dichotomized by exposed and unexposed JEM-codes for longest held job.<br>If outside health sector, JEM-codes from current job used.	BHR-related symptoms:<br>based on 8 questions on asthma and allergy symptoms that had exhibited best combination of sensitivity/specificity when compared to non-specific bronchial challenge testing with methacholine, developed in a validation study.<br><br>Assessment via validated questionnaire

Study	Study type	Region	Study period<br>Type of recruitment<br>Population<br>Response (%)	Sex<br>Age<br>Duration of employment	Exposure<br>Exposure assessment	Outcome<br>Outcome assessment
				≥27 yrs.:<br>668 (24.4%)		
Caridi et al. 2019	Cross-sectional	New York City, USA	February 2014<br><br>Members of service Employees International Union Local who lived in New York City and were in one of nine target occupations, providing a range of occupational exposures. 502 union members lacking phone number and mailing address were excluded (from 24,562 members).<br><br>N= 2030<br>nursing assistants: 702 (35%)<br>environmental service workers: 374 (18%)<br>licensed practical nurses: 297 (15%)<br>registered nurses: 280 (14%) laboratory technicians: 166 (8.2%)<br>operating room technicians: 63 (3.1%)<br>respiratory therapists or technicians: 75 (3.7%)<br>central supply workers: 41 (2.0%)<br><br>Response:<br>13%	Sex<br>Female: 1542 (76%)<br>Male: 487 (24%)<br><br>Age<br>Mean: 48.6 yrs.<br>SD=11.4 yrs.<br><br>Duration of employment<br>N.A.	Cleaning of fixed surfaces, sterilization of medical equipment<br><br>Questionnaire with "many" questions from frequently used standardized instruments and a previous study of healthcare workers in Texas.	BHR-related symptoms: asthma-related symptoms in the past 12 months.<br><br>Assessment through questionnaire (see exposure assessment)
Patel, 2020<br><br>Ref 7968	Cross-sectional	Texas, USA	2016-2017<br><br>Random sample of certified nurse aides (CNAs) registered through the Texas Department of Aging and Disability Services<br><br>N=413<br>Response: 21.6%	Sex<br>Male: 23 (10.2%)<br>Female: 216 (89.8%)<br><br>Mean age (SD)<br>38.0 yrs. (0.89 yrs)<br><br>Duration of employment<br>0-4 yrs: 69 (35.7%)<br>5-10 yrs: 53 (24%)<br>11-20 yrs: 70 (26.9%)	Cleaning-related tasks and use of cleaning agents<br><br>Job Exposure Matrix (JEM)	BHR-related symptoms in the past 12 months<br><br>Validated questionnaire

Study	Study type	Region	Study period<br>Type of recruitment<br>Population<br>Response (%)	Sex<br>Age<br>Duration of employment	Exposure<br>Exposure assessment	Outcome<br>Outcome assessment
				>20 yrs: 47 (13.3%)		

*Same study population yrs.: years; JEM: Job Exposure Matrix; ECRHS: European community respiratory health survey; ASPAN: American Society of Postanesthesia Nurses

Table S9. Results for included studies on bronchial hyper-responsiveness (BHR)-related symptoms (Type A)

	Prevalence or incidence			Risk estimate		
**Study**	**Effect estimate**	**Exposure group**	**Comparison group**	**Effect estimate**	**Effect value (95% CI)**	**Adjusted for**
Arif et al. 2009*	Prevalence	Nursing professionals (NPs): 116/448 (31.3%)	Other health care professionals (HCPs): 715/3186 (72.6%)	PR**	Other HCPs: 1.00 (Ref.)<br>NPs: 1.15 (0.97-1.37)	No adjustment
Delclos et al. 2007*	Prevalence	Nurses: 7.3%<br>Respiratory therapists (RTs): 5.6%<br>Occupational therapists (OTs): 4.5%	Physicians<br>4.2%	OR	Physicians: 1.00 (Ref)<br>Nurses: 1.95 (1.51-2.52)<br>RTs: 2.01 (1.55-2.61)<br>OTs: 2.32 (1.80-2.98)	No adjustment

OR: odds ratio; PR: prevalence ratio; Ref.: reference

*Same study population

**Own calculation

Table S10. Results for included studies on bronchial hyper-responsiveness (BHR)-related symptoms (Type B)

Study	Exposure category	Exposure sub-category	Exposed (%)/ Number exposed cases (%)	Unexposed (%)/ Number of unexposed cases (%)	Effect estimate	Sub-exposure Effect value* (95% CI)	Adjusted for
Arif et al. 2009**	Cleaning tasks	Patient care cleaning and disinfection	N.A.	N.A.	OR	0.69 (0.26-1.83)	Age, sex, ethnicity, atopy, smoking, BMI, and seniority
		Instrument cleaning and disinfection	N.A.	N.A.		1.27 (0.94-1.73)	
		Building surfaces cleaning and disinfection	N.A.	N.A.		1.57 (1.11-2.21)	
	Adhesives/ solvents	Patient care	N.A.	N.A.	OR	1.51 (1.08-2.12)	
		On surfaces	N.A.	N.A.		1.37 (0.91-2.06)	
Delclos et al. 2007**	Cleaning agents	Used in patient care	Exposed: 2705 (98.8%) / N.A.	Unexposed: 33 (1.2%)/ N.A.	OR	0.79 (0.35-1.78)	Seniority, race/ethnicity, BMI, atopy; weighted survey samples
		Instrument cleaning	Exposed: 1257 (45.9%)/ N.A.	Unexposed: 1481 (54.1%)/ N.A.		1.26 (0.93-1.72)	
		Surface cleaners	Exposed: 1943 (71.0%)/ N.A.	Unexposed: 795 (29.0%)/ N.A.		1.63 (1.21-2.19)	
	Adhesives/ solvents/ gases	Used in patient care	Exposed: 1921 (70.2%)/ N.A.	Unexposed: 817 (29.8%)/ N.A.	OR	1.65 (1.22-2.24)	
		On surfaces	Exposed: 581 (21.2%)/ N.A.	Unexposed: 2157 (78.8%)/ N.A.		0.98 (0.64-1.51)	
		Miscellaneous	Exposed: 869 (31.7%)/ N.A.	Unexposed: 1869 (68.3%)/ N.A.		0.78 (0.60-1.01)	
Caridi et al. 2019	Work tasks	Clean fixed surfaces	1291/2030 (63.6%) / 363 (28%)	739/2030 (36.4%) / N.A.	OR	1.38 (1.08-1.77)	Age, sex, race, smoking status, history of allergies
		Sterilize medical equipment	301/2030 (14.8%) / 95 (32%)	1729/2030 (85.2%) / N.A.		1.63 (1.20-2.22)	
		Use chemicals, adhesives, or solvents on patients	646/2030 (31.2%)/ 175 (27%)	1384/2030 (68.2%) / N.A.		1.03 (0.80-1.31)	
Patel, 2020<br><br>Ref 7968	Cleaning products and tasks	Patient care cleaning and disinfection	223 (92.2%)	16 (6.7%)	OR	1.71 (0.45-6.51)	BHR: Race, atopy, obesity, smoking, years at job
		Cleaning/ disenfection products	Endoscopy: 68 (28.5%)<br>Glutaraldehyde: 68 (28.5%)<br>Orthophthaldehyde: 68 (28.5%)<br>Enzymatic cleaners: 26 (12.1%)<br>Bleach: 216 (90.4%)	Endoscopy: 171 (71.5%)<br>Glutaraldehyde: 171 (71.5%)<br>Orthophthaldehyde: 171 (71.5%)<br>Enzymatic cleaners: 213 (89.1%)<br>Bleach: 23 (9.6%)<br>QACs: 23 9.6%)		Endoscopy<br>1.33 (0.66-2.68)<br>GA<br>1.33 (0.66-2.68)<br>Enzymatic cleaners<br>1.23 (0.39-3.86)<br>Bleach<br>1.08 (0.36-3.26)	

Study	Exposure category	Exposure sub-category	Exposed (%)/<br>Number exposed cases (%)	Unexposed (%)/<br>Number of unexposed cases (%)	Effect estimate	Sub-exposure<br>Effect value*<br>(95% CI)	Adjusted for
			Quaternary ammonium compounds (QACs): 216 (90.4%)<br>Sprays: 225 (93.2)	Sprays: 14 (6.8%)		QACs<br>1.08 (0.36-3.26)<br>Sprays<br>1.39 (0.35-5.60)	

N.A.: not available; OR: odds ratio; BMI: body mass index

*If not specified, reference group is "not exposed"

**Same study population

Table S11. Characteristics of included studies investigating risk of work-related and respiratory symptoms (Type B)

**Study**	**Study type**	**Region**	**Study period**<br>**Type of recruitment**<br>**Population**<br>**Response (%)**	**Sex**<br>**Age**<br>**Duration of employment**	**Exposure**<br>**Exposure assessment**	**Outcome(s)**<br>**Outcome assessment**
Vyas et al. 2000	Cross-sectional	United Kingdom	Study years not given, period of assessment 1 year.<br><br>19% of all endoscopy units within the UK were approached (61 in total), all current workers and ex-workers (who left employment within the preceding 5 years due to health problems) in 59 endoscopy units<br><br>N= 416<br><br>nurses in endoscopy units<br>n=348 current workers<br>n=68 ex-employees who had left within the past 5 years<br><br>Response<br>Current workers: 74.4%<br>Ex-employees: 69.2%	Sex<br>Current workers:<br>Female: 319 (91.6%)<br>Male: 29 (8.4%)<br>Ex-employees:<br>Female: 68 (100%)<br>Male: 0<br><br>Age<br>N.A.<br><br>Mean duration of employment<br>Current workforce:<br>2.2 yrs. (range 1 month-19 yrs.)<br>74.1% employed for < 5 yrs.<br>Ex-workers:<br>N.A.	Exposure to glutaraldehyde (GA)<br><br>Single work environment questionnaire at each unit (completed by senior member of nursing staff).<br>Site inspection confirmed the work environment and personal work practices in used.<br>Measurements for airborne concentrations of aldehyde biocides.	Work-related symptoms (WRS): symptoms improving on rest days or symptoms experienced as more severe during a work shift including chronic bronchitis, persistent cough, chest tightness, shortness of breath, and wheeze. Lower respiratory tract symptoms (any one of the above stated WRS).<br><br>For current employees symptom screening questionnaire (adaption of Medical Research Council respiratory questionnaire) for lower respiratory tract.<br>For ex-employees questions about past WRS had been present and they were continued
Dimich-Ward et al. 2004	Cross-sectional	British Columbia, Canada	Respiratory Therapists (RT): October-December 2000<br>Physiotherapists (PT): January-September 1999<br><br>All registered RTs and PTs working in British Columbia were contacted by mail using database of professional registry.<br><br>N= 903<br><br>Response:<br>RT: 64.1%<br>PT: 68.6%	Sex<br>RT:<br>Female: 161 (58.5%)<br>Male: 114 (41.5%)<br>PT:<br>Female: 573 (91.2%)<br>Male: 55 (8.8%)<br><br>Mean age<br>RT:<br>37.0 yrs. (SD = 7.7 yrs.)<br>PT:<br>43.2 yrs. (SD = 9.2 yrs.)<br><br>Mean duration of employment (SD):<br>RT: 11.3 yrs. (SD = 7.1 yrs.)	Sterilization with Glutaraldehyde<br><br>Questionnaire with questions on job tasks and on use of glutaraldehyde	Respiratory symptoms at any time in the last 12 months. Terms used: asthma attack, wheeze, chest tightness, woken by cough, woken by dyspnea. Terms for other respiratory symptoms included; usual cough, usual phlegm, childhood asthma (physician-diagnosed asthma before age of 16 years), reported asthma (physician-diagnosed asthma since entering the profession)<br><br>Questionnaire with questions on respiratory symptoms based on validated questionnaires from American Thoracic Society-Division of Lung Disease

Study	Study type	Region	Study period<br>Type of recruitment<br>Population<br>Response (%)	Sex<br>Age<br>Duration of employment	Exposure<br>Exposure assessment	Outcome(s)<br>Outcome assessment
				PT: 17.6 yrs. (SD = 9.6 yrs.)		
Ellett et al. 1996	Cross-sectional	USA, all states	March 1995<br><br>5000 surveys sent to 50% of the membership of the ASPAN selected at random<br><br>ASPAN membership predominantly composed of recovery room nurses<br><br>Response†:<br>ASPAN: 37%	Sex<br>Female: 1716 (97.9%)<br>Male: 36 (2.1%)<br><br>Mean age<br>ASPAN exposed (E):<br>42.7 yrs.<br>ASPAN not exposed (NE):<br>42.2 yrs.	Use of disinfectants<br><br>Adapted endoscopic disinfectant surveys asking about disinfectant use	Respiratory problems (not defined)<br><br>Adapted endoscopic disinfectant surveys asking about health problems.

yrs.: years; SD: standard deviation; ASPAN: American Society of Postanesthesia Nurses; N.A.: not available

Table S12. Results of studies with work-related symptoms and respiratory symptoms (Type B)

Study	Exposure category	Exposed (%)/ Number exposed cases (%)	Unexposed (%)/ Number of unexposed cases (%)	Outcome	Effect estimate	Sub-exposure Effect value* (95% CI)	Adjusted for
Vyas et al. 2000	Exposure to peak Gluteraldehyde concentrations on current workers only	N.A.	N.A.	Chronic bronchitis	RR	1.6 (1.25-2.05)	Smoking, months worked on the unit, number of hours spent on the unit, and number of hours spent decontaminating endoscopes
				Persistent cough		0.76 (0.54-1.06)	
				Wheeze		0.52 (0.32-0.83)	
				Chest tightness		0.94 (0.69-1.28)	
				Shortness of breath		1.51 (0.97-2.36)	
				Lower respiratory tract symptoms (any of the above symptoms)		1.03 (0.80-1.32)	
Dimich-Ward et al. 2011	Sterilization with Gluteraldehyde	% unexposed: 53.3% / N.A.	% unexposed: 46.7% / N.A.	Asthma attack	OR	1.3 (0.6-2.9)	Age, sex, childhood asthma, and smoking status
				Wheeze		2.1 (1.1-3.8)	
				Chest tightness		1.4 (0.7-2.8)	
				Woken by cough		2.3 (1.3-3.9)	
				Woken by dyspnea		1.3 (0.6-3.1)	
				Usual phlegm		1.0 (0.4-2.4)	
				Usual cough		1.5 (0.6-3.5)	
Ellett et al. 1996	Use of disinfectants	929 (53%)/ 4%	830 (47%)/ 3%	Respiratory symptoms (not defined)	PR	1.32 (0.80-2.18)	Not adjusted

N.A.: not available; OR: odds ratio; PR: prevalence ratio; RR: relative risk

*If not specified, reference group is "not exposed"

Table S13. Risk of bias of included studies investigating work-related symptoms and respiratory symptoms

Study ID	Major domains						Minor domains			OVERALL
	Recruitment procedure and follow-up	Exposure definition and measurement	Outcome source and validation	Confounding	Analysis method	Chronology	Assessor blinding	Funding	Conflict of interest	
**Type B (Risk of exposure to cleaning and disinfectants in nurses)**										
Vyas et al. 2000	✓	✗	✗	✗	✓	✗	⚠	⚠	⚠	✗
Dimich-Ward et al. 2011	✓	✗	✗	✗	✓	✗	⚠	⚠	⚠	✗
Ellett et al. 1996	✓	✓	✗	✗	✗	✗	⚠	⚠	⚠	✗

✓ Low Risk; ⚠ Unclear; ✗ High risk;

Table S14. Characteristics of included studies investigating risk of chronic obstructive pulmonary disease (COPD) (Type B)

Study	Study type	Region	Study period<br>Type of recruitment<br>Population<br>Response (%)	Sex<br>Age<br>Duration of employment	Exposure<br>Exposure assessment	Outcome(s)<br>Outcome assessment
Dumas, 2019<br><br>Ref 8405	Cohort	USA	Baseline: 2009<br>Follow-up: 2011-2015<br><br>Nurses Health Study 2 (NHS2) recruitment of female registered nurses from 14 US states, starting in 1989 with follow-up every 2 years.<br><br>Response for cycles >90%<br><br>N=73,262<br>Follow-up: 98,817/116,429 =84.8% (own calculations)	Sex<br>All females (100%)<br><br>Mean age (SD)<br>Overall<br>54.7 yrs (4.6 yrs)<br>By weekly use of disinfectants<br>none: 55.0 yrs (4.6 yrs)<br>surfaces only: 54.5 yrs (4.6yrs)<br>instruments: 54.1yrs (4.6yrs)<br><br>Duration of employment: N.A:	Frequency of cleaning or disinfection tasks<br>Use of commonly-used disinfectants<br><br>Cleaning/disinfection tasks: questionnaire<br><br>Use of commonly used disinfectants:<br>job-task-exposure matrix (JTEM)	Self-reported physician-diagnosed COPD<br>COPD stringent case definition for meta-analysis via validated questionnaire<br><br>Questionnaire

Table S15. Results of included studies investigating risk of chronic obstructive pulmonary disease (COPD) (Type B)

Study	Asthma type	Exposure category	Exposure sub-category	Exposed (%)/ Number exposed cases (%)	Unexposed (%)/ Number of unexposed cases (%)	Effect estimate	Effect value* (95% CI)	Adjusted for
Dumas, 2019<br><br>Ref 8405	COPD	Use of Disinfectants	Weekly use of disinfectants to clean surfaces and/or instruments	Any disinfectants cases: 306<br>of which:<br>Surface only:<br>cases: 161<br><br>Instruments: 145<br><br>More stringent case definition:<br>Any disinfectants: 138 cases<br>Surface only: 75 cases<br>Instruments: 63 cases	Unexposed cases (no weekly use): 276<br><br>More stringent case definition:<br>Unexposed cases (no weekly use): 120	HR	Any disinfectant:<br>1.35 (1.14-1.59)<br>Surface only:<br>1.38 (1.13-1.68)<br>Instruments:<br>1.31 (1.07-1.61)<br><br>More stringent case definition:<br>Any disinfectant:<br>1.24 (0.96-1.59)<br>Surface only:<br>1.32 (0.99-1.78)<br>Instruments:<br>1.14 (0.84-1.56)	Age, smoking status, pack-years, race, ethnicity, BMI
			Frequency of any disinfectants use	<1 day/week: 113 cases<br>1-3 days/week: 165 cases<br>4-7 days/week: 140 cases	Never: 163 cases		<1 day/week: 1.12 (0.88-1.42)<br>1-3 days/week: 1.40 (1.12-1.74)<br>4-7 days/week: 1.43 (1.13-1.80)	
			Frequency of spray use	1 day/week: 113 cases<br>1-3 days/week: 165 cases<br>4-7 days/week: 140 cases	Never: 147 cases		<1 day/week: 1.26 (0.94-1.69)<br>1-3 days/week: 1.35 (0.95-1.91)<br>4-7 days/week: 1.38 (0.93-2.03)	
			Frequency of disinfectant use for cleaning surfaces	1 day/week: 116 cases<br>1-3 days/week: 152 cases<br>4-7 days/week: 128 cases	Never: 185 cases		<1 day/week: 1.09 (0.86-1.38)<br>1-3 days/week: 1.31 (1.05-1.63)<br>4-7 days/week: 1.37 (1.09-1.72)	

Study	Asthma type	Exposure category	Exposure sub-category	Exposed (%)/<br>Number exposed cases (%)	Unexposed (%)/<br>Number of unexposed cases (%)	Effect estimate	Effect value* (95% CI)	Adjusted for
			Frequency of disinfectant use for cleaning instruments	1 day/week: 110 cases<br>1-3 days/week: 82 cases<br>4-7 days/week: 62 cases	Never: 185 cases		<1 day/week: 1.25 (1.01-1.55)<br>1-3 days/week: 1.16 (0.91-1.49)<br>4-7 days/week: 1.35 (1.03-1.78)	
			Specific disinfectants	Formaldehyde<br>cases: 62<br>Glutaraldehyde<br>cases: 192<br>Hypochlorite bleach<br>cases: 215<br>Hydrogen peroxide<br>cases: 229<br>Quaternary ammonium compounds<br>cases: 233<br>Enzymatic cleaners<br>cases: 97	N.A.		Formaldehyde<br>1.20 (0.92-1.57)<br>Glutaraldehyde<br>1.25 (1.04-1.51)<br>Hypochlorite bleach<br>1.36 (1.13-1.64)<br>Hydrogen peroxide<br>1.29 (1.08-1.54)<br>Quaternary ammonium compounds<br>1.33 (1.11-1.60)<br>Enzymatic cleaners<br>1.05 (0.84-1.31)	

Table S16. Risk of bias of study included investigating chronic obstructive pulmonary disease (COPD)

Study ID	Major domains						Minor domains			OVERALL
	Recruitment procedure and follow-up	Exposure definition and measurement	Outcome source and validation	Confounding	Analysis method	Chronology	Assessor blinding	Funding	Conflict of interest	
**Type B (Risk of exposure to cleaning and disinfectants in nurses)**										
Dumas et al. 2019†	✅	✅ ❌	❌	❌	✅	✅	⚠️	⚠️	❌	❌

✅ Low Risk; ⚠️ Unclear; ❌ High risk; †Exposure low risk for "disinfection tasks" /high risk for "specific disinfectants
